# Ciliary proteins Fap43 and Fap44 interact with each other and are essential for proper cilia and flagella beating

**DOI:** 10.1007/s00018-018-2819-7

**Published:** 2018-04-23

**Authors:** Paulina Urbanska, Ewa Joachimiak, Rafał Bazan, Gang Fu, Martyna Poprzeczko, Hanna Fabczak, Daniela Nicastro, Dorota Wloga

**Affiliations:** 1 Laboratory of Cytoskeleton and Cilia Biology, Department of Cell Biology, Nencki Institute of Experimental Biology PAS, Pasteur 3, 02-093 Warsaw, Poland; 2 Departments of Cell Biology and Biophysics, University of Texas Southwestern Medical Center, 6000 Harry Hines Blvd., Dallas, TX USA

**Keywords:** Wdr52, Wdr65, Wdr96, Dyh6, Dyh7, Tether/tether head complex

## Abstract

Cilia beating is powered by the inner and outer dynein arms (IDAs and ODAs). These multi-subunit macrocomplexes are arranged in two rows on each outer doublet along the entire cilium length, except its distal end. To generate cilia beating, the activity of ODAs and IDAs must be strictly regulated locally by interactions with the dynein arm-associated structures within each ciliary unit and coordinated globally in time and space between doublets and along the axoneme. Here, we provide evidence of a novel ciliary complex composed of two conserved WD-repeat proteins, Fap43p and Fap44p. This complex is adjacent to another WD-repeat protein, Fap57p, and most likely the two-headed inner dynein arm, IDA I1. Loss of either protein results in altered waveform, beat stroke and reduced swimming speed. The ciliary localization of Fap43p and Fap44p is interdependent in the ciliate *Tetrahymena thermophila*.

**Electronic supplementary material:**

The online version of this article (10.1007/s00018-018-2819-7) contains supplementary material, which is available to authorized users.

## Introduction

Cilia and their homologous structures, flagella, are evolutionarily conserved organelles that convert the chemical energy released during ATP hydrolysis into a mechanical force to power cell motility or generate fluid flows at the epithelial cell surface. The canonical motile cilium (this term will be used hereafter for both cilia and flagella) consists of a 9 + 2 microtubular core, called the axoneme, and multi-subunit complexes, such as the outer and inner dynein arms (ODAs and IDAs), radial spokes (RSs), the nexin–dynein regulatory complex (N–DRC) and the modifier of inner dynein arms (MIA) complex. These complexes are periodically distributed along the outer doublet microtubules and form a characteristic pattern repeating every 96 nm, called the 96-nm axonemal repeat unit [1–3]. Each 96-nm repeat contains four outer and seven inner dynein arms (a–g) attached to the A-tubule of the outer doublet and extending their motor domains towards the B-tubule of the preceding doublet. In contrast to two-headed (or, in some organisms, three-headed) ODAs, which are periodically distributed every 24 nm and share similar architecture and protein composition, the IDAs are heterogeneous in their structure and subunit composition, and most likely also in their functions [2, 4–9].

Among IDAs, only the most proximal arm of the 96-nm ciliary unit has a two-headed heterodimeric structure (IDA f or I1), while the remaining six IDAs are single-headed [2, 6]. The two-headed IDA I1 forms a trilobed structure consisting of two dynein heavy chains that differ in their function and distance from the surface of the A-tubule [6, 10, 11] and three intermediate and five light chains (ICLC) that form the third lobe [for review, 12, 13]. Cryo-electron tomography and 3D reconstruction analyses revealed that IDA I1 is highly connected with other axonemal structures [13]. Importantly, the existence of linker structures between outer and inner dynein arms suggests a possible cross-talk between these structures [2, 8, 14].

The molecular mechanism that regulates cilia and flagella beating is still an intriguing and challenging puzzle. At the ultrastructural level, cilia beating is generated by the spatiotemporally coordinated, ATP-dependent motor activity of the dynein heavy chains of the inner and outer dynein arms. However, a phenotypic analysis of numerous IDA and ODA mutants revealed that inner and outer dynein arms perform different functions and that their activities are regulated differently. It is assumed that the inner dynein arms control the size and waveform of the cilia bend and that their activity is regulated by the mechano-chemical signals initiated at the central pair complex and transmitted to IDAs by the radial spokes. The two-headed IDA I1 is suggested to contribute to the formation of the waveform through the modulation of flagellar bending [9], possibly through changes in the level of phosphorylation of intermediate chain IC138, a subunit of the ICLC structure [4, 15–17]. Interestingly, mutations in IDA I1 can rescue the immotility caused by defects in the cilia central pair complex [5, 18]. In contrast to IDAs, ODAs control the cilia beat frequency and are downstream of IDAs in the signal transduction cascade [4, 7, 16, 19].

The detailed molecular mechanism(s) that regulate(s) cilia beating is/are not fully understood. Among other reasons, this is due to the incomplete identification of the proteins involved in this process and the factors that regulate their interactions, such as second messengers or posttranslational modifications. The assembly and proper function of cilia require several hundred proteins [20, 21], but the roles and precise localization (at the ultrastructural level) of a substantial fraction of putative ciliary proteins remain unknown. At the same time, 3D reconstructions using cryo-electron tomography revealed that each axonemal unit contains, in addition to established macromolecular complexes, numerous minor structures of unknown protein composition and function [2, 13, 22–26]. These minor complexes may function as links between the major complexes and/or as their regulators. Some of these minor complexes may even play a role in the regulation of dynein arm activity. Thus, a better understanding of the mechanisms that regulate cilia motility requires the identification of all proteins involved in this process. Despite the substantial progress in localizing and deciphering the roles of new ciliary proteins involved in regulation of cilia beating, such as the recent discoveries of the CSC [27–30] and MIA complexes [3], we are still far from decoding the molecular mechanisms that govern cilia motility.

Here, we present evidence that two highly evolutionarily conserved proteins, Fap43p and Fap44p, localize exclusively to cilia and are indispensable for proper cilia motility in the ciliate *Tetrahymena thermophila*. Deletion of either Fap43p or Fap44p results in abnormal cilia beating and reduced cell swimming velocity. In cilia, both proteins localize in close proximity to each other and to Fap57p, a large ciliary protein of unknown function, and most likely to IDA I1. Moreover, Fap44p is missing from *FAP43*-*KO* mutant cilia, and likewise, Fap43p is absent from cilia lacking Fap44p. Thus, Fap43p and Fap44p are most likely subunits of a minor protein complex that is positioned in close proximity to IDA I1 and may regulate its activity.

## Materials and methods

### Strains, culture and phenotypic studies

The wild-type CU428.2 and B2086.2 cells were obtained from the *Tetrahymena* Stock Center (Cornell University, Ithaca, NY, US), and the CU522 strain, which carries a mutation in the macronuclear *BTU1* locus, causing paclitaxel sensitivity, was kindly provided by Dr. Jacek Gaertig (University of Georgia, Athens, GA, USA). The cells were grown at 30 °C in SPP (Super Proteose Peptone) medium [31] with moderate shaking (80 rpm). After transformation by biolistic bombardment, positive clones were selected by growing in SPP medium supplemented with an antibiotic–antimycotic mix (Sigma-Aldrich, St. Louis, MO, USA) and the appropriate selection drug.

The cell proliferation rate, swimming speed and phagocytosis rate were determined as previously described [30]. Cilia motility was visualized in *Tetrahymena* cells that were partly immobilized in 10% Ficoll 400 in 10 mM Tris–HCl, pH 7.5, in a chamber made on a slide coated with 0.1% poly-l-lysine [32]; cilia beating was recorded using a high-speed camera (Andor Zyla 5.5 sCMOS) mounted on a Leica DMI 6000 microscope (63 × oil immersion lens, numerical aperture 1.4) with an Andor DsD2 unit. The frame rate of the video recording was 200 frames/s.

*Chlamydomonas fap44*, *fap43* and *fap244* mutants and the control strain (wild-type) cc-4533 were generated by the *Chlamydomonas* Library Project (CLiP) [33, 34] and purchased from the *Chlamydomonas* Resource Center (http://www.chlamycollection.org). After streaking the cells on TAP (Tris–Acetate–Phosphate) media agar plates, single colonies were picked and transferred into 24-well plates with liquid TAP medium and cultured at 23 °C under 12:12 h light/dark conditions.

For genomic DNA analyses, cells were centrifuged at 3000 rpm, washed with ddH_2_O, and resuspended in 20–50 µl ddH_2_O. After the addition of an equal volume of 100% ethanol and 200 µl of 5–10% (w/v) Chelex-100 (Bio-Rad, CA, USA), the solution was heated for 10 min at 98 °C. After centrifugation at 10,000×*g* for 10 min, the supernatant was used for genotyping (Phire Plant Direct PCR kit, Thermo Fisher Scientific, Waltham, MA, USA) with the following primers: for the *fap44* strain, the gene-specific primer *fap44*-1 (5′-GAAGGCATAATGGCTGGTGT-3′), inserted cassette primer Aph8-1(5′-GCTCGTGGAGCTCTGAATCT-3′), and control primers (5′-GAAGGCATAATGGCTGGTGT-3′) and (5′-GACGGGCAACGAGTCCTCGC-3′); for the *fap43* strain, the gene-specific primers *fap43*-1 (5′-CTTCAAAAAGGAGTTTGCGG-3′) and *fap43*-2 (5′-ACAGCCTTGGACCTTCCTTT-3′) and control primers (5′-AGCAGCTTCTACCTTCTGCG-3′) and (5′-GTGGTGCGGAGAAATGAGAT-3′); and for the *fap244* strain, the gene-specific primers *fap244*-1 (5′-CACACGTCCATCTGGTTGTC-3′) and *fap244*-2 (5′-GCCATGCTGCTTTCTAGTCC-3′) and control primers (5′-GGTATAATGCGGCGTTCTGT-3′) and (5′-TCAATCTGTGCCTGCATCTC-3′). For the swimming speed analysis, cells were placed between a glass slide and cover slide separated by a Vaseline cushion, and DIC images of cells were recorded using a 60 × oil objective lens with a Nikon ECLIPSELVDIA-N microscope equipped with an ANDOR Zyla-VSC-04868 camera. Videos (~ 400 fps) of freely swimming cells were acquired using a 20 × objective and dark-field settings. The motility of cells was tracked with Nikon NIS-Elements AR software, and the swimming speeds were calculated based on the tracked distance and elapsed time. Statistical significance was determined by Student’s *t* test.

### Protein tagging and domain analysis

The *Tetrahymena thermophila FAP43* (TTHERM_00196190) and *FAP44* (TTHERM_00498220) gene sequences were obtained from the *Tetrahymena* Genome Database (http://www.ciliate.org). The DNA fragments with the addition of the appropriate restriction sites used to prepare expression plasmids were amplified from the genomic DNA using Phusion Hot Start II high-fidelity DNA polymerase (Thermo Fisher Scientific, Waltham, MA, USA) and the primers listed in Table S1. To express C-terminally 3xHA-tagged Fap43p or Fap44p in their native loci, we used a strategy similar to one described previously [35]. Generally, approximately 1 kb of the open reading frame (ORF) and 1 kb of the 3′UTR were cloned into an appropriate native locus expression vector [35]. The constructed pFAP43–3HA and pFAP44–3HA plasmids enabled the expression of either Fap43p or Fap44p with the C-terminal 3HA tag under the control of the corresponding native promoter and transcription termination with 0.6 kb of *BTU1* 3′UTR. The neo4 cassette, enabling resistance of the transformed *Tetrahymena* cells to paromomycin [36], was inserted in the reverse orientation between the 3′UTR of *BTU1* and the 3′UTR of the analyzed gene.

To express Fap43p with a C-terminal GFP tag under the control of its native promoter in the native locus, the 3HA tag from pFAP43–3HA native locus expression plasmids was replaced by a GFP coding region preceded by a 27-nucleotide linker encoding GSGGGSGTG amino acid residues.

To express C-terminally 2V5-tagged Fap43p or Fap44p under the control of their native promoters in their native loci, the 3HA tag from the pFAP43–3HA or pFAP44–3HA native locus expression plasmid was removed and replaced by a 2V5 coding region preceded by a 27-nucleotide linker, and the neo4 cassette was replaced by a cassette providing resistance of the transformed *Tetrahymena* cells to puromycin [37]. The pFAP57A–2V5 native locus expression plasmid was generated by replacing the *FAP44* gene fragment in the pFAP44–2V5 native locus expression plasmid with a fragment of the open reading frame and 3′UTR fragment of *FAP57A*, amplified using primers listed in Table S1.

To express C-terminally HA–BirA*-tagged Fap43p or Fap44p under the control of their native promoter in the native loci, the 3xHA tag from the pFAP43–3HA or pFAP44–3HA native locus expression plasmids was removed and replaced by a *Tetrahymena*-optimized BirA* coding region (kindly provided by Dr. Jacek Gaertig) preceded by a 27-nucleotide linker encoding the amino acid residues GSGGGSGTG and a single HA coding region.

Approximately 10 μg of the final plasmid was digested with *Mlu*I and *Xho*I to separate the targeting fragment from the plasmid backbone, precipitated onto DNAdel Gold Carrier Particles (Seashell Technology, La Jolla, CA, USA) according to the manufacturer’s instructions and biolistically transformed into CU428.2 cells or mutant cells. Transformants were selected for 3–4 days at 30 °C on SPP with 2.5 μg/ml CdCl_2_ and either 100 μg/ml paromomycin (BioShop Canada Inc.) or 200 μg/ml puromycin (BioShop Canada Inc.) The positive clones were grown in media with decreasing concentrations of CdCl_2_ (to 0.05–0.1 μg/ml) and either an increasing concentration of paromomycin or a constant concentration of puromycin to promote phenotypic assortment.

To overexpress Fap43p, the predicted ORF of *FAP43* was amplified with the addition of *Mlu*I and *Bam*HI sites at the 5′ and 3′ ends, respectively (primers in Table S1), and cloned into either the pMTT1–GFP vector [38] or the pMTT1-HA vector [30], enabling protein expression as an N-terminally GFP- or HA-tagged fusion protein under the control of a cadmium-dependent *MTT1* promoter from a non-essential *BTU1* locus. To overexpress Fap44p in its native locus, approximately 1.2 kb of the predicted ORF of *FAP44,* starting from the ATG codon, was amplified with the addition of *Mlu*I and *Bam*HI sites at the 5′ and 3′ ends, respectively, and cloned into a pMTT1–GFP vector [38]. Next, the 5′UTR *BTU1* fragment was removed using *Sac*II and *Cla*I sites and replaced by 0.8 kb 5′UTR of *FAP44*, amplified with the addition of *Sac*II and *Pst*I sites at the 5′ and 3′ ends, respectively, and a neo2 cassette [39], amplified with the addition of *Pst*I and *Cla*I sites. Thus, the neo2 cassette was inserted in the reverse orientation between the 5′UTR of *FAP44* and the *MTT1* promoter. The transgene was separated from the plasmid backbone using *Sac*II and *Bam*HI.

For domain truncation analyses, fragments of the *FAP43* or *FAP44* ORFs were amplified with the addition of the *Mlu*I and *Bam*HI restriction sites (primers in Table S1) and cloned into a pMTT1-HA plasmid to enable the expression of protein with C-terminal HA tag [40].

Approximately 15–20 μg of the obtained plasmid was digested with *Apa*I and *Sac*II to separate the constructs from the plasmid backbone and used to biolistically transform CU522 cells. The transformed cells were selected for 3–4 days on SPP medium supplied with 20 μM paclitaxel (BioShop Canada Inc.).

### Deletion of *Tetrahymena FAP43* and *FAP44* gene fragments

A construct to disrupt the *FAP43* coding region was prepared using a pNeo4 plasmid [36]. Briefly, two fragments of the gene, separated by 0.62 kb, were amplified by PCR with the addition of *Apa*I and *Sma*I sites for the upstream 1.77 kb fragment and *Pst*I and *Sac*II restriction sites for the 1.52 kb downstream fragment and cloned into a pNeo4 plasmid such that the neo4 cassette was in reverse orientation to *FAP43* (primers in Table S1). For biolistic transformation of the conjugating CU428.2 and B2086.2 cells, 60 μg of the obtained plasmid was digested with *Apa*I and *Sac*II restriction endonucleases and coated onto gold particles. The conjugating cells (10^7^ cells) were transformed with three shots (20 μg per shot). The *FAP43* heterokaryons were generated as described [41, 42]. The deletion of the 0.62-kb fragment of the *FAP43* gene was confirmed by PCR with the primers listed in Table S1.

To engineer cells lacking a fragment of *FAP43*, *FAP44*, *DYH6* or *DYH7* in the macronuclear genome, a 0.6–1-kb fragment encompassing a part of the 5′UTR and ORF of the gene was amplified by PCR with the addition of pcoDel plasmid flanking sequences [43] and cloned into a pcoDel plasmid (kindly provided by Dr. Mochizuki) digested with *Not*I using the Gibson method [44] and the OverLap Assembly Kit (A&A Biotechnology, Poland). A 10 µg sample of the obtained FAP43, FAP44, DYH6 or DYH7 coDel plasmid was used to biolistically transform the conjugating CU428.2 and B2086.2 cells, and transformants were selected for 3–4 days on SPP medium supplied with 100 μg/ml paromomycin. Among the paromomycin-resistant clones, cells from slow-swimming clones (suggesting defects in cilia function) were selected, and the targeted loci were analyzed by PCR [43]. The genomic DNA was purified, and the size of the deleted fragment was estimated by PCR using primers complementary to the genomic sequences positioned approximately 1 kb upstream and 1 kb downstream of the fragment inserted into a pcoDel plasmid. The PCR product was sequenced to confirm deletion of the fragment of the gene. To confirm that the selected gene was specifically targeted in the coDel mutant cells, mutants were rescued with a 3-kb PCR fragment encompassing the deleted sequences, and phenotypic analyses of the rescued cells were performed.

### Immunofluorescence and western blot analysis

The cells were fixed and stained on coverslips as previously described [30, 35]. The antibodies were used in the following final concentrations: monoclonal mouse anti-HA antibodies (Covance, Berkeley, CA) 1:300, polyclonal rabbit anti-HA antibodies (Cell Signaling Technology, Danvers, MA) 1:300, monoclonal rabbit anti-V5 antibodies (Cell Signaling Technology, Danvers, MA) 1:1600, polyclonal rabbit anti-GFP antibodies (Abcam, Cambridge, UK) 1:6000, anti-α-tubulin 12G10 antibodies (Developmental Studies Hybridoma Bank, Iowa University, Iowa City, IA, USA) 1:300, anti-mouse IgG Alexa-488 or anti-mouse IgG Alexa-555 antibodies (Invitrogen, Eugene, OR, USA) 1:300. After washing, the coverslips were mounted in Fluoromount-G (Southern Biotech, Birmingham, AL, USA) and viewed using a Zeiss LSM780 or a Leica TCS SP8 confocal microscope.

For western blots, cells were grown in SPP with shaking (80 rpm) at 30 °C and deciliated as previously described [45]. Cilia proteins (20 µg) were run on a 10% SDS-PAGE gel and transferred to a nitrocellulose membrane. After an hour of blocking with 5% non-fat milk in TBS, the membrane was incubated overnight at 4 °C with one of the following primary antibodies diluted in 1% non-fat milk in TBS: anti-HA (1:2000), anti-V5 (1:1600), or anti-GFP (1:60,000). After washing for 3 × 15 min in TBS-Tween 20, followed by a single wash in TBS, the membrane was incubated with secondary antibodies, either anti-mouse IgG–HRP or anti-rabbit IgG–HRP (Jackson ImmunoResearch, West Grove, PA), diluted 1:20,000 in 1% non-fat milk for 1.5 h at RT. After washing in TBS-Tween 20 (3 × 15 min) and TBS (15 min), proteins were detected using a Westar Supernova kit (Cyanagen, Italy).

### Mass spectrometry analyses

To identify proteins that co-immunoprecipitate with GFP-tagged Fap43p, the cytoskeletal fraction [46] or cilia from *Tetrahymena* cells overexpressing GFP–Fap43p or expressing Fap43p–GFP, respectively, and GFP-expressing cells (negative control) were harvested and processed as previously described [30]. To identify potential Fap43p or Fap44p-interacting proteins using proximity-dependent labeling, cells expressing either Fap43p–HA–BirA* or Fap44p–HA–BirA* were grown in SPP medium to a density of 2 × 10^5^ cells/ml, starved overnight in 10 mM Tris–HCl buffer, pH 7.5, and incubated in the same buffer supplied with 50 μM biotin for 2–4 h at 30 °C. After deciliation [45], the collected cilia were resuspended in 0.5 ml of axoneme stabilization buffer (20 mM potassium acetate, 5 mM MgSO_4_, 20 mM HEPES, 0.5 mM EDTA) and treated for 5 min on ice with 10 μl of 10% NP-40 to remove the ciliary membrane. After centrifugation (10 min at 21,100×*g* at 4 °C), the axonemes were resuspended in a lysis buffer (50 mM Tris–HCl, pH 7.4, 500 mM NaCl, 0.4% SDS, 1 mM DTT), incubated at RT for 1 h, and then centrifuged at 8000×*g* at 4 °C. The collected supernatant was diluted with an equal volume of 50 mM Tris–HCl buffer, pH 7.4, and incubated overnight with 100 μl of streptavidin-coupled Dynabeads (Dynabeads™ M-280 Streptavidin, Thermo Fisher Scientific, Waltham, MA, USA) at 4 °C. After washing (6 × 5 min with washing buffer: 15 mM Tris–HCl, pH 7.4 150 mM NaCl, 0.1% SDS, 0.3 mM DTT) at 4 °C, resin-bound proteins were analyzed on SDS-PAGE gel, followed by western blot with anti-streptavidin–HRP (Thermo Scientific, Rockford, IL, USA), diluted 1:40,000, or by mass spectrometry (Laboratory of Mass Spectrometry, Institute of Biochemistry and Biophysics, PAS, Warsaw, Poland).

For the entire cilia proteome, wild-type and mutant cells were deciliated as above, the cilia were collected and washed with 10 mM Tris, pH 7.4, and an equal amount of ciliary protein was run on SDS-PAGE gel and analyzed by mass spectrometry (Laboratory of Mass Spectrometry, Institute of Biochemistry and Biophysics, PAS, Warsaw, Poland).

### Phylogenetic analysis

Fap43p and Fap44p orthologs were identified in the NCBI protein database using Blastp search and either *Tetrahymena* or *Chlamydomonas* protein as bait. Protein amino acid sequences were aligned using the ClustalX2 program [47] and edited using the SeaView program [48]. The predicted protein sequence of *Chlamydomonas* Fap43p was manually corrected as described in the legend of Fig. S1. The domain analyses were performed using SMART (http://smart.embl-heidelberg.de/), WDSP (http://wu.scbb.pkusz.edu.cn/wdsp), [49–51] and COILS (http://www.ch.embnet.org) [52] programs.

## Results

### Knockout of *FAP43* reduces cilia beating amplitude

The bioinformatics screen of the human ciliome against *Tetrahymena thermophila* and *Arabidopsis thaliana* proteomes enabled the selection of numerous highly evolutionarily conserved and yet uncharacterized putative ciliary proteins. Among them, our attention was drawn to the *Tetrahymena* ortholog of *Chlamydomonas reinhardtii* Fap43p and the human protein Wdr96p (WD-repeat protein 96). The phylogenetic screen showed that Fap43p/Wdr96p orthologs are present in organisms that assemble motile cilia but not in the worm *C. elegans*, which forms only immotile sensory cilia (Fig. S1).

**Fig. 1:**
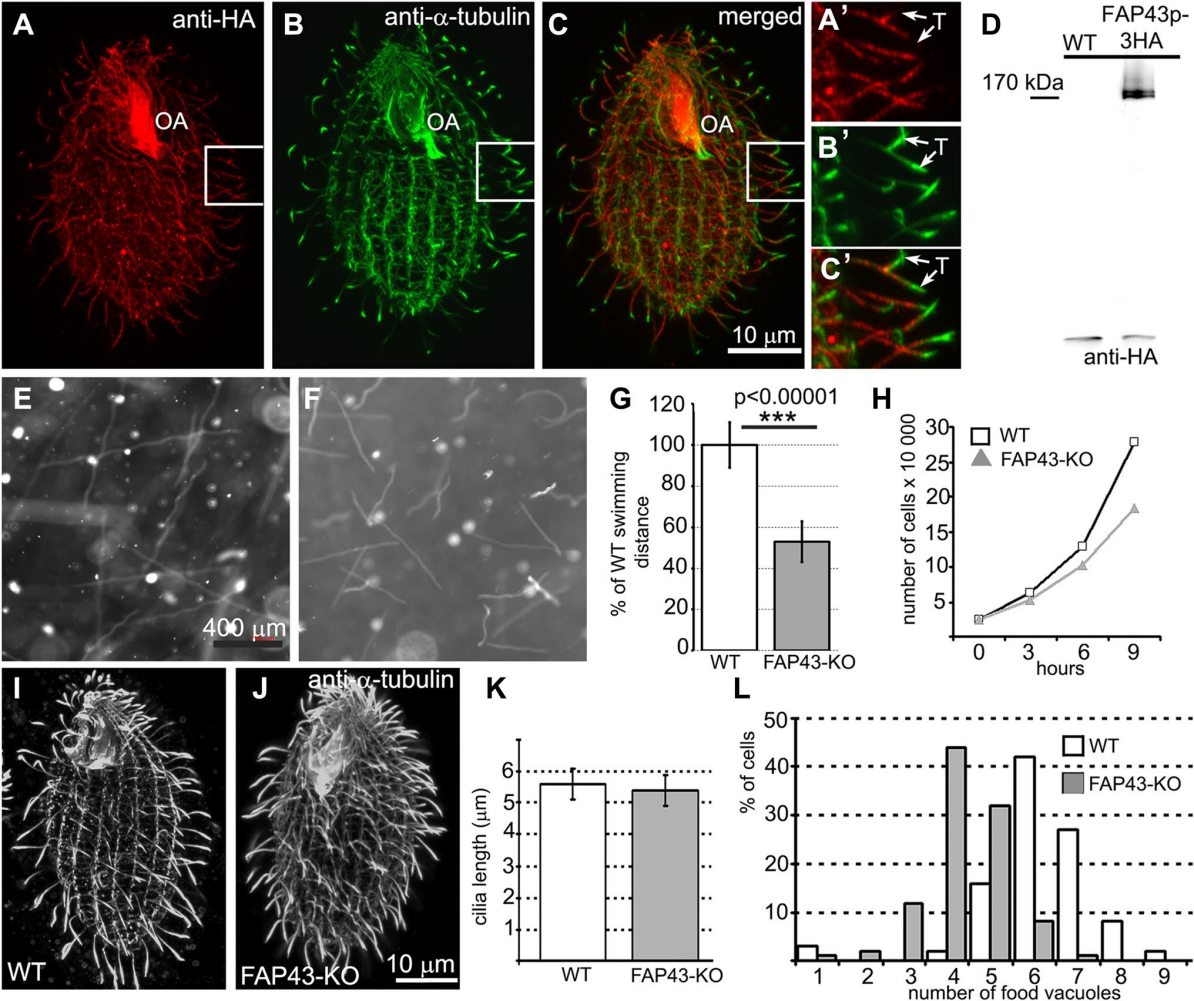
Fap43p–3HA localized throughout the cilia except at the cilia tips, and knockout of *FAP43* affects cilia function but not cilia length in *Tetrahymena* cells. **a**–**c′** Immunofluorescence confocal images of *Tetrahymena* cells expressing Fap43p–3HA at the native level, double labeled with anti-HA (**a**, **a′**) and anti-α-tubulin antibodies (**b**, **b′**). **c**, **c′** Merged images. **a′**, **b′**, **c′** The magnification of cilia marked with white insets in **a**, **b**, and **c**, respectively. *OA* oral apparatus, *T* cilium tip. Scale bar 10 μm. **d** Western blot analysis of the ciliary proteins isolated from wild-type and Fap43p–3HA-expressing cells. Note the presence of two HA-positive bands. Swimming paths of wild-type (**e**) and *FAP43* knockout cells (**f**) recorded for 3.2 s using a video camera. **g** Average distance swum during 3.2 s, normalized to the wild-type value (WT *n* = 139, *FAP43*-*KO n* = 146). Bars represent standard deviation, *p* value < 0.00001. **h** Growth curves of wild-type and *FAP43* knockout cells. Deletion of Fap43p does not affect cilia length. Immunofluorescence confocal images of wild-type (**i**) and *FAP43* knockout cells (**j**) stained with anti-α-tubulin antibodies. Scale bar 10 μm. (**k**) Graphical representation of wild-type (*n* = 130) and *FAP43* knockouts (*n* = 50) cilia length measurements. Bars represent standard deviation. **l** Graph showing the efficiency of the formation of food vacuoles based on the analyses of the number of India ink-filled food vacuoles per cell formed during 10 min. On average, wild-type and *FAP43*-*KO* cells formed 5.1 vacuoles (*n* = 406 cells) and 3.3 vacuoles (*n* = 344), respectively

*Tetrahymena* Fap43p is a 1678-amino acid protein with a calculated molecular weight of 198 kDa. Similar to other Fap43p orthologs, this protein contains two structurally distinct regions: a WD40-repeat-rich N-terminal region and a C-terminal region predicted to form coiled coils (Fig. S2a). When expressed under the control of the native promoter, Fap43p–3HA was exclusively targeted to cilia and was present along the entire length of the cilium, with the exception of the cilium tip (Fig. 1a–c′). Two closely co-migrating bands of Fap43p were detected in a western blot analysis of the ciliary proteins isolated from Fap43p–3HA-expressing cells (Fig. 1d). Because alternative splicing is infrequent in *Tetrahymena* cells [53, 54], it is most likely that Fap43p is posttranslationally modified.

To identify the region(s) of Fap43p required for its ciliary localization, we overexpressed either full-length Fap43p or the truncated variants of the protein (Fig. S2a–b) under the control of a cadmium-inducible *MTT1* promoter [55] from a non-essential *BTU1* locus. When overexpressed, full-length Fap43p localized to cilia, and the excess of the expressed protein accumulated near the basal bodies (Fig. S2c–c’). Similar localization was observed in cells that overexpressed the C-terminal fragment of Fap43p, which is predicted to form coiled-coils (Fap43p G667-Y1678, Fig. S2d). In contrast, the N-terminal fragment, containing only WD40 repeats (M1-K712), was most likely unstable or toxic, as it was maintained in the cytoplasm at a very low level (Fig. S2e). However, a fragment containing both the WD40 repeats and the subsequent three out of seven domains predicted to form coiled-coils, although prone to degradation (Fig. S2b), was detected at a low level in the cilia and cell cortex (Fig. S2f). Thus, the C-terminal fragment is indispensable and sufficient for ciliary localization of Fap43p.

**Fig. 2:**
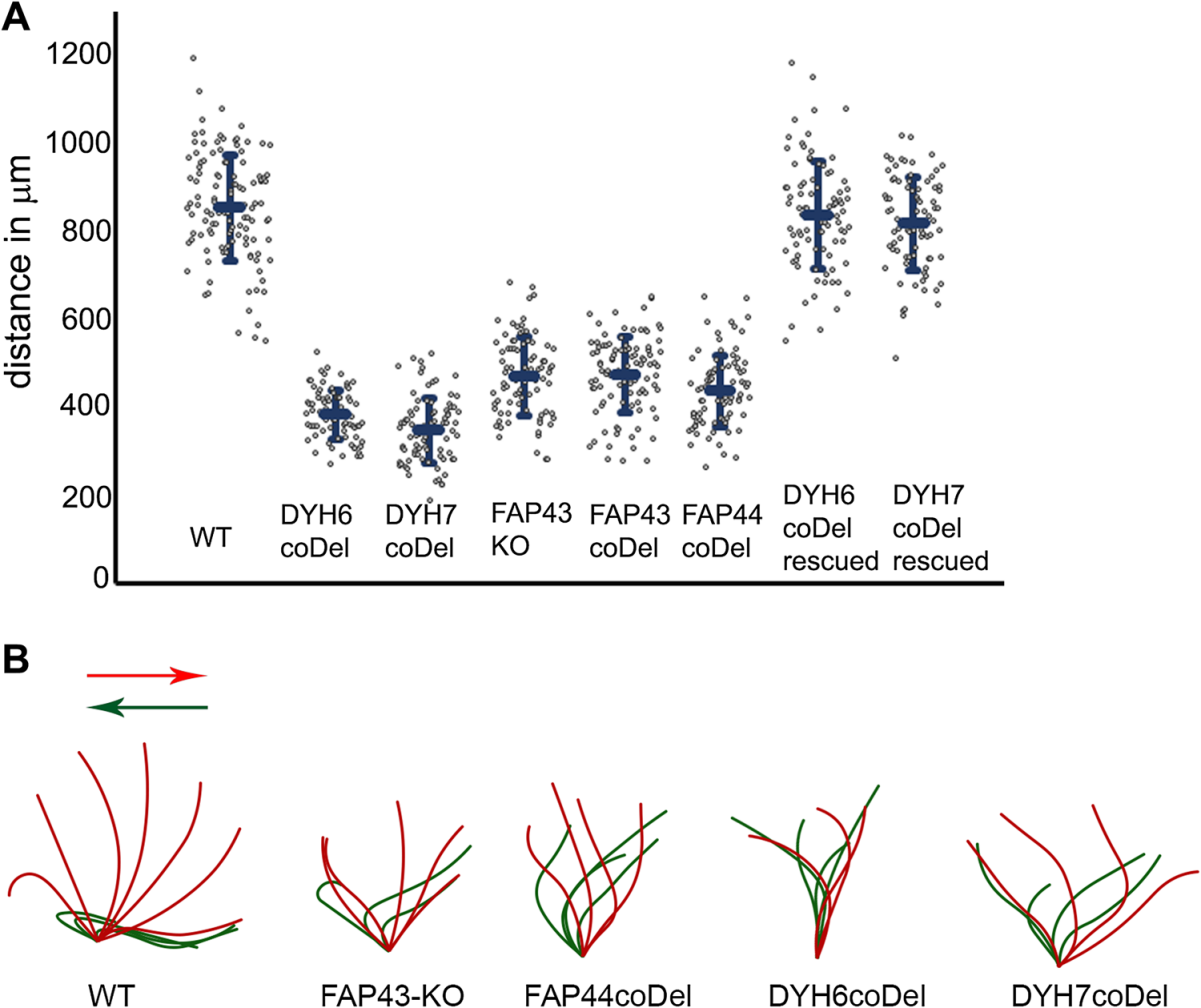
Comparison of the motility and ciliary waveform of wild-type and *Tetrahymena* mutants. **a** Graph representing distances with standard deviation swam during 3.2 s by wild-type (WT, 849 ± 120 μm, *n* = 117), *DYH6coDel* (381 ± 55 μm, *n* = 79), *DYH7coDel* (346 ± 73 μm, *n* = 89), *FAP43*-*KO* (469 ± 88 μm, *n* = 84), *FAP43coDel* (473 ± 84 μm, *n* = 100), *FAP44coDel* (435 ± 81 μm, *n* = 100), *DYH6coDel rescued* (833 ± 120 μm, *n* = 90), *DYH7coDel rescued* (814 ± 104 μm, *n* = 81). *N* number of measured paths. **b** Drawings representative of tracing of the power (red) and recovery (green) stroke of a single cilium: wild-type, *FAP43*-*KO*, *FAP44 coDel*, *DYH6 coDel* and *DYH7 coDel*. Drawings were prepared based on the images selected from high-speed videos (Fig. S5 and supplemental Movies)

To learn about the role of the protein, we generated *FAP43* germline knockout cells (*FAP43*-*KO*, Fig. S3). Compared to wild-type cells, *Tetrahymena* mutants lacking Fap43p exhibited a phenotype typical of cells with ciliary defects, including reduced cell swimming speed, proliferation rate and phagocytosis rate. The video recording of live cells showed that *FAP43*-*KO* cells swam with significantly reduced velocity and on average traveled half the distance (53 ± 10%) traveled by wild-type cells (Figs. 1e–g, 2a, Fig. S4, Supplemental Movies 1 and 2). In the ciliate *Tetrahymena*, cilia beating contributes to the separation of two daughter cells during the final stage of cell division [56]. Accordingly, cells lacking Fap43p multiplied more slowly than wild-type cells (Fig. 1h). The calculated doubling time of the *FAP43* knockouts was approximately an hour longer than that of wild-type cells (wild-type—2.4 h, *FAP43*-*KO*—3.5 h). Near the cell anterior, *Tetrahymena* cells form an oral apparatus, a funnel-like structure surrounded by four rows of ciliated basal bodies. During phagocytosis, funneling of the food particles to the oral cavity depends upon the synchronous beating of oral cilia [57]. Cells lacking Fap43p formed fewer food vacuoles than did wild-type cells, as was visualized by growing cells in a medium supplemented with India ink (Fig. 1l).

Immunofluorescence light microscopy with anti-α-tubulin antibodies revealed that *FAP43*-*KO* cells assemble cilia in similar number and length as wild-type cells (Fig. 1i–k). Therefore, the reduced efficiency of cilia-related processes was most likely caused by impaired ciliary motility. Therefore, we used a high-speed camera to record cilia beating in wild-type and mutant cells. In the wild-type cells, cilia beat synchronously, with well-defined phases of the power and recovery strokes that take place in different beating planes (Fig. 2b, Fig. S5, Supplemental Movie 3). During the power stroke phase, cilia are stiff and stroke in the direction of the cell posterior end, marking a semicircle perpendicular to the cell surface. During the recovery phase, cilia bend and move parallel to the cell surface, returning to the initial position with their tips facing the cell anterior end. In *FAP43* knockouts, the waveform of the cilia beat was altered; the extent of the strokes was approximately half that of the control, and the cilia were more perpendicular to the cell surface at the beginning and end of each beat cycle (Fig. 2b, Fig. S5, Supplemental Movie 4). Although some cilia started the power stroke and finished the recovery stroke closer to the cell surface, the beating amplitude remained smaller than that in wild-type cells.

### Fap43p interacts with Fap44p, and their localization to cilia is interdependent

Because classical transmission electron microscopy analysis did not reveal any apparent changes in the ultrastructure of the axoneme of *FAP43* knockouts (data not shown), to address the question of Fap43p localization and function in cilia, we searched for Fap43p-interacting proteins using two biochemical approaches. First, we immunoprecipitated GFP-tagged Fap43p from either ciliary or cytoskeletal fractions isolated from cells expressing Fap43p–GFP at the native level or overexpressing GFP–Fap43p, respectively, using anti-GFP antibodies and analyzed the immunoprecipitates by mass spectrometry. The corresponding fractions isolated from cells expressing the GFP protein alone were used as controls (Fig. S6a–b). The comparison of the experimental and control immunoprecipitates indicated that Fap43p and an ortholog of *Chlamydomonas* Fap44p were absent in the controls but predominant among the proteins identified in immunoprecipitates from cells expressing GFP-tagged Fap43p (Table 1, Tables S2, S3).

**Table 1:** Mass spectrometry analysis of proteins co-immunoprecipitated with GFP-tagged Fap43p or biotinylated in cells expressing either Fap43–HA–BirA* or Fap44–HA–BirA*

Protein	Total number of peptides				
	Number in TGD	GFP–Fap43p–oex	Fap43p–GFP native	Fap43p–BirA* native	Fap44p–BirA* native
Fap43	TTHERM_00196190	682/454	12/7	74/52	32/18
Fap44	TTHERM_00498220	215/188	13/9	57/37	31/18
Fap57A	TTHERM_00105300	0	0	31/19	56/30
Fap57B	TTHERM_00052490	0	0	6/4	3/2
Fap57C	TTHERM_00214710	0	0	3/3	4/3
Dyh6	TTHERM_00688470	0	0	1/1	2/2
Dyh7	TTHERM_00912290	0	0	0	0
Dyh3	TTHERM_01276420	10/10	0	1/1	0/0
Dyh4	TTHERM_00499300	11/11	0	2/2	2/2
Dyh5	TTHERM_00486600	15/15	0	0/0	0/0

In the corresponding control samples, the proteins listed in the table were not identified. Note that the number of identified peptides of Fap43p and Fap44p is significantly larger than the number of peptides of other proteins. In *Tetrahymena* cells, the excess of the overexpressed GFP–Fap43p accumulates near the basal bodies (Fig. S2). Co-precipitation of ODA dynein heavy chains in cells overexpressing GFP–Fap43p is likely due to their accumulation at the cilia base before transport to the cilia (in the case of overexpressed GFP–Fap43p, the cytoskeletal fraction was analyzed). Note that only 1–2 peptides of Dyh3p and Dyh4p were detected in samples prepared from cells expressing either Fap43p or Fap44p at the native level (similar to the number of the Dyh6 peptides; however, in contrast to IDA I1, there are four ODAs in each axonemal unit). All proteins identified in the experimental and control samples are listed in Tables S2–S5

When overexpressed, GFP–Fap43p immunoprecipitated not only with Fap44p but also with three nucleoporins (Nup93, Nup155 and Nup308), kinesin type II and dynein heavy chains of the outer dynein arms (Table S2). However, the number of Fap43p- and Fap44p-specific peptides was significantly higher than the number of peptides of the other co-precipitated proteins (Table S2). Because the majority of the overexpressed GFP–Fap43p accumulates near the basal bodies (Fig. S2c), and because nucleoporins and outer arm dyneins were not identified as potential Fap43p partners when Fap43p was expressed at the native level and purified from cilia (Table 1, Tables S3, S4), we suspect that these proteins are not true Fap43p interactors.

Because of the limitations of the co-IP approach, we next took advantage of the BirA* proximity labeling assay, which enables the identification of potential interactors that are within 10 nm of the target [58]. We isolated cilia from either wild-type *Tetrahymena* cells or cells expressing Fap43p–HA–BirA* under the control of a *FAP43* promoter, both grown for 4 h in a medium supplied with 0.05 mM biotin. The biotinylated proteins (Fig. S6c) were purified using streptavidin-coated beads and analyzed by mass spectrometry. In addition to Fap43p and Fap44p, we repeatedly identified three orthologs of *Chlamydomonas* Fap57p, with one ortholog, Fap57Ap, predominating (Table 1, Table S4).

The reciprocal experiment with cilia isolated from cells expressing C-terminally BirA*-tagged Fap44p (Fig. S6d) strengthens the hypothesis that Fap43p, Fap44p and Fap57p are positioned in close proximity in the *Tetrahymena* axoneme (Table 1, Table S5). Interestingly, the ratio of the Fap57p peptides to either the Fap43p or Fap44p peptides was higher if Fap44p was tagged with BirA* ligase (Table 1). Thus, it is very likely that the C-terminal end of Fap44p is positioned closer to Fap57p than is the C-terminal end of Fap43p. A few peptides of Dyh6p, the dynein heavy chain of the two-headed inner dynein arm IDA I1, were also identified in Fap43p–HA–BirA* and Fap44p–HA–BirA* precipitates (Table 1).

**Fig. 3:**
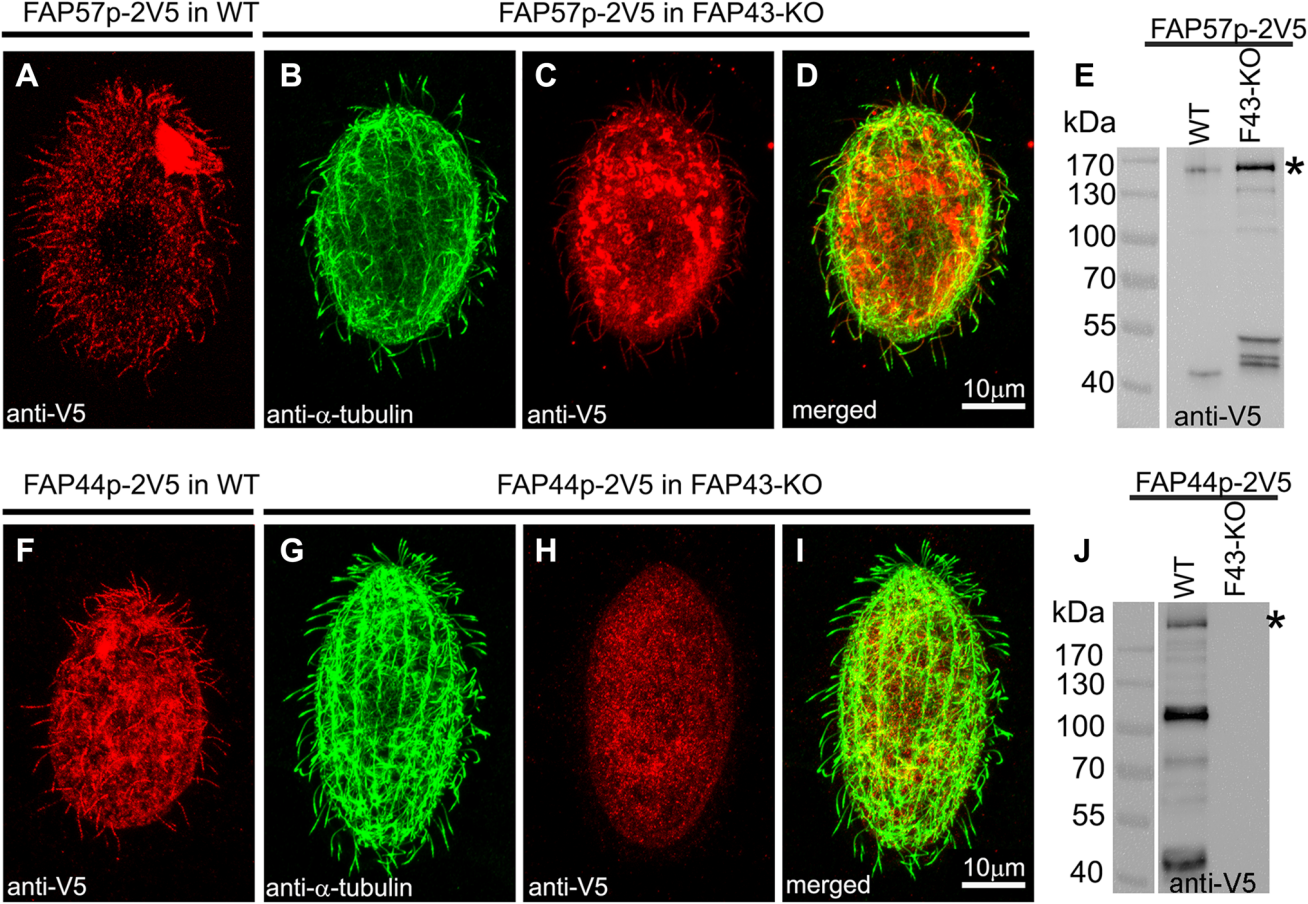
Fap43p is indispensable for Fap44p but not Fap57p ciliary localization. **a**–**d**, **f**–**i** Immunofluorescence confocal images of either wild-type (**a**, **f**) or *FAP43*-*KO Tetrahymena* cells (**b**–**d**, **g**–**i**) expressing either Fap57p–2V5 (**a**–**d**) or FAP44p–2V5 (**f**–**i**) under the control of their native promoters, double labeled with anti-V5 (**a**, **c**, **f**, **h**) and anti-α-tubulin antibodies (**b**, **g**). **d**, **i** Merged images. Western blot analysis of ciliary proteins isolated from wild-type and *FAP43*-*KO* cells expressing either Fap57p–2V5 (**e**) or Fap44p–2V5 (**j**)

**Fig. 4:**
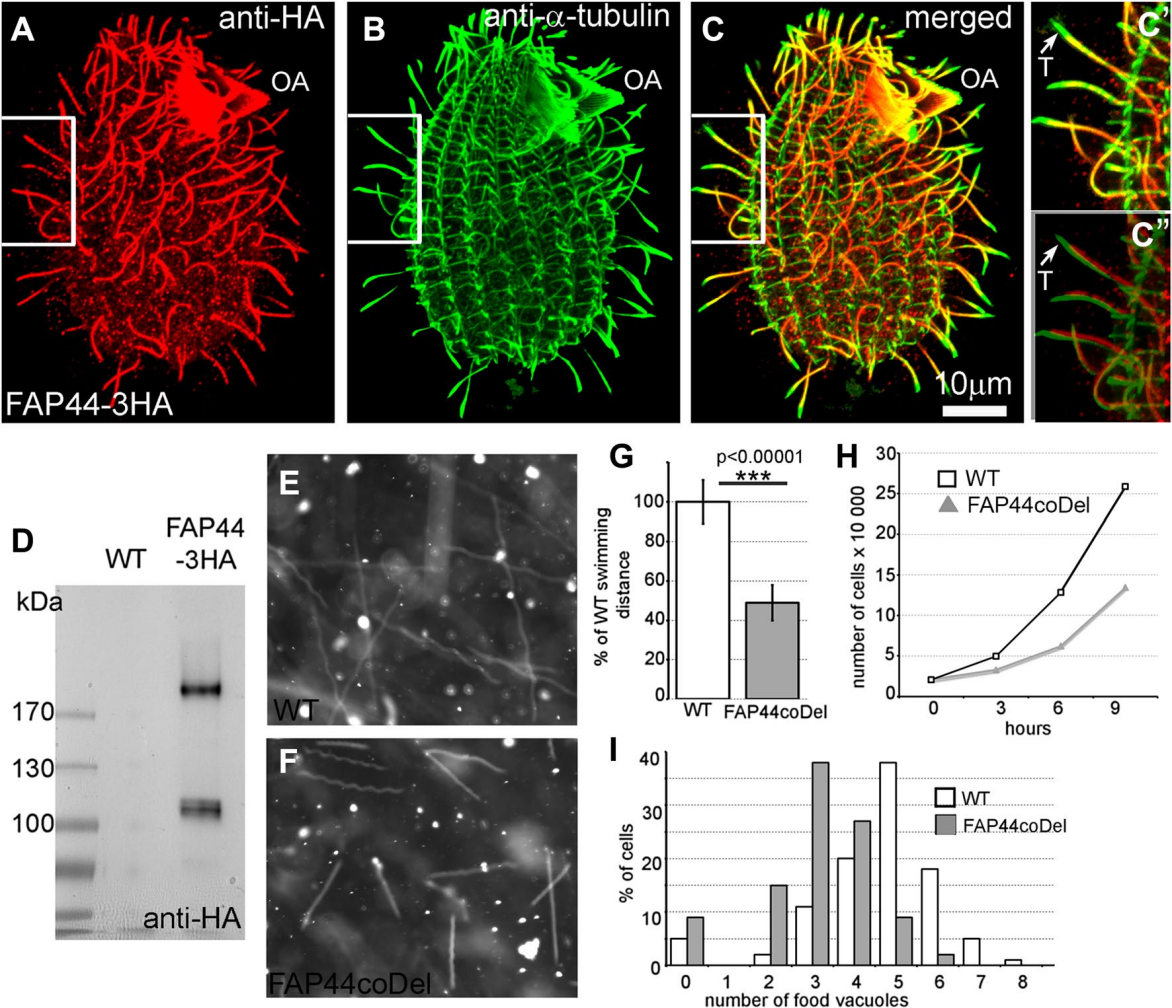
Fap44p–3HA, similar to Fap43p localized throughout the cilia, except the cilia tips, and is indispensable for proper cilia function. **a**–**c″** Immunofluorescence confocal images of *Tetrahymena* cells expressing Fap44p–3HA at the native level, double labeled with anti-HA (**a**) and anti-α-tubulin antibodies (**b**). **c**–**c″** Merged images. **c′**–**c″** Magnifications of cilia marked with white insets in **a**–**c**. **c″** Note a shift in the merging of the **a** and **b** magnified images enabling better visualization of the cilia tips lacking Fap44p. *OA* oral apparatus, *T* cilium tip. Scale bar 10 μm. **d** Western blot analysis of ciliary proteins isolated from wild-type and Fap44p–3HA-expressing cells stained with anti-HA antibodies. Swimming paths of wild-type (**e**) and FAP44coDel mutants (**f**) recorded for 3.2 s using a video camera. **g** Average distance swum during 3.2 s, normalized to the wild-type value (WT, *n* = 139; FAP44coDel, *n* = 146). Bars represent standard deviation, *p* value < 0.00001. **h** Growth curves of wild-type and FAP44coDel mutant cells. **i** Graph showing the efficiency of the formation of food vacuoles based on the analyses of the number of India ink-filled food vacuoles per cell formed during 10 min. On average, wild-type and FAP44coDel cells formed 4.6 vacuoles (*n* = 100 cells) and 3.1 vacuoles (*n* = 100 cells), respectively

Fap44p and Fap57Ap, the orthologs of human Wdr52 and Wdr65, respectively, are large, previously uncharacterized ciliary proteins with calculated molecular weights of 215 and 152 kDa, respectively (Figs. 3a, e, f, j, 4a–d). Both proteins are evolutionarily conserved in organisms assembling motile cilia or flagella (Figs. S7, S8). Their N-terminal fragments are enriched in WD repeats, while the C-terminal regions contain coiled-coil domains (Figs. S9a, e). Thus, the domain compositions of Fap44p and Fap57p are similar to that of Fap43p. Moreover, similar to Fap43p, the HA-tagged C-terminal fragment of Fap44p containing coiled-coil domains was necessary and sufficient for its ciliary localization, while the WD-repeat-rich N-terminal fragment accumulated in the cell body (Fig. S9b–d).

Next, we analyzed whether the ciliary localization of Fap44p and Fap57p depends upon the presence of Fap43p. We prepared constructs enabling the expression of 2xV5-tagged Fap44p or Fap57p under the control of the respective native promoters and used these to transform either wild-type (control) or mutant cells lacking Fap43p. The immunofluorescence analysis showed that Fap57p–2V5 expressed at the native level was targeted to cilia in both wild-type and *FAP43*-*KO* cells (Fig. 3a–e). Thus, the cilia targeting or docking of Fap57p does not depend upon the presence of Fap43p. In contrast to Fap57p, Fap44p–2V5 was not detected in cilia of *FAP43* knockouts. Instead, this protein accumulated within the cell body (Fig. 3g–j). On the other hand, Fap44p–2V5, expressed in wild-type cells, was successfully targeted to cilia (Fig. 3f). Thus, Fap43p seems to be required for the targeting or docking of Fap44p to cilia. These observations were confirmed by mass spectrometry data, which showed that both Fap43p and Fap44p are absent from cilia purified from *FAP43*-*KO* cells (Table 2, Table S6).

**Table 2:** Comparative proteomics of ciliary proteins in wild-type and *FAP43* and *FAP44* mutant cells

Proteins	Number of unique peptides			
	WT	FAP43-KO	FAP43coDel	FAP44coDel
Atu1 α-tubulin	2739/53	1924/52	2890/53	2646/50
Btu1 β-tubulin	3058/56	2423/56	2949/52	3072/51
ODA Dyh3	1616/425	1055/426	1411/440	877/261
ODA Dyh4	1702/448	1127/424	1395/445	981/284
ODA Dyh5	1164/345	906/355	990/355	701/225
IDA I1a Dyh6	440/234	371/236	377/222	98/59
IDA I1b Dyh7	387/220	356/252	359/218	86/61
Fap43	100/69	0	0	0
Fap44	88/64	0	0	0
Fap57A	43/36	43/36	40/35	21/20
Fap57B	19/17	29/26	15/14	9/9
Fap57C	19/18	12/12	8/8	5/5

Mass spectrometry analysis of the axonemal proteins from *Tetrahymena* wild-type, *FAP43* knockout (*FAP43-KO* and *FAP43coDel*) and *FAP44coDel* mutant cells. The table shows the numbers (X/Y) of all the identified peptides (X) and identified unique peptides (Y). Note that Fap43p and Fap44p are missing in mutant but not in wild-type axonemes (all identified proteins are listed in Table S6)

### Lack of either Fap43p or Fap44p affects cilia beating in a similar way

To further analyze the interdependence between Fap43p and Fap44p, we obtained somatic knockouts of *FAP44* (Fig. S10) by targeted gene disruption in macronuclei (co-deletion method, coDel) [43]. The phenotypic analysis showed that, similar to *FAP43*-*KO* cells, *FAP44*-*KO* cells exhibited alteration of cilia-dependent processes. *Tetrahymena* cells lacking Fap44p swam (Figs. 2a, 4e–g, Fig. S4, Supplemental Movie 5), proliferated (Fig. 4h) and formed vacuoles (Fig. 4i) at a lower rate than the wild-type cells. The waveform and amplitude of the beating cilia of *FAP44*-*KO* mutants were similar to those observed in *FAP43*-*KO* mutants, but we observed either immotile or rotating cilia even more frequently (Fig. 2b, Fig. S5, Supplemental Movie 6).

**Fig. 5:**
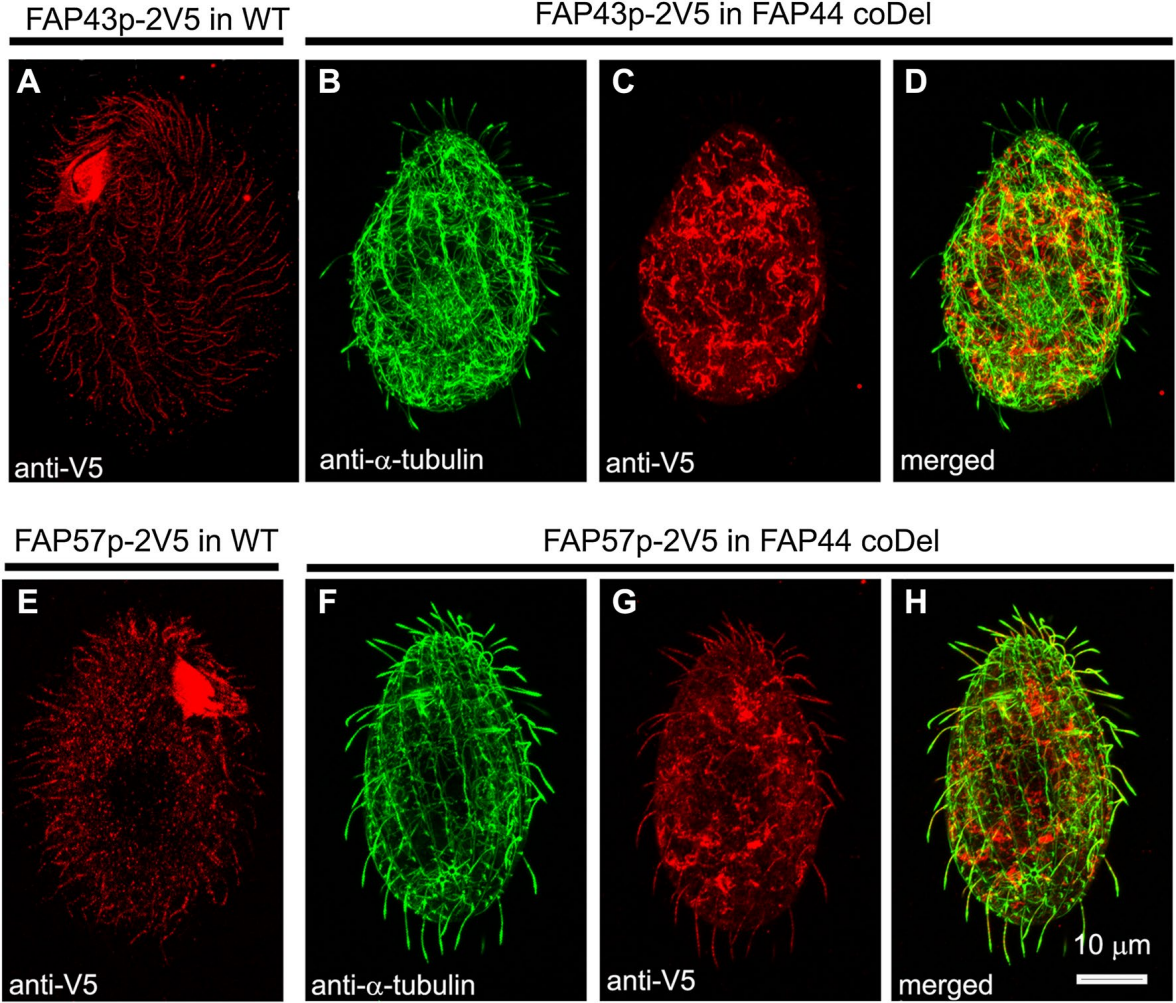
Fap44p is indispensable for Fap43p but not Fap57p ciliary localization. Immunofluorescence confocal images of either wild-type (**a**, **e**) or *FAP44 coDel Tetrahymena* cells (**b**–**d**, **f**–**h**) expressing either Fap43p–2V5 (**a**–**d**) or FAP57–2V5 (**e**–**h**) under the control of their native promoters, labeled with anti-V5 (**a**, **c**, **e**, **g**) and anti-α-tubulin antibodies (**b**, **f**). **d**, **h** Merged images

To investigate whether Fap44p is required for Fap43p ciliary localization, we introduced a transgene enabling the expression of Fap43p–2V5 under a *FAP43* promoter into wild-type and *FAP44* knockout cells. Whereas in the wild-type cells, the Fap43p–2V5 fusion protein was detected in cilia (Fig. 5a), in cells with deleted Fap44p, the cilia lacked Fap43p–2V5 (Fig. 5b–d). Thus, in *Tetrahymena*, Fap44p is required for Fap43p ciliary localization. Accordingly, Fap43p was absent in cilia purified from *Tetrahymena FAP44* knockout mutants, as revealed by mass spectrometry analysis (Table 2, Table S6).

Similar localization experiments for Fap57p showed that Fap57Ap–2V5 was effectively targeted to cilia in wild-type and *FAP44*-*KO* coDel cells (Fig. 5e–h). Thus, it is most likely that Fap43p and Fap44p are subunits of the same ciliary complex, and their presence in cilia is interdependent, but neither of the proteins is required for ciliary localization of Fap57p.

### Ciliary beating is similarly affected in Dyh6p, Dyh7p, Fap43p and Fap44p mutants

It is thought that the size and waveform of the ciliary bend are controlled by the inner dynein arms [4, 7]. Some Dyh6p peptides were identified in the precipitates of biotinylated proteins from cilia of Fap43p–HA–BirA* or Fap44p–HA–BirA* expressing cells (Table 1, Tables S4, S5; in *Tetrahymena*, Dyh6 and Dyh7 are the dynein heavy chains of the two-headed inner dynein arm I1 (dynein f) [59–61]). Thus, it is plausible that the Fap43p/Fap44p-containing complex is positioned near the IDA I1 motor domains. Using the coDel approach, we engineered *Tetrahymena* knockout cells with a disruption of the *DYH6* or *DYH7* gene (Fig. S10). To ensure the specificity of the gene targeting, we rescued *DYH6coDel* and *DYH7coDel* mutants with approximately 3 kb PCR fragments containing part of the 5′UTR and ORF of *DYH6* or *DYH7*, respectively (rescue experiment, Fig. S4e–h).

Next, we compared the swimming speed and cilia beating pattern of *DYH6* and *DYH7* knockout mutants with those of the *FAP43* or *FAP44* knockouts (Fig. 2, Fig. S4). Mutants lacking either Dyh6p or Dyh7p swam slowly and on average traveled 43 ± 6 and 39 ± 8%, respectively, of the distance swum at the same time by wild-type cells, i.e., approximately 10% less than the distance traveled by *FAP43*-*KO* and *FAP44*-*KO* mutants (Fig. 2a, Fig. S4, Supplemental Movie 7, 8). *DYH6coDel* and *DYH7coDel* rescued cells swam at a similar speed as the wild-type cells (Fig. 2a, Supplementary Movie 9, 10), strongly suggesting that we specifically deleted a fragment of the *DYH6* or *DYH7* gene, respectively.

The analysis of the ciliary waveform revealed that both the power and recovery strokes were incomplete in the *DYH6* and *DYH7* knockout mutants (Fig. 2b). However, we noticed that, similar to the case in *FAP43*-*KO* and *FAP44*-*KO* mutants, not all cilia beat in an identical way, with some being immotile or rotating, especially in *DYH7* knockout mutants (Supplemental Movies 11, 12). Thus, the lack of IDA I1 dynein heavy chains affects cell motility more severely than the deletion of *FAP43* or *FAP44*. Moreover, inner arm I1 dyneins are present in *FAP43* and *FAP44* mutant ciliary proteomes (Table 2, Table S6). Thus, we assume that the Fap43p–Fap44p complex is not required for IDA I1 docking but perhaps could regulate its activity.

Fap43p and Fap44p are essential for proper ciliary motility and function also in the unicellular green algae *Chlamydomonas*. *Chlamydomonas* mutant cells lacking either Fap43p (*fap43*) or Fap44p (*fap44*) (Fig. S11a, b) swam at a reduced speed compared to wild-type cells (Fig. S11d, e) and showed some alteration in flagella behavior (Supplemental Movies 13–15). In contrast to *Tetrahymena*, in *Chlamydomonas* Fap244p (identified only in the proteomes of *Chlamydomonas* and *Volvox*) co-assembles with the Fap43p/Fap44p complex [62]. Mutant cells lacking Fap244p (*fap244*) swam with only a slightly reduced rate compared to wild-type cells (Figs. S11c, e). Thus, Fap43p and Fap44p perform an important function in both cilia and flagella, and their role is likely evolutionarily conserved.

## Discussion

Genetic, proteomic and microscopic analyses conducted in the last few years have delivered a significant amount of new data that have expanded our knowledge about motile cilia structure and beating regulation. At the same time, we are only beginning to understand the complexity of the molecular mechanisms that regulate the function of these highly sophisticated cellular nanomachines. One of the obstacles to fully deciphering the molecular mechanisms that regulate cilia beating in time and space is insufficient knowledge about the ciliary proteome, as well as about the functions of and interactions among the proteins that build cilia. It is estimated that cilia are composed of hundreds of proteins [20, 21]. However, numerous proteins of the cilia proteome are only hypothetical ciliary proteins, and their precise localization in cilia and their roles are unknown. At the same time, cryo-electron tomography analyses and 3D reconstruction of the 96-nm axonemal unit revealed the existence of as-yet uncharacterized minor complexes of unknown protein composition and function. It is likely that these minor complexes function in signal transduction from the central pair complex to the dynein motors or regulate the functions of the major ciliary complexes.

Searching for “missing links” in the chains of interacting proteins that are involved in cilia beating regulation, we focused our attention on the orthologs of two highly evolutionarily conserved ciliary proteins, Fap43p and Fap44p. We showed that both proteins likely form a minor ciliary complex and that they are essential for proper ciliary and flagellar beating. The biochemical analyses suggested that the Fap43p/Fap44p complex may be located in close proximity to IDA I1. Dyh6p, the 1-alpha DHC of IDA I1, was identified among the biotinylated proteins in cells expressing either Fap43p–BirA* or Fap44p–BirA*. However, the number of Dyh6p-specific peptides was low, suggesting that the distance between IDA I1 and the C-terminal end of Fap43p and Fap44p could be approximately 10 nm or slightly more, or that this distance could vary and that the motor domain of IDA I1 is only temporarily in the range of the BirA* ligase. Interestingly, cryo-electron tomography of axonemes isolated from wild-type *Chlamydomonas* revealed that one of the minor complexes of yet undetermined protein composition is positioned proximally to IDA I1 [13]. This so-called tether/tether head complex seems to connect the IDA I1α motor domain to the A-tubule of the outer doublet and was suggested to function as a sensor of the distance between the IDA I1α motor domain and the microtubule surface [13]. It is possible that Fap43p/Fap44p are subunits of the tether/tether head complex. This assumption is supported by data obtained during the cryo-electron tomography analyses of the axonemes isolated from *Tetrahymena FAP43* knockout cells showing that tether/tether head complex is missing in this mutant [62].

The mass spectrometry analyses of the entire cilia proteome indicated that Dyh6p and Dyh7p, which build the IDA I1α and IDA I1β motor domains, respectively [59–61], are present in *FAP43* and *FAP44* knockout mutants. Thus, based on the data presented here, we hypothesize that the Fap43p/Fap44p complex is not required for IDA I1 assembly or docking to the axoneme. This conclusion is also supported by cryo-ET data of axonemes isolated from the *FAP43*-*KO* mutant, which showed that the IDA I1 complex is present in the majority of the axonemal units [62]. We speculate that the Fap43p/Fap44p complex could interact with IDA I1 and affect its motor activity. Such interactions could be regulated by, for example, the posttranslational modification of Fap43p. We detected two co-migrating bands of Fap43p–3HA on a western blot, which suggests either an alternative splicing of *FAP43* or posttranslational modification of Fap43p. Because alternative splicing is infrequent in *Tetrahymena* [53, 54], the posttranslational modification of Fap43p is more plausible; such modifications could affect the properties of Fap43p and subsequently its interactions with IDA I1.

Based on the biochemical analyses, both Fap43p and Fap44p are positioned near the evolutionarily conserved yet uncharacterized protein Fap57. The *Tetrahymena* genome encodes at least four proteins with homology to *Chlamydomonas* Fap57, but only one of the *Tetrahymena* orthologs, Fap57A, predominates among ciliary proteins biotinylated in cells expressing either Fap43–HA–BirA* or Fap44–HA–BirA*. Because cryo-electron tomography analyses revealed that the entire tether/tether head complex is missing in *Tetrahymena FAP43*-*KO* cells [62] and Fap57Ap is present in cilia even in the absence of either Fap43p or Fap44p, it is likely that Fap57Ap, unlike Fap43p and Fap44p, is not a subunit of the tether/tether head complex but could form a linker between Fap43p/Fap44p complex and other axonemal macrocomplexes.

The overexpressed GFP–Fap43p not only localized to cilia but also accumulated near the basal bodies and co-immunoprecipitated with nucleoporins (Nup93, Nup155 and Nup308), kinesin type II and the dynein heavy chains of the outer dynein arms (Table S2). In mammalian cells, nucleoporins were detected at the cilium base [63, 64]. Additionally, preassembled ODA and kinesin II-type motor protein accumulate near the ciliary base to be transported into the cilium. Because neither nucleoporins nor outer arm dyneins were identified as potential Fap43p partners when Fap43p was expressed at the native level and purified from cilia (Table 1, Tables S3, S4), we concluded that these proteins are not true Fap43p interactors.

Both Fap43p and Fap44p share similar domain organization; the N-terminal half is enriched in WD repeats, while the C-terminal half is predicted to form several coiled-coils. When overexpressed as truncated fragments, only the C-terminal fragment localized to cilia and near the basal bodies, whereas the N-terminal WD40-repeat-containing fragments accumulated in the cell body and were most likely degraded. Thus, the C-terminal fragment of both Fap43p and Fap44p is required and sufficient for ciliary transport and localization.

Most axonemal proteins are delivered to cilia as cargo by intraflagellar transport (IFT) particles. However, with a few exceptions, it remains unknown which IFT subunit(s) mediates the transport of axonemal proteins and whether the cargo binds directly to the IFT subunit or requires an adaptor protein. Previously, it has been suggested that coiled-coils mediate the interactions between IFT subunits [65], but to our knowledge, there are no data showing interactions between IFT subunits and cargo involving coiled-coils. It is also possible that only a fragment of the C-terminal part of Fap43p or Fap44p mediates IFT subunit or adaptor protein recognition and binding. Further analyses are required to uncover the molecular mechanism underlying Fap43p and Fap44p ciliary transport.

Recent whole-exome sequencing of men with MMAF (multiple morphological abnormalities of the flagella) revealed that biallelic mutations in *CFAP43* and *CFAP44* result in diverse morphological sperm defects and male infertility [66]. It is plausible that mutations in Fap43p or Fap44p could also affect the function of motile cilia assembled in different organs and not only in sperm cells. In humans, coordinated ciliary beating drives, e.g., cleaning of the airways, circulation of the cerebrospinal fluid in the brain ventricles and transport of oocytes in the Fallopian tubes prior to fertilization. The movement of the nodal cilia ensures proper left–right asymmetry of the internal organs in the human body. Defects in the assembly or function of the motile cilia cause a multisymptomatic, genetically heterogeneous disorder called primary ciliary dyskinesia (PCD), which affects one per 15,000–30,000 individuals [67–70]. Interestingly, the genetic background of many recognized cases of PCD remains unknown. Since Fap43p and Fap44p are indispensable for proper cilia beating in *Tetrahymena* and both proteins are highly evolutionarily conserved, it is plausible that mutations in either *FAP43* or *FAP44* may cause PCD. Thus, Fap43p and Fap44p could be new candidate protein markers for PCD or other ciliopathies.

## Electronic supplementary material

Below is the link to the electronic supplementary material.

**Fig. S1:** Multiple alignment of Fap43p homolog sequences: *Anopheles gambiae* (Ag, XP_312857.5), *Chlamydomonas reinhardtii* (Cr, XP_001698838.1), *Ciona intestinalis* (Ci, XP_018671542.1), *Danio rerio* (Dr, XP_697139.4), *Diachasma alloeum* (Da, XP_015109804.1), *Gallus gallus* (Gg, XP_015144200.1), *Homo sapiens* (Hs, XP_005270228.1), *Leishmania braziliensis* (Lb, XP_001567348.2), *Paramecium tetraurelia* (Pt, XP_001445436.1), *Pseudocohnilembus persalinus* (Pp, KRX02904.1), *Sinocyclocheilus rhinocerous* (Sr, XP_016390257.1), *Tetrahymena thermophila* (Tt, XP_001017273.3), *Trypanosoma brucei gambiense* (Tb, XP_011773146.1), *Trypanosoma vivax* (Tviv, CCD18628.1), *Volvox carteri f. nagariensis* (Vc, XP_002952887.1), *Xenopus tropicalis* (Xt, XP_017947697.1). The predicted *Chlamydomonas* protein sequence was corrected based on an analysis of the whole-genome shotgun sequence (https://phytozome.jgi.doe.gov, Cre16.g691440 | chromosome_16) and led to the identification of a fragment with high homology to the *Volvox* Fap43 protein. (PDF 112 kb)

**Fig. S2**: A C-terminal coiled-coil-domain-containing fragment of Fap43p is sufficient for targeting to cilia. (A) Schematic representation of the motifs and domains identified in full-length Fap43p and its truncated versions. Red rectangles represent WD40 repeats as predicted by the SMART and WDSP programs; blue rectangles represent coiled-coil domains as predicted by the SMART and COILS programs. (B) Western blot analysis of the cytoskeletal proteins isolated from cells overexpressing either GFP-tagged full-length Fap43p or HA-tagged truncated versions. The numbers represent the molecular weight size marker and refer to all blots. The stars mark the positions of the detected bands corresponding to the predicted molecular mass of the overexpressed proteins (GFP-Fap43p M1-Y1678 = 224 kDa, Fap43p-HA M1-D1353 = 159 kDa, Fap43p-HA G667-Y1678 = 121 kDa). The majority of the Fap43p-HA M1-D1353 fragment is degraded (although prepared under the same conditions as the other samples). Fap43p-HA M1-K712 was undetectable on the western blot. (C-F) Immunofluorescence confocal images of cells overexpressing either full-length GFP-tagged Fap43p (C, C’) or truncated versions of the protein (D-F) containing coiled-coil domains (D), WD40 repeats (E) or WD40 repeats and 3 out of 7 coiled-coils (F). Note that the Fap43p M1-K712 fragment is hardly detectable. C’ – overexposed cell presented in image C to visualize GFP-Fap43p in cilia. (TIFF 1511 kb)

**Fig. S3**: PCR analysis of the *FAP43* locus in wild-type and *FAP43* knockout cells. (A) A schematic representation of the *FAP43* locus in wild-type and *FAP43* knockout cells. The white rectangle represents a fragment of the *FAP43* ORF replaced by the neo4 cassette. Annealing (if it occurs) of the primers to the *FAP43* locus is indicated by arrows. (B) PCR analysis of the *FAP43* locus with primers indicated in scheme (A) demonstrates that a fragment of the ORF was removed from the *FAP43* locus. Amplification of the *FAP208* locus was used as a control for the quality of the isolated genomic DNA. (TIFF 315 kb)

**Fig. S4**: Comparison of the motility of wild-type *Tetrahymena* and studied mutants. (A-H) Swimming paths of (A) wild-type, (B) *FAP43*-*KO*, (C) *FAP43*-*coDel*, (D) *FAP44coDel*, (E) *DYH6coDel*, (F) *DYH7coDel*, (G) rescued *DYH6coDel* and (H) rescued *DYH7coDel* cells recorded for 3.2 s using a video camera. Red bar = 200 μm. Graph representing distance swum in 3.2 s is shown in Fig. 2a. (TIFF 2106 kb)

**Fig. S5.** Tracing of the power (red) and recovery (green) stroke of a single cilium. Selected frames of the Supplementary Movies with marked cilium position. Note that the color line corresponding to the cilium is shifted to the side of the cilium. Red – power stroke, green – recovery stroke. (TIFF 7898 kb)

**Fig. S6:** Fap43p is positioned in close proximity to Fap44p and co-immunoprecipitates with Fap44p. (A, B) Silver-stained gels showing proteins immunoprecipitated using GFP-Trap resin, either from (A) a cytoskeletal fraction of cells overexpressing GFP-Fap43p (GFP overexpressing cells as a control) or from (B) isolated cilia from cells expressing either Fap43p-GFP at the native level or GFP under the control of an uninduced *MTT1* promoter (control). Bands marked by brackets and stars most likely represent GFP-tagged Fap43p (224 kDa) and Fap44p (241 kDa). (C, D) Western blot analysis of the biotinylated proteins in either wild-type cells (C and D, lines to the left) or cells expressing Fap43p-HA-BirA* or Fap44p-HA-BirA*(C, D, lines to the right, respectively) under the control of their native promoters. Note that only one major band of biotinylated protein appears in wild-type cells. Predicted molecular weights of the BirA* tagged proteins: Fap43p, 233 kDa; Fap44p, 250 kDa; Fap57Ap, 187 kDa. (TIFF 897 kb)

**Fig. S7:** Multiple alignment of Fap44p homolog sequences: *Cephus cinctus* (Cc, XP_015588735.1) *Chlamydomonas reinhardtii* (Cr, XP_001695270.1), *Diachasma alloeum* (Da, XP_015121875.1), *Danio rerio* (Dr, XP_017209920.2), *Gallus gallus* (Gg, XP_015152018.1), *Homo sapiens* (Hs, EAW79642.1), *Ichthyophthirius multifiliis* (Im, XP_004031186.1), *Leishmania mexicana* (Lb, XP_003873518.1), *Paramecium tetraurelia* (Pt, XP_001432360.1), *Rattus norvegicus* (Rn, XP_008767007.1), *Tetrahymena thermophila* (Tt, XP_001028010.2, TTHERM_00498220), *Tribolium castaneum* (Tc, XP_008191675.1), *Trypanosoma brucei brucei* (Tb, XP_845979.1), *Volvox carteri f. nagariensis* (Vc, XP_002947240.1) (PDF 105 kb)

**Fig. S8:** Multiple alignment of Fap57p homolog sequences: *Chlamydomonas eustigma* (Che, GAX79654.1), *Danio rerio* (Dr, XP_697139.4), *Drosophila melanogaster* (Dm, AAN71097.1), *Homo sapiens* (Hs, XP_005270577.1), *Tetrahymena thermophila* (TtFAP57A TTHERM_00105300, TtFAP57B TTHERM_00929540, TtFAP57C TTHERM_00052490, TtFAP57D TTHERM_000681920), *Trypanosoma cruzi* (Tc, EKG07864.1), *Xenopus tropicalis* (Xt, XP_002931652.2). (PDF 62 kb)

**Fig. S9:** A C-terminal coiled-coil-domain-containing fragment of Fap44p is sufficient and indispensable for cilia targeting. (A) Schematic representation of the motifs and domains identified in a full-length Fap44p and its truncated versions. The red rectangles represent WD40 repeats as predicted by the SMART and WDSP programs; the blue rectangles represent coiled-coils as predicted by the SMART and COILS programs. (B-D) Immunofluorescence confocal images of cells overexpressing either full-length GFP-tagged Fap44p (B) or HA-tagged truncated versions of this protein (C, D) containing either WD40 repeats (C) or coiled-coils (D). Note that the Fap44p M1-I731 fragment forms cytoplasmic aggregates and is not detected in cilia. (E) Schematic representation of the domains identified in full-length Fap57Ap. (TIFF 1478 kb)

**Fig. S10**: PCR analysis of the *coDel* mutants. PCR analyses of the endogenous *FAP44*, *DYH6*, *DYH7* and *FAP43* loci using specific forward and reverse primers annealing approximately 1 kb upstream and 1 kb downstream, respectively, of the amplified gene fragments cloned into the pMcoDel plasmid. Genomic DNA was isolated from cells of slow-swimming clones and wild-type clones (WT, control). Note that the fragment amplified using wild-type genomic DNA as a template is significantly larger (marked by a star) than the corresponding fragments amplified using genomic DNA isolated from mutants, indicating deletion of a fragment of the targeted gene. (TIFF 7811 kb)

**Fig. S11. Analyses of the** ***Chlamydomonas fap44***, ***fap43*** **and** ***fap244*** **mutants.** Mutation and (partial) gene loss of the *Chlamydomonas* Library Project (CLiP) strains *fap44* (A), *fap43* (B) and *fap244* (C) were verified by PCR. (A) A schematic drawing of the interrupted *fap44* gene with the indicated position of the *fap44* gene-specific primer (*fap44*-1) and an insertion cassette primer (Aph8-1) used to amplify a DNA fragment of 720 bp. Below, the amplification of the DNA fragment in the *fap44* mutant verifies disruption of the *fap44* gene by the insertion cassette. (B, C) The amplification of the DNA fragment in *fap43* (B) and *fap244* (C) mutants with a pair of gene-specific primers shows deletion in the respective *fap43* and *fap244* genes in mutant cells. Control band represents: (A) 5′ UTR region of the *fap44* gene, (B) coding region of the *rib43* gene and (C) coding region of the *fap45* gene. (D, E) *Chlamydomonas fap43* and *fap44* mutants have normal flagella length but reduced swimming speed. (D) DIC images of *Chlamydomonas* WT and *fap44*, *fap43* and *fap244* mutant cells showing normal cilia length for all strains. (E) Swimming speed analysis showing that the *Chlamydomonas* mutants have reduced swimming speed compared to WT. ** indicates a significant difference (*P *< 0.01) between WT and *fap44* and between WT and *fap43* mutants. ns indicates no significant difference between WT and *fap244*. Error bars indicate ± SD. The number of cells analyzed is indicated on the bar graphs. Scale bar: 10 µm. (TIFF 11744 kb)

List of used primers. (DOCX 31 kb)

Mass spectrometry analysis of the proteins that co-immunoprecipitated with GFP-Fap43p, isolated from the cytoskeletal fraction of cells overexpressing GFP-Fap43p. Cells overexpressing GFP protein were used as a control. The table shows the number of all identified peptides and the number of identified unique peptides. Proteins that were present in both the experimental and control samples are highlighted in red. Note that the number of identified peptides of Fap43p and Fap44p is significantly larger than the number of peptides of the other proteins.In *Tetrahymena* cells, the excess of the overexpressed GFP-Fap43p accumulates near the basal bodies (Fig. S2). The identified NUP proteins could be subunits of the ciliary pore. Note also the presence of a kinesin similar to Kif3a and ODA dynein heavy chains, which likely also accumulate near the ciliary base to be transported to the cilia. Proteins identified by only one peptide in the experimental sample are not listed. (XLSX 11 kb)

Mass spectrometry analysis of the proteins that co-immunoprecipitated with Fap43p-GFP. Cilia were isolated either from cells expressing Fap43p-GFP under the control of the native promoter or expressing GFP (control) and processed as described (Material and Methods). The table shows the number of all identified peptides and the number of identified unique peptides. Proteins that were present in both the experimental and control samples are highlighted in red. Note that the number of identified peptides of Fap43p and Fap44p is significantly larger than the number of peptides of the other proteins. (XLSX 12 kb)

Mass spectrometry analysis of the biotinylated proteins in the cilia of Fap43p-BirA* expressing cells. Cilia were isolated either from cells expressing Fap43p-BirA* under the control of the native promoter or wild types (control) and processed as described (Material and Methods). The table shows the number of all identified peptides and the number of identified unique peptides. Proteins that were present in both the experimental and control samples are highlighted in red. Note that the number of identified peptides of Fap43p, Fap44p and Fap57Ap is significantly larger than the number of peptides of the other proteins. Proteins identified by only one specific peptide were not analyzed. (XLSX 26 kb)

Mass spectrometry analysis of the biotinylated proteins in the cilia of Fap44p-BirA* expressing cells. Cilia were isolated either from cells expressing Fap44p-BirA* under the control of the native promoter or wild types (control) and processed as described (Material and Methods). The table shows the number of all identified peptides and the number of identified unique peptides. Proteins that were present in both the experimental and control samples are highlighted in red. Note that the number of identified peptides of Fap43p, Fap44p and Fap57Ap is significantly larger than the number of peptides of the other proteins. Proteins identified by only one specific peptide were not analyzed. (XLSX 24 kb)

Mass spectrometry analysis of the axonemal proteins from Tetrahymena wild type, FAP43 (FAP43-KO and FAP43coDel) and FAP44coDel mutant cells. The table shows the protein molecular mass, the number of all identified peptides and the number of identified unique peptides. Note that Fap43p and Fap44p are missing in mutant but not in wild type axonemes. (XLSX 1022 kb)

Supplementary material 17 (AVI 40507 kb)

Supplementary material 18 (AVI 40507 kb)

Supplementary material 19 (AVI 3131 kb)

Supplementary material 20 (AVI 40507 kb)

Supplementary material 21 (AVI 3157 kb)

Supplementary material 22 (AVI 40507 kb)

Supplementary material 23 (AVI 40507 kb)

Supplementary material 24 (AVI 40507 kb)

Supplementary material 25 (AVI 40507 kb)

Supplementary material 26 (AVI 3157 kb)

Supplementary material 27 (AVI 3157 kb)

Supplementary material 28 (AVI 3641 kb)

Supplementary material 29 (AVI 367 kb)

Supplementary material 30 (AVI 654 kb)

Supplementary material 31 (AVI 3132 kb)

## Abbreviations

(C)Fap: (Cilia- and) flagella-associated protein
IDA: Inner dynein arm
ODA: Outer dynein arm
WDR: WD40-repeat
