# Supplementary material for: Ciliary proteins Fap43 and Fap44 interact with each other and are essential for proper cilia and flagella beating
Source: Cell Mol Life Sci. 2018 Apr 23;75(24):4479–93. doi: 10.1007/s00018-018-2819-7 (PMC6208767; doi:10.1007/s00018-018-2819-7)
Supplement: Supplementary file 1 — Fig. S1: Multiple alignment of Fap43p homolog sequences: Anopheles gambiae (Ag, XP_312857.5), Chlamydomonas reinhardtii (Cr, XP_001698838.1), Ciona intestinalis (Ci, XP_018671542.1), Danio rerio (Dr, XP_697139.4), Diachasma alloeum (Da, XP_015109804.1), Gallus gallus (Gg, XP_015144200.1), Homo sapiens (Hs, XP_005270228.1), Leishmania braziliensis (Lb, XP_001567348.2), Paramecium tetraurelia (Pt, XP_001445436.1), Pseudocohnilembus persalinus (Pp, KRX02904.1), Sinocyclocheilus rhinocerous (Sr, XP_016390257.1), Tetrahymena thermophila (Tt, XP_001017273.3), Trypanosoma brucei gambiense (Tb, XP_011773146.1), Trypanosoma vivax (Tviv, CCD18628.1), Volvox carteri f. nagariensis (Vc, XP_002952887.1), Xenopus tropicalis (Xt, XP_017947697.1). The predicted Chlamydomonas protein sequence was corrected based on an analysis of the whole-genome shotgun sequence (https://phytozome.jgi.doe.gov, Cre16.g691440 | chromosome_16) and led to the identification of a fragment with high homology to the Volvox Fap43 protein. (PDF 112 kb) [file 18_2018_2819_MOESM1_ESM.pdf]

# FAP43

```
     1
Hs   -MAQGRERDEGP-------------HSAGGASLSVRWVQGFPKQNVHFVNDNTICYPCGNYVIFINIETKKKTVLQCS----NGI
Gg   MAAAGGERNPGSPQLSAEALGGRCCSSPEETTLEVSWVQGFSNKNFGFINNQTVCYPCGNYILFLDIKTKKTTALQCQ----TGQ
Xt   ---------------------MSLIRKITIRTAFVRWVQGINKKKVTFINDHTVCYPCGNFIVFHDTNTRKQSFLQCM----TGS
Dr   ----------------------MVAEMDVLRNFEVQWVQGFTNGTFRFVDKKTVCYTCGNFIVFLNVETKERKTLQSP----GSG
Sr   --------------------------MDVLGNLEVRWVQGFNNGTFRFVDKKTTCYTCGNFIVFLNMETKSRKTLQSP----GSG
Ci   --------------------------MQAYGTLDVKWAKGFKGDRLSHVDRDMVCFSCGNSIKFVNVVTGAESVFQPP----GGA
Tb   -------------------------------MATNARILGFNSPSALVAAGDDFLLGAGGGIVIRRKEESSQWIPCEG----RYA
Tviv -------------------------------MSSNAVILGFNSRSSVAVAGDSVLLGAGAGVIVREGDESSQWIPGDG----RYH
Vc   ------------------------------MAFQTKVAIGYNGSPVAWLGSEEVAWTCGNALVLQSLNTKTQRVLKGT----GFG
Cr   ------------------------------MSIQTRSAVGYSGGQVTWTGPDECAWTCGNAVVLHTLGSKAQRVLKGT----GFG
Tt   --------------------------------MSIYESFSYNGSPFQLVFNDTIGFIQGSTLVLWDLATDKKDYIHSQ----KNG
Pp   --------------------------------MSIHSSYCYDGSPFQLAFKDTIAYISGGVLYFWDTQNNKKDYIQSS----KNG
Pt   -------------------------MFSKKWFNLFSDCTAFTGDKWELLEQDTIGFLAGNTLCKWNLENNSKSFVHSQ----RQG
Lb   ------------------------------MLQSVARVLGCSAPAVFAVHGNRVFVPADNGVVVVEDSYEYLWAPFPI---GKYS
Ag   --------------------------MEQNIRTKALWINPGAVEMACVVGNAAVAIAMGGHILFVNLKTTAKSYYSADSVHSGNG
Da   -----------------------------MPHAETKWVKFGDIRTIAFMGKETIAMASGLHIIFINLNTKEERVEKFDSKERGEG

     86
Hs   VGVMATNIPCEVVAFSDRKLKPLIYVYSFPGLTRRTKLKG------------------------------NILLDYTLLSFSYCG
Gg   VGAFAVNPNRGVLAFSDRKLNPVIYIYCFPELNKLTELKG------------------------------HIQLDYTLLAFSFLG
Xt   IGAFSVNTYSEVVAFSDQKLHPSIYVYTFPGFVKRVELKE------------------------------GAQLDYSLIAFSNAA
Dr   IGAFTASGQRRCIAFSDLGLKPSIFIYKYPELELTSELKG------------------------------TTKLGYTALALCDTG
Sr   IGAFTASGHRRCLAFSDLKLNPSIFVYNYPELEQVCKLKG------------------------------TTKLGYTALALCDTG
Ci   VCQVTSSSHHRMFAVAEEGLNPIITIYNFPSCEVVCALEA------------------------------GALLGYSQLQFSFNG
Tb   IGALAFSPSAGLLCVTEVKLDVSLHVFRFPERHHLQCIDN------------------------------VATVDVQHMLFSSDG
Tviv IGVMAFCPETSVLCLTEVRLNVSLLVFQLPDCDRLQKIDN------------------------------VATVDVEDMLFSPDG
Vc   ISCFTVSKRYGLIAVAEKALKPNVYIYAIKSLQLLAKLSPSELSKE------EEHALPGGSSEKQQQAN-VVTLGITAMGFSGDG
Cr   ISCFAVNKRHGLLAIAEKGLKPMVTVYSTKTLQPLAKLLPGDVREEGAGGGADKPSGSGAVSGKQQSSGPSVVLGVTCLAFSNDG
Tt   FQSFVAHPKKGIIVVAEYSLNPKVFIYSYPQKKLLRTLEN------------------------------VSELEIIQMDISFDG
Pp   FQCFTCHHSKQLVFAADYGLKPVVQVFTYPEKKQIQAKCV------------------------------ISDLQIIQMDVSVDA
Pt   FQCFTVHYRKNILVVAEYGLNPIVHVYDNENE---YDLVG------------------------------VSPLEILQMEVSNCG
Lb   IDKIAVS-AAGVLCLAEKRLHVALHFFDADSMQSLGVAEA------------------------------EVFVSIADIVFSADG
Ag   VACIAGHKLFPIVAFAERCTNPRILLVSYPANTVLSILEG-----------------------------DQTECSYTALCFSES-
Da   ACCLAGHPVVPMFAISERRANPKVRVYTYPSVNKVSECIY-----------------------------GERNWTGFISSAFAGT

     171
Hs   TYLASYSSLPEFELALWNWESSIILCKKSQPG-MDVNQMSFNPMNWRQLCLSSPSTVSVWTIERSNQEHCFRARSVKLPLEDGSF
Gg   PYLASYSSFPDFVLSVWNWQENILLCSESQPG-VAATSISFNPMNWQQLCFVTESSVTIWCIERNYDEHCLTQNPVKLPDGRGSL
Xt   PYLASFSSVPDHVLTIWNWQEGIPLCSQSESQ-TTYTTLTFNPMNWHQLCLSSETSLIVWNIEICDNLYQLKAMPVKLPSEDGTI
Dr   PYLVSASPMPDHIITLWNWESGLPLCSHSLLD-EDISALVFNPMNWCQICAIKSKSLTIWNVERCDNYHLMKPSAVNLPGTDGSA
Sr   PYLVCASSMPDHTISLWNWESSVLLCSHPLLG-EDVTALVFNPMNWCQIGAVNLRSLTIWNVERCDNYHLMKPSAVDLPAADGAV
Ci   SFFAATSSIPDFSIFVWDWQTGKLKCHEKLPGESPVTSLLFNPLNKREISTISETHVSIWTLEECNEISTMKSKVVNLPTTDGVI
Tb   EMLALLTCIPTTCVTFYSAARGNRLVKCASTEL--------GGVFCKHLTFPLHRHDCIAVLEPHGVRIACNMDSATFVPSILTL
Tviv DFLAMLTCMPSPCVTVFDVQHGMTLKKIHTIAL--------ESKFWKSLAFPPQPENSLAILESKSVMIATNVVSEFTTPIVLKL
Vc   ERLVICGDEPDCSVIVYSWRKGEVHGRSRLP----------HNQPAHQVSFHPLDPTILATTHSG-TVSVWYLEAMWDRTLFRYQ
Cr   ERLAVCGDEPDCSVIVYGWRKAEVLGRCRMPAA---ASSASSTSPASSVSFHPLDASVLATSGPGGACAVWFLEPLWEKAVFRPL
Tt   KKLLVVNGVPKFEIQVYDLETGQRLSGT--------NSSIPLRNKFIKAQFSPSKDTTFCVLYEN-NLVLCEIFPSFEVDQNSQE
Pp   NKLLVVSEVPNYEITVFDTSNCWLSDKNVIRRLEGEYSSVHLKSGFIKAQFSPQNNNIFAVLYEN-VLELYEILPTYEFVNGRQE
Pt   RYLLVILGVPKFEICLWDLKEKCRI--------KGKFSQLPLKLNFIEAKFTILG-ERILIRYAN-QLQLYQITPHYDNQIQKQV
Lb   KELFVLGTVPDTTVTIFRRSTP-------------------NGSYSLADRVPLQHSEEPKGLLAFGFEGSVVRFGIWRSGCIAGY
Ag   ELLVALTGVPDYSLEVYAWRSKELLCK---------------KPSSIFCEVQRLNCSPSAVFAVCQYAPRKPDLKLWEVH-----
Da   DYLLTLTSSPKFKAVVWLWRTG---------------ECLNTVDTGVSDEYQTMLCSPTSPPLITQLGRPSGRLKLYELN-----

     256
Hs   FNETDVVFPQSLPKDLIYGPVLPLSAIAGLVGKEAETFRPKDDLY---PLLHPTMHCWTPTSDLYIGCEEGHLLMINGDTLQVTV
Gg   N-HEDLFFPYSCDEDPYHGPVMPVSAIAGLVGDKAETFVPRTDIK---PSLHPTAHCWTATSDIYLGCKEGYILAIDTRKCSASI
Xt   GVEEENFNSSAPNGVSYYGPVMPKSAVAGLVGEEAEIFIPKEQRK---HSVQPSFHCWNATSDLYVGCERGKILTISAETQKVIV
Dr   IECEPNQSHLLNGKLTYLGPQMPTSAIAGLIGDQADNFVPLKLIK---PRLCPSAICWSISSDLYVGCKEGFLLCVNPETLHVSV
Sr   IECEANLSCMLSRKLTHLGPQMPISAIAGLSGDRADDFVPLKQIK---PKLCPSAICWSTSSDLYVGSKEGFLLCVDPDTLLVSV
Ci   LSNGEDLASRSGTRQG-FNVHLPTSTRAGLVGGNADKFEPPELRR---PRVIPSSQCWSSRGEIYVGCVGGQLFRVDGASGNPAL
Tb   ------SSKGHYFHSCVWGTEGLYCGA-----GRGQVVLLDELR----TDMKNYINCETPHNVTALLQN--GTLLFIG-------
Tviv ------DSNGEEFHSFAWGPEGVFCGA-----DNGSVILFDELK----MEMQILLTIDTKCAIMALHRT--KAVLLVG-------
Vc   SLGPGALPPGHDVTVHAWSPHGLYVGT-----SGGGLALLDLPSM---TPLVIKGASAVASAAASEPPSPGGTAGGGGAGGG---
Cr   QLAAGALPAGAEVTCHAWGPGGLYVGT-----STGGLVLLDTATM---APLQLHAAAGGAAGEVAGGASGGASSPTPGASSG---
Tt   VLQN--IRIDQNNKISEKSTFKNFIWD-----EQNNIYLADEY--------FVRYLNGTDLSEIMNKDCEYLVEFFVLTQKHLII
Pp   ELDKK-IRINKKASYEAEGQFSTFIWD-----EQNRIYLADKFNIVYLDIVFDQELNQNELKPKLTHESFHQITHFVLTQKHIIV
Pt   QLELVT-SIDLSAPTIMVQDYN-LIDDSKNQFEQNNIYLIQG--------NVLIYLDGTNLTEIARHECHSEIKHLIPTQIHLIV
Lb   ------AHGEDGKCFQKCFSVEASIESACVSGPSTMCYITKE------GSLHVYSHESRTFQDVCEGSLAPAAASMIVWDGTVFI
Ag   ------------------------GNIRISRLIERTVHLELE---------------TAELPLCVTFLIDGNLAVVTQRAKVFII
Da   -------------------ICSKIVSLTPASLAPSPESRKSE---------KITSSSWTYDGNLLLSDDLGNVYLTPTDGKRRQQ
```

```
      341
Hs    LN------------------KIEEESPLEDRRNFIS-------------------------------PVTLVYQKEGVLASGIDG
Gg    IQRKPLPEHMLKISDMLGYIRREAQKKKDGKPKALQRT----------------------------VVFTMAFCDEGLYTAGIEG
Xt    LAQ-----------------KDQDADPLSGVTLLEGN------------------------------IKTMAFHKEGLYIAGKDG
Dr    LYK-----------------PQTEVT-TGNSEMPMQEG----------------------------SFQSLVLQDSKLFAAGMES
Sr    LYK-----------------PQTELNPTDDSKTSLQEG----------------------------SFQSLVIQDCNVFAAGMEG
Ci    LLG------------------GISDSLLVGEIECG-------------------------------MFHSISLNSEGLVAGGDDG
Tb    ---------------------TECGDVFTYNIDQKAQR--------------------LLVRLGRSVVRLLTLPDVNDVLVATST
Tviv  ---------------------TQRGVIFSYDVDKRELK--------------------RMISVRHAVERFLACDDESNVLVVSST
Vc    ---------------------SAAGAFAVAMTAAAAAAGVAPGQPAVVLDAAGPGAGVSALALNRDHLAVCGTDGSVHVFSQAPV
Cr    ---------------------GAAGGSVVAVVPDAVTS----------------GAAVTALTLNRDLVAVAGADGSVRVFVSVPA
Tt    IL-------------------QNGKFEWIYKYDPANLN--------------------EDEIKPFKLEKTY-LYDEEKLVNILYN
Pp    IH-------------------ENGLIEWLFKYHPKFEN--------------------DEDQKPFKFDKRY-QYQDEKIINILYN
Pt    VY-------------------ANAKIEWLYKYITNNLE--------------------DKVAVPFKVNKKYNLHDHIEVKKIMYN
Lb    FTR------------------SGDLLSVNLCDGTHHRR----------------------------SLGCLPSCASRLVVTSAAE
Ag    LAS------------------TGQISQTICNPEPLTTD------------------------------QEY--IPYVFYCKGGLF
Da    VLQ------------------SEETTPHLAGPLVVAFR-----------------------------GGVAVANTSPKILFYRKP

      426
Hs    FVYSFIIK--------DRSYMIEDFLEIERPVEHMTFS-PNYTVLLIQTDKGSVYIYTFGKE-----------PTLNKVLDACDG
Gg    ILYFYHIR--------DLQYEMKICADISEPISTLTFS-PDYTTLLIVTDQGTIYTYEPAHS-----------GEAVKLLTAFSS
Xt    VLRSCTIK--------GSELKLEDCWSVQESIESISFS-PNYKMLSIATCKGSVYLYNHRNP-----------GETYKVFDVYSG
Dr    VLRSIQIK--------GNKLEVVQTWALEEAASAMCFS-PDGETLLLTSNTGCVYRFKPLLK-----------DKAVKVLDVLCG
Sr    ILRNIQIK--------GNKLEVVQTWALNEAASSICCP-PDGETLLIVSNTGCVYRLKPLLN-----------DNIVKVLDVLCG
Ci    TLRLIQLD--------NNGVKITELHKGMQPITCLSFAPPLYVDLVLSTSSGEIQHYSLVER------------ECGPIITTRTG
Tb    DVTKISVD------TAQSVFVRRRSASDTVKLLVLGG------LVVIVCLDGSLVTYDQDTNTAG-----HTPVRFPEKVVDACV
Tviv  DVMKLDIN------LASSTTISSRNTGNTVKVLVIGS------LVVCVSSDGCFMLYEHETNTAI-----HIAVHFAEKAMDACV
Vc    AEALGPPG------FSHEVWLARGG-STGVPVSSADCGGPEHTALLLGCPDGTIYCAPMGPHTGAG-VTSRAGYTTATQIGDYPV
Cr    AAAAITPEPLA---LSHEVWLGRAGPARGVAVASAECGGADHATLLLGCPDGTMYRAPLAPKQGG-------GPTHSVLVVDCHV
Tt    QTYSKLYAG-------SNQGSIIIFPVEGETFDIEEEEEQQDKENENHSDNEDESREIEIQTEKKG-------PYPFSPIVFIRE
Pp    QDFTKMVAG-------TQEGSLLILPEVAETLEGEEEEENLDNEQE--GSDEEKSREIDIDLQTYG-------PFHVGSVIFIKE
Pt    QQCTKLIWF-------IRQCYSQYNPYEAQVDQQEE--------------DKVIDLTHEIEPVKLG-------PYQKGVITFIRE
Lb    LLLSTQTGL-----LAVAVPVVTNGGAADSRLVKGWTEASTLRCLSVNNGASAAWVLRDGSIALYK-------RDDVRVLTPCGV
Ag    VSAPDGQVNFY-----KKQKGTWNQMWSTQSDASYSLLLSYSTAEGLLGITTEGFIMRTVLDTDVR-------NVEFQIVKDLDV
Da    PAERVKSG------------SPWKIIWEIETSSPVLYLSRH-PQRDSILLCSIKGEISEVSVGDDD--------IPRLQIICQQG

      511
Hs    KFQAIDFITPGTQYFMTLTYSGEICVWWLEDCACVSKIYLNTLA-TVLACCPSSL-SAAVGTEDGSVYFISVYDKE---------
Gg    NLLAADFLSPGDKYCVSVTISGEVQVWLLENGICLSMLNLDIEA-TAMACCPSSN-SAAVGTKGGQIYFIDITKVE---------
Xt    DLLAADFLTFGNKYCLSTDISGHVQLWSVEDGRSVSSLSLNIQA-TAMACCPSSN-YAAVGSSTGHIYFIDAMKIE---------
Dr    DFVTVAPVYTDRSICLSVREAGVVQLWTLDDGLCMGSISLQTHV-TSMACCPIAQ-YIVVGTATGDVLFVEMTTKQ---------
Sr    DFVAVAPLYTDRSICLSVRETGDVQLWALDDGLCMGSISLQIHV-TSVACCPIAQ-YVVVGTASGDVLFVEMTTKQ---------
Ci    DFVGAGFTC-AGKLAVTCREDGELQIWSCEDGSLVASFSLCTRT-CSLSCCPSSP-VVAIGTWTGHVCYIDTSSLLSEEI-----
Tb    VGSVAVVVYDSG----------FVRSFTVENTVSVVS-QMKVSD-CPLTACTSDGVSVLAVCDKNVVHFIEVADG----------
Tviv  VNSVLVVAYDSG----------WVRCFTLNSDATVMTSQTRVSE-SPLHMCLSDGGSLVAVCDKSVIHYLSVKDG----------
Vc    GRLAGIVPHPGGGAFLTAGADGSVRLWAAEDGRLLARKALSSAQ-CALAAAAPGAGLAAVGSETGVVRVLVLPPAPTSGSDAVSG
Cr    GRLAGVVPHPGGGAFLTTGSDGSVRVWSTTDGALLGRKQLSSAQ-TALAAAAPGASLAAVGSETGVVRVLVLPPASTAASAAASD
Tt    LENQNVVITVTQNS--------YIYFWDIEKKIQVSTFKLNCTI-VCGDIHPNSK-TLILGSSSGVIRILDISNLN---------
Pp    LKKMNCVLTISSEG--------RLFLWNIQKQQLVFSHQFKAQM--SSACLDNSESILLIGSQQGVIRILDVSDFQ---------
Pt    YKQHNVIVCGSDQG--------VVLFMDVISKQPLSSFNIEGKI-ISGELIEPN---LIIGTSAGVLRFYNVADIR---------
Lb    RRAAVHACILNSSEIVILFEDGTLRCFDCVGEREVWSHQCLEWA-PTFVEADGSG--TVACCGRDALRFLRCMDDG---------
Ag    GYKFFNNCLNDAQQQLVGIKPAAGTVKLLQLETGAAGDTITVGR---VTSIAHHPEMPFFVVGSDTGYLHLVAVDGKQ-------
Da    SGHLFLVSVHPGRFIATVDSFDGFNLFESLTGKLTGRMWLGEYGRALSVLSHPVLPLLAISTDTGRCLILNVAEAS---------

      596
Hs    ------SPQVVHKAFLSESSVQHVVYDQQGIFLLVGTAEG-KVFIINANS--SSSFQIIGFTEVAKDILQISTVSLLETDIVEVM
Gg    ------APRVVHRIFLSKFPVLSLHYDQSGQFLIIRAMEG-YIFILDARP--SKLFQVLGYVVLAGEVLSLSVVSDFKNNLVEVI
Xt    ------ALRVVQRMRLYCVPVQHIHFDPRGNFLLTGAADR-HIFILDARP--SHSFEVLGYIVVCGEILTLSSLSSEDSQQTKAM
Dr    ------KPRLVHRVHLYHVPVDHLVFDQGGNCLITGASDS-RVFVLDSRP--SKGFDIIGWIEAPGAIVGLSTQYHQESKQIKVL
Sr    ------KPRLVHRVHLYHVPVDHLVFDQGGNFLITGASDS-RMFVLDARP--SKGFEIIGWIEAPGAIVNLSTQYYTESKQVKVL
Ci    ------NLRQVGSTRIHHSPVTYLKYNAGGDFLLTASEDEPHIFIIDAKP--SSGFQCIGYVDCDGTIKDISIHHN--SSTSTVL
Tb    ------LLETAASSDIFACAVTNLRWAVNGGRSVLAACNNGEVHNLCFTG-KCDSASAGVTVDMTWRLDFPVNDFLPLYG-----
Tviv  ------VLQLQTTSEAIASTITNLCWSANNEQSLLAACSNGEIYLIKYFE-SDDLGAPAAVIDVIWRLDFPVTELLPLFA-----
Vc    GTQPSQQLRVMYRRRLHTSAIDALAFSPNND-LLLSAGRDGAVWLLALDA-RTGSCRALGFVTLPPGERVMAVTWPRAG------
Cr    APLP--ALRVLFRQRLHSAPVDVLVFSPAND-LLLSAGRDGIAWLCSVDA-RGGRVRPLGCLSLPPGERVLSATWPRSDGGSGAA
Tt    -----GVHLLKQIKVLKDKPVSNLHFNPDGSMFFVSSTESKKIYLINS-----TKYNIIGYIALPAKVNTACWNTSQKLV-----
Pp    -----KIQMVKKIRLFKNKAIEFLEFSKDQSLILAGSSDSKKAFFLSTQNSRKKPYRVVGYSKLPYKINYLTWNSSNVHK-----
Pt    -----QPVLFKMIKLYIDKAISSISIIDNN--LAVCSSDSPTVFFFQN-------ENLIGFVDLPFNCQAITYG-----------
Lb    -----VEDRGVVRTTLLASICLVRWVPMETCLLVVCQNGDAFLVEVPKD-------GDAETTHTAEAFVRSSWRLDFPITDALVC
Ag    -------VKLFGRLYLSRHPIVSVVFAIADSRFFGAVDATGQIAILELSDG--WQMNVLQVFGELGKAKHLFMWCS---------
Da    ------DPLVHNCFHLIREPLDAAKFSQSGRVLGVCSTRAGRIFIVRGFEG---NIHVWGLLEISEKIADYLIYED---------
```

```
     681
Hs   VLSS----------LPEAGRSRLEMFTLPTLLPQVS--TTFADERGRLKDEIIH------------------------------K
Gg   VLLS----------VAENQQTRLEIFCLSSALMEDI--DKYVNDQGMLNVSAIK------------------------------K
Xt   ALICPV-----NEGKEEKGGTRLEMFSLPLQMLSSP--SEYIDEKGMFKDAMVQ------------------------------K
Dr   VLCN-------KSEKEISEGNVLLLLTLFAQQLTES--TSCMDAHGCLRKEVFD------------------------------S
Sr   VLCS-------KTEREINEGNVLLLLTLFVQQLTDS--SGCVDVYGCLCKDALH------------------------------S
Ci   AVMSQ------SNDNGDYDSNNLIMFSLTSELLSNTD-GHFVSLRNNFKDTSVK----------------------------LTR
Tb   ------------------DGDVINIFVHSVDKDTKMYALERQRVKESKPLRPYF-----------------------------LM
Tviv ------------------DDDVINILVHSVDKDTKLYVLERGRDKEGKPLRPLF-----------------------------LM
Vc   ------------SDVLDGESAFISLAGGGIMSLTASAELTSGNWRNPNPDLQLVRPTAVAAHGSRSLGSDNGDSGPSGAPEPVVV
Cr   SGHAQAGPVSTTSAEGDEPSCLLSLAGGGLMCLTASAELHSGNWRNPYPDMVLL----------------------------VTI
Tt   --------------IPTHKNVLFVLLNYFLISLIVPDATYQNKDQLKIDNDVCP------------------------------V
Pp   --------------PNQGKTSAFALVNFFLIAIIPPNPDEEYK-ELNLGEEVCP------------------------------L
Pt   ------------------KGQLFCICSFLLLSIPQPKQQSSLKLEVQI-------------------------------------
Lb   YVT---------------PDVLNLFVHSADHDSKVYMLDRQREGDGKVSRPLF------------------------------LI
Ag   ------------------SQEVLVVQPLETEDSISFSSIEFIVKQHSLEETTRT-----------------------------QL
Da   -------------------ETVLTLVQSNKHVAVGKYIAVYKTTGPDYFELRD------------------------------VV

     766
Hs   YLYELEHALSSAVLGFQSNQ--IYGFCSQVPYICSYLLPEEEHTG------IYILKPYKKVQSRQYGPGLLYLSSHGLWLITIAK
Gg   EQYDLEYPLSSAVR-LKDDI--VYGYCTCAPFICKYHLSKQSILED-----PPVFLSEKMIPSNQFGAGFICLSPNSRWLASAAK
Xt   ISYDVDQPLFSAVLGYNVSS--VFGFGSYAPMLLKYVIHKEISGDP-----LTALVTEKVVRGSHLGPGVLCLSPHLKWISVSGR
Dr   CVLELPNSLCSCVL--AVNK--IFGYCQQRKVLQRFSIPENGENPNN----VVQLIPEKETGGHLLSPAFLQLSPHHTWLASVGR
Sr   CLYEIPHALCSCVL--AINK--IFGYCQQRKVLQSFRIPESGVKPSNAQE-AVHLILEKEAGGHLQSPAFLQLSPHQSWLATVGR
Ci   HSLSVSVDSAIVTTIGDNIE--IAALSSESKNLLQFKLPHTTQG-------PTPIHPVSEHYGHQLSGGSIILSPHGKWVASFAP
Tb   RDHECGGNVLQRLGGDSIISAGGDGRVVVRDISHYLMKLPPVPPTKEK---KHPLKEFLLRPFGRGGITCLSVWNAAGGFVCGGN
Tviv RDHESVGTVLQRLGDEKVISAGVDGRVVVRNISHYLAKLTPIPPSKEK---RNPICDYALRPFGKGGIRTLCVWNDTGGFICGGN
Vc   RLLRLEVPVLAMTTAPHDRTGELYGLGADKQLHKVALPAEAAAWAGLR---ARPFRSSVHVPAHARAGGGITMAPGGHVLVSGGA
Cr   KLLRLEVAMLAVAAVPGDRYGDAYGLGADKQLHKLVLPAEAAAWAGLR---ARPLRSAQHVPAHARASGGVAVAPGGHLLASGAA
Tt   YARKIDPDMNHIAVNQVTGDILMTGKDKIVKRYKQPEELYAKMDTRIK---VGGAP-IEEQDGHPLPTNAMIISDQFNLLITGGQ
Pp   FGRKTDNNLNLLVVHPRTGDVITTGKDKMLKRYQQPDKLYQTLDLKLK---AGEDP-LDEVDGHSLQTNIMRIHSQNNQLISGAQ
Pt   MGRKIDPDQTLLIVTPQQ-EVITTGKDKIFKKYKFPEELLSKMDLKMR---VANQPPVDEQDGHQLPATCLAIKD---FLYSSAK
Lb   HDHSSGGSCLLRLNDSTVISCGRDGSIAARDLTPYQTQMTPIPPSREK---RKPLWVHAVRSSFGGGITTVATTDTGAEVICGGN
Ag   RLPKDYCHIIPKCAGTNLNFYAYEIRTNVIDLLEITRTDADIDVVLLR------------TIKTPLAVSHLELCVDDRYLYVWSM
Da   KLPRVYASLHCGSDFGEIIASPFLSKQLHLIKSEDNFDKFILTEALS----------------SSHRLRDVKINADTRYVVTFGY

     851
Hs   CGILCIRDVYTLET--------FAWCRSHSHQGHGIQSMRISMDGQNILVNGRDDGTLVYL--KWKRFGGHLASEILDYYQKLLI
Gg   DGVLFIYDTSTMEI--------LAQNYCHSYEGGGIRSMVFSLDGKFILVNGENDGTLVCL--KWKKIKEIEVKEADFHWHSLLT
Xt   DGIVYVKDIKNMET--------VAQVQCHSYHTGGINSLSFSLDGQSIITTGTFDGALVCL--QWKSTGSSRLGAAVEYGKAFTL
Dr   DGLLRICEISEMDR--------YVQLQCHSCWQGGVGSVCFTPDSQTIITSGLRDGSLVCS--RLKLSGVGKANAATQYSQSVAD
Sr   DGLLRICEISTMDR--------YVQLQCHSCWQGGVGSVCFTPDSQTLITSGLRDGSLVCSRLRLKLSGAGKTNAATQYAQSVAD
Ci   DGDVIVRDVSNWDS--------ITRFQPHSYIHGGVKTLTFSPDASHFISVGYSDQACMCYQWNFTSKGQSAGNNAVEYARVLNM
Tb   DSVVHLVP--------------VGKSPIHYSWSEPFWHQRAISTSPSRASSP-SDAETLSAER----------------------
Tviv DTVIHIIP--------------NGSANIEYSWTEPVWTQRSFSIAASGCSSP-LKSEHNILGQ----------------------
Vc   DGVIAMRNLNLAALGDSSAGGGIGGSVLHDVTAGGVVSVSFDATGRYFASAG-ADGAIVVFEVGPAAGAAHLLVTPPWPAVPFAG
Cr   DGTVALRNMSLITLAAQQGGDGAG-AGLHDITAGGVVTVSFDATGRYLASAG-ADGALFVYEL----------------------
Tt   DGSIYLRNLEKLSE--------FQQIKSHNWKINGVSTLDFSKKYKLLYSGG-YDGSFFAWSLD---KVSFQNIQRN-YEILRED
Pp   DGTIFIRDLDNLMD--------YHQIKGHNLKYRGVSYLEFSQKYPLCYSGGNGDGSFFVWKFQNEIMFDQDNIELPPQKVLEEN
Pt   DGTIMFRSYQQLNQD-------VRLLRGHNLKSGGVSTIYVSQKFKMIYSGG-FEGDLFWWTGEK--IRGDSDAAQLMKSNYNAP
Lb   DGVIQCVTLKAEGA--------HATWQEPQWTHAEAARLGIVGDKITTVQAG---------------------------------
Ag   DGKIALYELLTVLCH-------SGMLTVLKLLQPIPQSPNTMSNTSKSLPCEAENSSLLDETVIDAAVPWIEQAERERWEEKGK-
Da   DGLVVIRNRSNLSQL-------IAVLTTHHRQEGGVKHAVPCPCNSILVTLGKNGNLVASKISQLESAEVGDHVTSFADAKVVLS

     936
Hs   SLSSAMDKENHYLSTTPKVSVDLGS-DSE----HTKQK----ASTDLSQDELVLTD----VKKEIPWIQQKSQE-----------
Gg   ILNKSISDENAVLRLMAEWQLESES-TSESLPEEKSKKSPLEPSSAAVTEDGSFTSLHSDSASEMTWLGQKTEK-----------
Xt   SLQAAMKEENQMLSRMPEWTADTSS-MAVESKEKEYRN-----LSVDVTEQDTFPN----TSADVTWIYRKLEEVRQRNLCIYSR
Dr   SFESVVSSENTVLTKMTDRD------SFHTGEVSLKGK----MSHEAEQEENYFTS-----PTSYTWLDGKLDA-----------
Sr   SFESVVSSENTILSKMTDWDPQTQSLTRCSSFLTREGK----TSHEAEQEENYFVS-----PASSTWLDGKLDA-----------
Ci   RLRSVMDVEGDVLAAMEEW-SPSTKIATPDQEEQDDSD--VTRPKSGARSEVTMTP-TPTPQSDATWLDARSLL-----------
Tb   -------------------------------------------------------------------------------------
Tviv -------------------------------------------------------------------------------------
Vc   SVTAASRVEADAIDDASELTEVELVRRQGALETGGING-----------------------------------------------
Cr   -------------------------------------------------------------------------------------
Tt   VNIQDLPDEKVMHYQKVLEEELIKSKKEVN-------------------------------------------------------
Pp   QMIMDVQDNEVIHYQQELQEEFIRSQKEIR-------------------------------------------------------
Pt   MEIQDMPDQEVRYYQQVLESEFLEQQAPIR-------------------------------------------------------
Lb   -------------------------------------------------------------------------------------
Ag   -------------------------------------------------------------------------------------
Da   NSTGVELDGDGDSTWIELKEIRKLERERN--------------------------------------------------------
```

```
    1021
Hs    -------------------------------AIKKEVNLFSKKRKEIKQGIKSLSKTILNMMEENDK-LENIAKLDQQEFGLDLE
Gg    -------------------------------DIREETERFANQKEKLQMGIKKLRKTIQKMMHENEQ-VPDIEKLGQQEFNLDME
Xt    KCCVNSIFWKRNDNGYVLNTNVAVWVKLHLFAKKKETEKYAAEKEIIKTGIKKLRKTIQAMMRENES-LPDIEKLDQQEFNLDTE
Dr    -------------------------------VLQEEAQQYAETKKNLRRNIKVLRDTIKAMMEENES-LADIEKLEQQEFNLDVE
Sr    -------------------------------VLQEETQQYAETKKNLRKNIKVLRDTIKAMMQENEN-LSDMEKLGHQEFNLDVE
Ci    -------------------------------AVKEENAKYDEVKRDLRKNIKELRKTILGMMENNNS-LPELEQLRHDEYNLDTD
Tb    ------------------------------------------SRCRIISALADLRMEVEKLLQERTP-TVRAEDFLLP------E
Tviv  ------------------------------------------SKSYILSTLSELREDVGKLLVERTA-AVRAEDFLLP------E
Vc    ----------------------------------------ESKRAAVVSKLMSLRSRIGELLEANQA-VPELERLERSELIVDVG
Cr    -----------------------------------------SKRAAVANRLATLRSRIADLLAANEA-APELERLTRNDMIVDVG
Tt    ----------------------------------------EQSKKVIEEGLKKIQNKLLELLDHNKNEAEELEKLERDDFVIDID
Pp    ----------------------------------------EKQKQEIQSNLMGIKSELEVLLNNNEN-AEELEKLKRDDFVIDIE
Pt    ----------------------------------------EQQKRQTKDLLKQIQTKLNALLQENAE-SDELEKLQRDEFVIDLY
Lb    --------------------------------------DAAPEREALLLAVSRVRELWMKVMSDKDT-EVPLEAFLTP------E
Ag    --------------------------------------IYEPEHEAIFAEFGEIKHQLKELLNHNER-APVEERFPLQVFNLNAE
Da    --------------------------------------EMAIERASILSDFEKLRDRLKALIDDNEK-ASEAAKLPLENFDLDKE

    1106
Hs    ELERLHDESQEEVAKMIKDVEMHNLAKSYLAELIKEECWNSMAVKGRALKCFHIP-CVVENFPMKARTVEELKELERVLQQKKIE
Gg    EQERAEAEAEQEVARVREKIEMEILANCYLQDVIKHECWDSMCVKGRAVKCFRIA-CEVKNYPLKKRDEEELRTLEKVLRLRNIE
Xt    EQERLYLESEKEVARVRQEIELENLSKQYLREVIKQECWDSMAVKGRSVMAFHTG-YEVMNYPMKERTPKELEDFARVLNLKKIE
Dr    EQQRLQLEGDQEVNRVRKEIEMENLAKSYQREVLKRECWDSMQVKGKAIKAFHSE-NEVKNYPMKERTAKELEELRMVENMRQIE
Sr    EQQRMQLEGKQEVTRVRKEIEMENLAKCYQREILKKECWDSMQVKGKAIKAFHSE-NEVKNYPMKERTEKELEELQRIETMRQIE
Ci    EQSKMQEQQDQRVQEIREEIEFENLAQRYLAYLITKQCWDDMVTK-------------VVLSPTTQPPVFPVKKMQSETGTK---
Tb    QRQAFNEECEMEIHKAREDDYYSLVHNEFVQHTIKTECWDVMEVQRSKIVSMTDPETEVHNFHLRKPCAQRAKIQKKIKLMRAIQ
Tviv  QRDNFNADCVTAIQEAKEDDYYRMVHNEFVQHAIRRDCWNTMEVPQSKIVSLTNLESEVFNFQLRKADPTATNILRKIQFLRKLQ
Vc    LVERLKHDTEARVAAVAAAVRRNNLRTELLAERLRRMVWDHLAVKGAVVSGLRSSL--VVNNFPLTQPGPHERVLRQVTLLRRVE
Cr    LVEELQRETENRVAAVQAAVKKEHLRTELLAERVRRMVWDSMAVKGAVISGLR------------APPGQHERVLKQVTMLRRVE
Tt    TKNKILDSGKQQRKQIKETAKRENLKQEIIHSKIKEKTWDQMAVQLKGINGLKSK-YLVYNYHIRKRTADEERKLKIVKDLRRIE
Pp    TRDQILKQGEIECEKIRENAQKENYKQEILHNRLLEKTWNTMKTHLKAINGLDTN-IIVYNYHIRQRSEKELQQLKWIQNLRKIE
Pt    ARDAILEDGKKQQQALRDLVKKENVKQEILYQLIKERTWDQIEIPLKGVNGLIQH-IIVTNYHIRVRQSEETRKLKLIRELRKQE
Lb    QRDQFAAECDSAVFDMREGHYYHAILNEYLQDTLRRRCCEAMEVTRMKVVSMKTSELEVHNFHIHRRTRAEASLSRKALFLRQLQ
Ag    ATEKLTQEANHTKDEEKKRLQQFIESQKDINEKLIENCWMIMTKKPWKIRPMFKK-AFVENYAIVPANEYR-QHLEKVRVYRETE
Da    GRARAIENNREEQKNLVRKLEEKASREAEISEFLRKLCWDSMQVPACALISLDGR-VRVDNYALPRISDQEEERRAWAKFYVDLS

    1191
Hs    AECLKLRKEIVEAQSGVKLIKQRHEE-----------------------------------------------------------
Gg    AADLKVQEKVLETKSKTVLFKEEN-------------------------------------------------------------
Xt    AVDLKVRKEIVETQPKIKADDEEEVE-----------------------------------------------------------
Dr    QEDSQLQEGVLDTLFGDKEEE----------------------------------------------------------------
Sr    QENSQLQEMILNTISGDKEQEEAE-------------------------------------------------------------
Ci    -------------------------------------------------------------------------------------
Tb    IKTEECFTLSSLVKRAKEGNLCTEQQACGP-------------------------------------------------------
Tviv  LKVGDHFTFASVKFGNKEGEVGEIPAAPQD-------------------------------------------------------
Vc    LLEHQALGVVPYN------------------------------------------------------------------------
Cr    LAEQAALGVIPYG------------------------------------------------------------------------
Tt    IREQKARKEAKDQVLKEIIPVEDLIKKPEEYIINAGPGKQKLVLIDYEKKEETKDD-----KNKGAGTTQTGPAG--------QT
Pp    LKEQKLRKENQEQ---EIVDIFEFIDEPNKYIVNGVAEKPQMVLIDYEAQEEAKQQEALQAQKQKINTIQQQPAKGKGMGNQQQA
Pt    IRELKIRKQ---QAIKEAWSIEEHIQDP-KYIINSKPGAQTMVLNDYEKKEEVAPT--------------------------KVA
Lb    KKSAVGRRT----------------------------------------------------------------------------
Ag    LNASHDALLP---------------------------------------------------------------------------
Da    TKLSSAEAAHG--------------------------------------------------------------------------

    1276
Hs    -------------------------------DDEEEEEEDKTVKYSNLPNYLLGSLSTDFGVDTSLLSSQLELHSREEKINQIIL
Gg    ---------------------------------------TEVMAGDTASCCLVGSLSSQYGADTSILYHQLDLHSREQKINQIVL
Xt    ---------------------------------------EESVSKSQDSSSLVGSLSDLYGGDTSYLYSQLILHSREEKINQIIL
Dr    -------------------------------------------VSLTESPALTGSLSSLYGGVNPYLYNQFNLHVREQKINQITL
Sr    -------------------------------------------DSKTESPALTGSLSALYGGVSPHLYSQFNLHIREQKINQITL
Ci    ------------------------------------QGGDEDEEEEKVGLSLHGSLGPNYSGG-EMFYSQFDLHTREEKITQIIL
Tb    -----------------------------------------------------------PSDVDELLYDTLDVYTGPRATIQLIL
Tviv  --------------------------------------------------------DIFLEDNTALLYDALDVYTHSRAIIQIIL
Vc    -----------FMFRGSAFESAGDGDDNYDGSTPTPDAAAAAAAAAVAAAAAASATGAAGSDPDTLMYSDFDLHCNTRRALQIHL
Cr    -------------------------------------------------------------------LIDFDVYCNTRKALQVQL
Tt    AGQGFGGRARHGAAQAKKEE----KKVEEVHHEEKEKKEVEEKEEEKKDED--------DLGEWDFLYGAFELFTNNRKRNQIII
Pp    HGHGHGMGRQRFNQQAKPKQQVDLKKVDKEKEEAEQKKKEQEKKQESQNQEQENNGESAELNEWKYLYGALELFTNNRKRNQIRL
Pt    AKPGVGGVQQNIKQRQQQQQ--------QQQQQEEEKRQEEEKQKELEKQQQ-----YEELTEWDYLYGVSELFTLNRKRNQMIL
Lb    ---------------------------------------VPGLQIMSKTATPADGSSSGTEDLDAAMLNDADVYTQSRMVLQSLL
Ag    ---------------WKPTPTYQLESILNRDPEYANELDNLARASLEKSYALSGTTTHQFFQPYALRYDQLEVVTFEQLYFELIY
Da    -----------KAIPPTKYPQEREPIEKASEKVLNLLKGDDNDEEEKFALSGTTTHRWIREDPSRVVNQILRDGSHCEGVKRNIL
```

```
     1361
Hs   LKDIIYKVKTVFNNEFDAAYKQKEFEIARVKERNVRIREIILDLELEEA-VWQPEFE-----------DCEKPER-TLVVQDE-E
Gg   LKDIIYKVKTAFNKEFDIVAQQKEQEIARIKERNLRIREILAQLDLQVE-VWEPGLT-----------CDENPEQ-VLTVQDS-E
Xt   LQDIIHNIKKAFNKDFDIACRQKEQEVTRVKERNNRIQEIMVELNLQEK-LLEPTFT-----------DNEKPER-ALTVDDS-E
Dr   LKDVIYNVKSAFNKEFEAVYKQKEQEIHRTKEKNKRIFQIMAKLGISET-LWEPRLT-----------DNECPER-ALTVTDS-E
Sr   LKDVIYKVKSTFNKEFEAVYKQKEQEIYRLREKNKRISEIMSELDLSET-LWEPSLT-----------DNECPER-ALTVTDS-E
Ci   VKDAIRRIKSSFNEAFTNIFRNKEQEITRTMERNVRIREIMKELDLDADSVKDPQMD-----------VDEKPEK-VFIVTDD-E
Tb   LECKILHEKKSFNIRFDTLRERKSRELNLIAERNGRCVRIMQQLGEHTCPPNVLFTP-------------------VFDIEED--
Tviv LTGQTRYLKKAFNSRFDALRERKRRELGLIEERNDRCKRILQQLGDTTCLSDILFTP-------------------VCDPNED--
Vc   LRQKIRNLKVTFNTDFNRVAAQKRSDCDRIADLNARMDETVKDLRKMGAGGFG-----------------RVRAT-VLSVRDE-E
Cr   LKQKMRDVQAGFNTDFNKVAAAKKADCDRIADLNARLDDTLKDLRKLGAGPPAGLLDERFSLSAQQDTRDNIAAT-VLMVREE-E
Tt   LQNIIFKVKEEFNKEFDKMMQQRQVQVDMIAEKNKRIQEILHELHKEEEIFEPN------------KNILENPER-VLEVDPS-E
Pp   LHNIIFSVKEEFNKEFEKCMAQRSKELDQIQDRNKKIEEIYLELHKEPEYQEIK------------QNILEQPEKNILEVDPEKE
Pt   INDVIFSMKRQFNAEFELIQKQRQQQLDNINERNKRIIEINGELKRDPQLQLMK------------KNILEDPEK-ILIVKPE-E
Lb   VKGRSLALKDAFNTRFTDLQDLKRQSMMQVEERTLRCITIGKQLGSLPAQLFTAVVD-----------PEENPNS-LFVVEDK-E
Ag   GDLEIEKLRTLFNEKFEQIKTLKHEEMELVLKRNRRARYVQQELVFLGNLMADRNKFEVTEIEDPKYAADERPDT-IIHTEDS-E
Da   GKIREEKLKKHFNKLFEAMHSRKSSEIKLARERIARIDHCTLELRQMFQVDCTVDHSWLKLEWQ----ESERPES-MVEVEDE--

     1446
Hs   ITAHKHIKPWHKAK-ELIVNHEKEHWLLIQDASTRLRALMDMMGGVLEVKKEDILRMVIPQPAFMA-------------------
Gg   IKVEKYLTPQEREKAEMLTRLEMER-HLASLDNERQRALNDMMGGVLEVKQEDILKIDIPPPSFIS-------------------
Xt   VKVERYLTPEQKAKAEKLAKEEEAKRLAEQEDNAKQRALDDMMGGVLEVKKEDILRMEVPQPGFIA-------------------
Dr   IKVEKYLTLEQKEKEAKLREEEERQRLAAKSENMRDQGLSVMMGGVLELKKEDVLRMEVPQPEFMS-------------------
Sr   IKVEKYLTPEQKEKEEQLREEDKQQRLAAKSDNMRDQALSVMMGGVLELRKEDVLRMEVPQPEFMS-------------------
Ci   VTVERVLSKEQQKEKEEQERVEQERKEAAKQDNKRERALTDMMGGVLEVKKEDALKQDIPTPDFMDE------------------
Tb   -PQTVFEVFDSEIDPELLKLAVKSDDGELVVSPSDEAALKTWMDGLEKVTEVLRVNVPIPPF-----------------------
Tviv -PTAIFEVFDSEIDPELLKLADKTDDNAYVMSPANEAALKTWMDGLEKAIEVLAVNVPLPPF-----------------------
Vc   VGVERYITPEERARKEAARRAEEEATKRSARDNAGERALRQMMGGTLAPRGG-GVDGDGNPF-----------------------
Cr   VGAERYVSPAERAKQEAARKADEDAAKRSAKDNAGERALRQMMGGTLAARGG-GHD-ESNPF-----------------------
Tt   IAFKKFLTREEREKIERDRLREEERQRALKQDDAGVRALKDMMGGTLEEKKENPLDEGLEMEEWMN-------------------
Pp   ISFKKFLTREDRKKIEEARLKEEERLKALLADDAGRRAVQQMMDGTIEEKKENLLDEEIKKEEWMD-------------------
Pt   IGFQQYETREMREKKEQERLKEEARLKALMADDSGVRAVKDMMGGTLEEKKETPLDEKLEVEEWMK-------------------
Lb   LSAEAQALIAPSMGATVVS-PVDEAALPLWMDGLEKEVVRLEVHVPLPDFADDTRDTFIPPE-----------------------
Ag   IPVAPYISPSVERLLELERQERERRQRELLEDDFKDRALVTMMDGVLEHRWEDEIKKSLPLPQCLVFEAIICGQEYRLTLFVVFC
Da   EVYRVINDSGSEANVKRLDNSVTREETSVLADTFRTGELKKMMDGVLEVKWEDEIKKDIPRPDCLMK------------------

     1531
Hs   -----KPDAVWTEEERKQFKDYEKKVKELNEERDKYRKSLEAELKKLQNSIQESTQAFDEHLKRLFERRVKAEMVTNQEELKISN
Gg   -----KPEDEWTAQEKKIFREYEEKVKELNEEKEKYRRTLKNKLEELEASIQEMTQNFDKTVCKLFERKVNLEKVIYQEELKIVN
Xt   -----RAEAEWTDEEKKQFKEYEKKCKDLSEEKEKYSKVLEAEMKKLQLSVIETTQSFDDVLARLFEKKVKSEMAIYQEELKINN
Dr   -----KPEAQWTEEERKSFKEFEKKAKELSEEQEKYRKTLETEMKKLLTSIKEATQMFDEKLAKLFERKVKSEMVIYQEELKIAN
Sr   -----KPEVQWTEEERKSFKEFEKKAKELSEEKEKYRKTLETEMKKLLTSIKEATQMFDEKLTKLFERKVKSEMVIYQEELKIAN
Ci   -----KLEKDWTDEEKKKAKEHQKKLKDLQEEREKYRKNLENELKKLQQNNLDAITNYDESLQQLFNRKVKTEMVVNQEELKILR
Tb   --------ADNSLEQYVPPE---ERSDEQQRIFEEYEKEVAEQTVLINEKKELLRGEVAALVKANMTSAKAIDDEIDVLRTDRML
Tviv --------ADETLDQYVSPD---ERTDEQQRLYEEYEKQVVEQTILVNEQKEALRQELVALKKANDASCALIDGEVVALGDERMK
Vc   ---------SLPPPPWLAAMGLDPDTVNPKMLSEEQARELKDWQAREKNLQEERARRVGMLELELRTAKAAVEDVVGRFDEALAG
Cr   ---------SLPKPAWLVALGVEPDAVNPKLITEEQNRELKEWQAKEKSLQEERAKRITVLEMELRTAKAAVEELT---------
Tt   -----KPLEEMNEEERVRFKEYEVKKQRLEEEKEKIRKNLENELKKLKSDVNEICQKFDEKLLLTFKRKLEFDYRVYEQELYIVK
Pp   -----LPEEELNDEQKIKLKEYEVNLQKQQEEKDKIRKNLESQLKKLKQEIVEICQKFDQKLLILFRRKLEFDYRIYEQELYIVK
Pt   -----KSPDDMTEEERMKLKEFEVRKQKLEEEKEKIRKNLEAELKKLNNEITDICQRFDDKLLILFRRKLEYDYRILEQELSIVR
Lb   ---------ERTEEQTRIIEAYAKRLKEENECVEAKREALRGEFKSLQEKNRETAAKLDEQLHQIRQLRLNTAEEVDEAELQLAL
Ag   LQEIGKEPQHYNETDIREVNEYEDQSRVLHQERLRYRKMLQDELHELAISLDEQIKRFNTAVAKLTLQKIVIEAAIRQEEMRILR
Da   ------DPSSCSADDLRIIKNYEEALRRLEGEREKYRKCLEKEIEELTESLKSSVTHFNREMELFYLKRLKVESAVLQETLMRLR

     1616
Hs   LAFSLLLDEELSSREKFLNNYLTRKQHEKSQ-----TSEAVRKSREDLDVCKEHYDNLLAEDKVMDRSFKKEFSE-IPGHQVDIL
Gg   LTYSLLLDEELDSREAGLRYFLVKKQKEKVK-----IARTVEVTKRKIEFYTDQYDNAIAEDKNLEYGFRKEFAH-VPASLLGEL
Xt   LLFSLLIEEEINTRVAHLAHILDKKRKQKNQ-----TAEIVKSFKTQVLAFRESYDNLVAEDKLLDRGFKKEFSD-ITSYQVDQL
Dr   LVLSIQTEEEILTREKQLSFKLEKARIKKNE-----IGEELKKHKETVDEFREAYDNTVAEDKLLDKGFRKEFYD-VPGHSIDQL
Sr   LVHSIQTEEEILSREKQMSLKLEKARIVKNE-----IGEELKKHKETVDEFREEYDNTVTEDKLLDKGFRKEFFD-VPGHIIDQL
Ci   MTQAIMVADELATHEDQLNKLLELKNKFKAS-----SSSRLEDIQMLVKDYEEINDSTIAEDKMLDRAFKRDFSD-VPLSISDQL
Tb   VAQLVDELELHQVN------------------------------ALCLFLLKKTIRNKFLGVKREEEDLLCRLRQ-LDSLYEYRL
Tviv TSQQVDALELQQVN------------------------------ALGRLFLQKAIYREYVSVLQEKAHVDCRLEQ-LKNLESHRQ
Vc   LAARRHR-----------------------------VAAAVAALEARTLALAAGLARCAKTSEAVEKQLLARLGA-AKEAHGRAA
Cr   -------------------------------------------------------------SEAVERGLLARLGA-AKEAHGRAA
Tt   LAQQILKQQNIIIQLDQYDVTINKLTDQVRKGQANREILHEFKQEVD--QNKNKLQDQYQNSLNVKPSYGVDPNK-IRNIWVYAF
Pp   LSLSMIEDKYLNIKLEEISELSKDLSDDPSNKLKAGIDEFYKQELARRQQLQREAQNYFKNERALGDLQGLDLGK-LGLIWTKVF
Pt   LALSIIISQQAQLRVSELEKLHDEMTAQLNQLQQMKNNLDQTRDLTQ--QQRKTLNDQIVNYFNRGQTAGMDANK-VKILWQYAF
Lb   LFQHR--------------------------------------------LCVSAAYRQHQSMAQKREALRDGRTE-AEFIVAQQK
Ag   ATLYNHARLVYDANVARLRAQIEQNADYTDQ-----LTEVLNEFQEKAADYRNTYDTLRTKDRLLDKQFKINFSDTAQSALVDQA
Da   ESQRHHLRLKGMEAFAHLELNELAPAQERTK----KLIDDSSALESNVNESRVRYDNLSKRDKLLEGKFRGEFTD-LKQPMVDHL
```

```
     1701
Hs   YKLFKRRPRISKQK----------THSETTSVVPFGELPGSGKLNKDAFAQLMKAMDELDNISNMPEG---------LDPLVWNH
Gg   VELYKCRPRTVMTE----------IYLATANRSKSS--SVSAEDYKDALSRLMKAMDELDNPKHMPNG---------LDPSIWEK
Xt   YKLYKRRPRVQRLR----------TQTDSAAPFGERPGSAKANKDS--IALLMKAMDELDTPENMPEG---------VELPVWER
Dr   YKLYKRRPRVQRMK----------TQTENN---PFKEG----AAPSDGLSLMMKAMEELDAPEHMLEG---------LDPAVWER
Sr   YKLYKRRPRVQRIK----------TQTENN---QFKEGALLGTAPSDGLSLMMKAMEELDAPENMPEG---------LDPFVWER
Ci   YKLFKRRPRQQKPR------------TLAERSNSLDPYGVSDFSAEVTIFSTQDGLAELDSEVNIPEG---------VDSALWER
Tb   KLYLASEARVQDCI----EEEKNMITDMRCLPPFTDPDWGERLNRRFTTWRSKYEDGLAKVPEPTRSGV--------VPIPLWEQ
Tviv SLLTSAEEDAQQRI----DDERSLISNMRHSPPFSDPDWGDRLYRRFTKWCTRYEGGVAKVPNPGQNGV--------VPLALFDK
Vc   ADLTERRTALTELESKHAAAAAEERQLDRNFKKEFVDADLHFNRLLHMYRARRSEQLVAAAMG-TPEG---------LDPGIWEK
Cr   SELSERRAALAELESRQAQLGADERLMDRNFKKEFAEADIHLNRLLQLYRARKPEQLASMPGGGKPEG---------LDAALWDR
Tt   EEQKKDERKKKEIE--DKIMNDPKYRNELQKLDPFIELNKKEIKKKLDGIFDSYVDDIREKITNMQMFE------KQNEEHGVKF
Pp   KDQK-SALEKKQQE--EKLLKDENYKSELVELDPLFDIKKNNIMKSQQNDFNNYKLDIQQKATSAQLFMGGYGESQEEQQRDLEI
Pt   EDQKTNERAKQEQD--EKLLKIQKYKDVLIQIDPLFENQKKIISAQLDEQFETYLFDIQEKASNLQGID---------FEHVRDQ
Lb   HLLRAAQARLAAAE--------SKTGGYAASVRALAPFEDGTTGEKLHRRFVRWQRRFVDGSAPLPDAD---TSAADCTAEQWAA
Ag   YKIFKRRPKTQLRS---IVTVSVFQDMAKRIVAKKTPGTHGNLLLPKECQDYLAHCESLDQPSNCPAG---------MDSSLWQT
Da   LRHYKKRPRLGQLT-----------CTSITYLTEMGKCILSGEKSEILPRECLDYSKGMDMLDVMPSG-----LPNQIDGCHWML

     1786
Hs   FCMTRRAKVENEQKVKQKAADLLEMATFLQKRVEEEEKVQQEIERVFHELILLQEEKVRFQLNLTIQILLKQGQVELEN-FQLVL
Gg   FCLARRNKLESEQLVKWMALILAEIEVFLQRRIDDNEKMSSKIENIFKELIWLQEEKIKLQVNLTFQFLLKQGQVEVES-TEIP-
Xt   FCLARRSKIEYEQQVKIKALVLAEMQAFLDKRIEEDEKIRQDTENIMQELNVLRSEKMKFQLDLTVQFLLKQGQVELEN-SDLIP
Dr   FCLARRAKVESEQKVKLKALTLAEMQAFLQRRIDEDDEAQLEIKNLIDELNGLCDEKMRFRLDSMVQIVLQQGQVEVEA-GEFIA
Sr   FCLARRAKVESEQKVKLKALTLAEMQAFLQRRTDEDEKAQLEIKNLIDELNSICEEKMRFRLDSMVQIVLQQGQVEVEA-GDFIA
Ci   LCNARRNKVESESNVKQSVTTLADMMKYLHHRQNEDEVLEHSIDDVIKQQTKLCEDSMKNMLNLEVQFLLKQGQVEINC-GPFIS
Tb   YCQCCRAVVEARDKIIHLRGEADALNDEVVEVETEKKKAQFALDDKEKAEEACRKEVIEKVLDIQNLYTLQQGQVQDEN-AMVSD
Tviv YRECCIAVVKAKEAIAHETERVHQYHECLVAVYVEKSRLEEILTRKKKAADACKNNVVQKLLNVQILHNLQQGQVQDER-AVVNE
Vc   FTLYRSSRLEAEAAVRATQVDLALARRDLPELESREAGLATEMDELMSAITALRTERRTSAYDNELQLRLLAGQVEVAPPVGAPA
Cr   FVAYRAERLAAEAGARAAAGDLVLARRDLPELESREAALGSEMEVLMGSITALRSERKVAAYDNEVQLRLLAGQVEAMPPRPASA
Tt   IKDVLQQRFQFKKELDQSEVEFRDINNSITNFENDLFALQQKLKEQKSEQRAHIQALKKGKSNIELIIRFKQGFVEIPS-DKPVP
Pp   LYNLMRKKFELEKEYEQAEANLNDIDACLTQKSKEASEVIQQENLYQRQIMEAKESLANSKSNIEVILRFKQGLVEINNQNLLTP
Pt   LLQVLSQRFRMKLDYEKIDKDSNDLENFNKKFEQEFNQLSSQLQNSEEDIQSLHNMLEKSRSNIEVMFRFKQGYVEISQ-DKPVP
Lb   FCDHCQAVAELQQLVNDAEAEVQRAAVDVREAQRHCKLITDEIDVTGEAMAAVRGSSVARLLDVHALCRLRQGQIQDEG-ATTAT
Ag   LIKMRRIKIESEFRLKSCELMLSDAEAAIAAFQREITSKRNVLTSLEQTLDQLQNDQFEAATNRTVQLVMKRGWIEIQQ-SGRTS
Da   LCKLRRSKVEIEIRLKSCAVELAEAEQTLTSHQKAIQTSQNRVQQLRETITERKRQNVEAMRDMKVQLVLKMGQIETQL-SGNPR

     1871
Hs   EYSDAILINKNIIEDLNSVIRTQGQKKVASMMESKDVHKRILQIEWEHKKMEMEREDLNQKAWDIQMLFFSRDRQKYLNEPNYEA
Gg   DYADAIFIKKSIIEELNCNIMDQVEKKIASMVECKEFSKGIFQLEWEHKKMRMQIEDLNQKAQDIVTLPVTKDRQLFLTMLDYDS
Xt   SFEDAILLHRSVIEDLNSTIRGLGEQKIASMVESKDFRKGIFQLEWEHRKIRMEMEDLEQKSRDISLLHVSKDFQVFLSEQNYDK
Dr   DYSNALLIQRKVVEDLNSTIRALGDQKIATMVECKDFRKGIIQQEWEHRRMRMQLEDLSNKARNIQTLRITQDIQDYLNETNNDN
Sr   DFTNALLIHRKVTEDLNSTIRALGDQKIASMVECKDFRKGIIQQEWDHRRMRMQLEDLSNKARDIQTLRITQEIQDYLNETSNDN
Ci   DYSDSILIHRGVVEDLNGTIRTLGESKVAAMTESKDFKKGIHQLEWERKRMVMQMEDLHNRARHIQMLKLTKDLQAFLNEENNAA
Tb   DFTDFSIRWVKNITDYNDLIFASFDEIRSLMSRSSQLRQSMKTCSWETERLLYCIGTLEMELRQLHTLRVTRQMQETIHTGA-VT
Tviv NFMNFSFRWVSSVTDYNDLIFQSFEEFRRLMVKRSQLRQAMKKCAWDTERLLYCIGTLEMELRQLHTLRVTRQMQESIHTGS-VI
Vc   DMSDARLLNQAVVEALNAVVLGKGTKKVEILTAMKDFKRGIYAAQWEAAAADMRLEDLRAKIRDLQLLHVTRDMQAVLKDGE-DR
Cr   DMSDARLLGRGVVESLNSVVLGKGTKKVELLTAMKDFKRGIYAAQWEAQAADMRLDDLRAKIRDLQLLHVTRDMQTVLKDGE-DR
Tt   SITDAILIPRDEIEKKNKRIDQEGKKKIRLMEDIKNIKFEVAEAEYDLKKRDLNEQKVRCSTREVQLLRVKKEMQVALTKKD-MR
Pp   SMEDAILITRQTIEQKNRSVQKKGDKKIRLMEEIKTNQDKVSEDNYNLKKLEKSIEKVENEIREIQLLKVKKEMQIGLQVKN-KK
Pt   NLQDAALIPRETIEKKNDEIKKEGDTKIELMKDIIKSKYQVEKNEYDLKKRDLIIQDLECKTREVQLLKVKKEMQIALTKDD-TR
Lb   AFLSSNLRWRDDVSQYNDLILQSEAESRALLIKAFAQQKLMRLLDWDTERLRYCTGTLQLELRQLHTLRVTRQMQEWLSGDA-EV
Ag   DFDRCVLIHRTDVEDINAIIRRAGTKKLNAMINAALFRRKIIFQEWEHRALKLQLRDLRDQLATVEKCKITKEVQSWLKMKGMKR
Da   DLDNAVLVPFDVVLGINDAIVGAGRKKLGAMKRTMEFRRTIEWREWRHACMKMTLEDMKEDLKILQGVKVTREIQRYLMRHDAVG

     1956
Hs   LISIQIGIMEQTIAVLDKMHKKNVENCKKLLKKLGKF-SNQKDIANYALSCNLREELVAVSERKDICNAMGSKLTCE--------
Gg   YMAHRIAVMEQTLAVMDKLHKKNMKKQQKRIKELEKC-ISLKEQANYELSLELKETLVSVSERRHISQAADMQHISE--------
Xt   RISDQIQVLEATLNAQEKQHEKNVKTYKKSLKDLETN-INKKRTANLDLDKDLQELLVSFSERKHIYDVVGVEQSTE--------
Dr   RVSKQLTTLERTLDLQKTAHQKKIEICKKQKRQLDRQ-VAKMQEKNAALDLRVAEMEITVAERRNFYEAAAMEDNQE--------
Sr   RVSKQLTTLETTLDLQKTTHQKKVEACKKQIKHLDRQ-GVQMQEKSAALDLKVADMEITVAERRNIYEAVAMGDNQE--------
Ci   KQSQQVAVLEQTLALQDKQLQKNISHKSKLHRDLERT-IRLKEQENHQLDRVLEELHVSVSERKNIADVNAGER-AK--------
Tb   SLEREINKMDARIEAVRSVMSKKVEERNRVISKLKMQ-INDRRAENQYLNNQVQALTNSVEDKKAVWGMLGEHNNDK--------
Tviv SSGEEVEKLKNRIEAVRSVMTKRVEERDRVIARLKMQ-IQDRHIENSFLERRAQQIKSVVSDEKAVWDMLSGHNDEK--------
Vc   STALEAASLEALMKQRERLHIKALDDKRRRLAKLAGE-VSSRSGQNQEVAIHLVTLGKVLEEQ---QRLQAGMQSST--------
Cr   SSALEAANLEALMKQRERLHAKALEDKRRRLRKLAAD-VSSRSAQNQEVAVHLVTLGKVLEEQ---QRLQAGMQSAN--------
Tt   LNESALKNLQEQIEKVKQATDKLVNIYEKKCKKIDEQ-IEFIRRENEQLRAQGVSLRNTVQQEEKFSATMFKKNQQQ---GADDK
Pp   LQEEKLQDLSNQIKILVETAEKRIKDLKKKEDKIQEQ-IYLIEKENDQLISQQEVLKNTVQHREKL-VDLYKKNTNQNPGGNKDN
Pt   LNERELANLKDQIDLLKSATDKRLQIINKKREKIEKD-IDFIKKENQQLIGQGGVLSGNVKQLQDISDMQNKKNVRQ-----GEE
Lb   SEEKMLAHLQRHMDMVEMNMSRKIEELRSVGRRLKSQ-IAERVTENTIVDAQCDDLDDTVRANTAVYRLMETGADSL--------
Ag   SEDFSQMALEKKIRNAVQNEEEMLQELKTALEEIEQR-IATKRKDNKQLDQQTRTLNIDVTEQHLQRDAELEQTEQK--------
Da   GVDKDTHITEKSILSKKKQRLSTILDTEKSRLFDIIKTVNQWQKRNDRLEDKIEKVKTENCKMSLLANDEARVRQER--------
```

```
     2041
Hs    --KIVKERYENMMQQQKLTNISKQQAEQISILQTEVERLRMKTFPALVQM-----------------------------------
Gg    --RIAKRRYQEILKQKHLRGLIEEQEKHLDILQAEAERLRSKTFPTL--------------------------------------
Xt    --KAAKERYVEIVQRRKLVDLAKIQAQEIIELRTEVDRLRMKTFPALVQMEY---------------------------------
Dr    --TESDKNYQDILLRKKLLAIARAQSEELLLLRAELEKLRMKNFPSLSQLHYN--------------------------------
Sr    --TEAEKNYQDIILRKKLLNIAGAQSEEILTLQAEVEKLRMKNFPSLSQLNYN--------------------------------
Ci    --NTADKRLKGIVQRRKLVDLAKAQAQEVAVLRAEVERLRMKTFPALVQVER---------------------------------
Tb    --DRLRERMRELYENSELEELARCQQEELVRLKNEVDRLREATFPSFAVVTRRTAR-----------------------------
Tviv  --DRHSERMKELFEHSELGELARCQQEELIRLKKEIELLRERTFPSFAVVSKRTVRQ----------------------------
Vc    --EQAARRMRSLVTHKKLKEIALAQQNELADLRGQVEKARLRTYPTFVELRNVAGMPTLPQRLPPDLKLAMRARSEADRACGDSN
Cr    --DQATRRMRSLVTHKKLKEIALAQQNELGELRQQLEKLRLRTYPTFIESGAVAGMPSPPRRLPPDIKLLAGSPSSSSVAGRT--
Tt    TDFEAYDKFKQIMNNRQLFTRVKRQTEEIELLREELGKLKARTFANFTAVQKY--------------------------------
Pp    DADESRKKFEAIARNRKLYDLGRRQIETIEAFRDELNKLKAKTFANFSVVNK---------------------------------
Pt    QQEPQEDKFTQIARNYRLFKKAKEQAEEIEILRDELTKLKAKTFANFQQVHR---------------------------------
Lb    --HARAARAKEIFCTSELEELARSQQEELVRLKHEVDRLRERTFPSFAVVSKQTR------------------------------
Ag    ---ATQDRMTAIVERARLVRLVQAQHTHILELGTMLELQRLKTYPTLTASTSVMTHNVHHLLGN---------------------
Da    ---FVRAKLKAIMKRNRLVKKIKDNYAELTSLRTQLELLRLKTYPTLRLKDARHLRKN---------------------------

     2126
Hs    --------------------------------
Gg    --------------------------------
Xt    --------------------------------
Dr    --------------------------------
Sr    --------------------------------
Ci    --------------------------------
Tb    --------------------------------
Tviv  --------------------------------
Vc    VLCFDKICTPSHVPYLSDDVYATAMRPTPPGQ
Cr    --------------------------------
Tt    --------------------------------
Pp    --------------------------------
Pt    --------------------------------
Lb    --------------------------------
Ag    --------------------------------
Da    --------------------------------
```
