# Supplementary figures and images for: Ciliary proteins Fap43 and Fap44 interact with each other and are essential for proper cilia and flagella beating
Source: Cell Mol Life Sci. 2018 Apr 23;75(24):4479–93. doi: 10.1007/s00018-018-2819-7 (PMC6208767; doi:10.1007/s00018-018-2819-7)

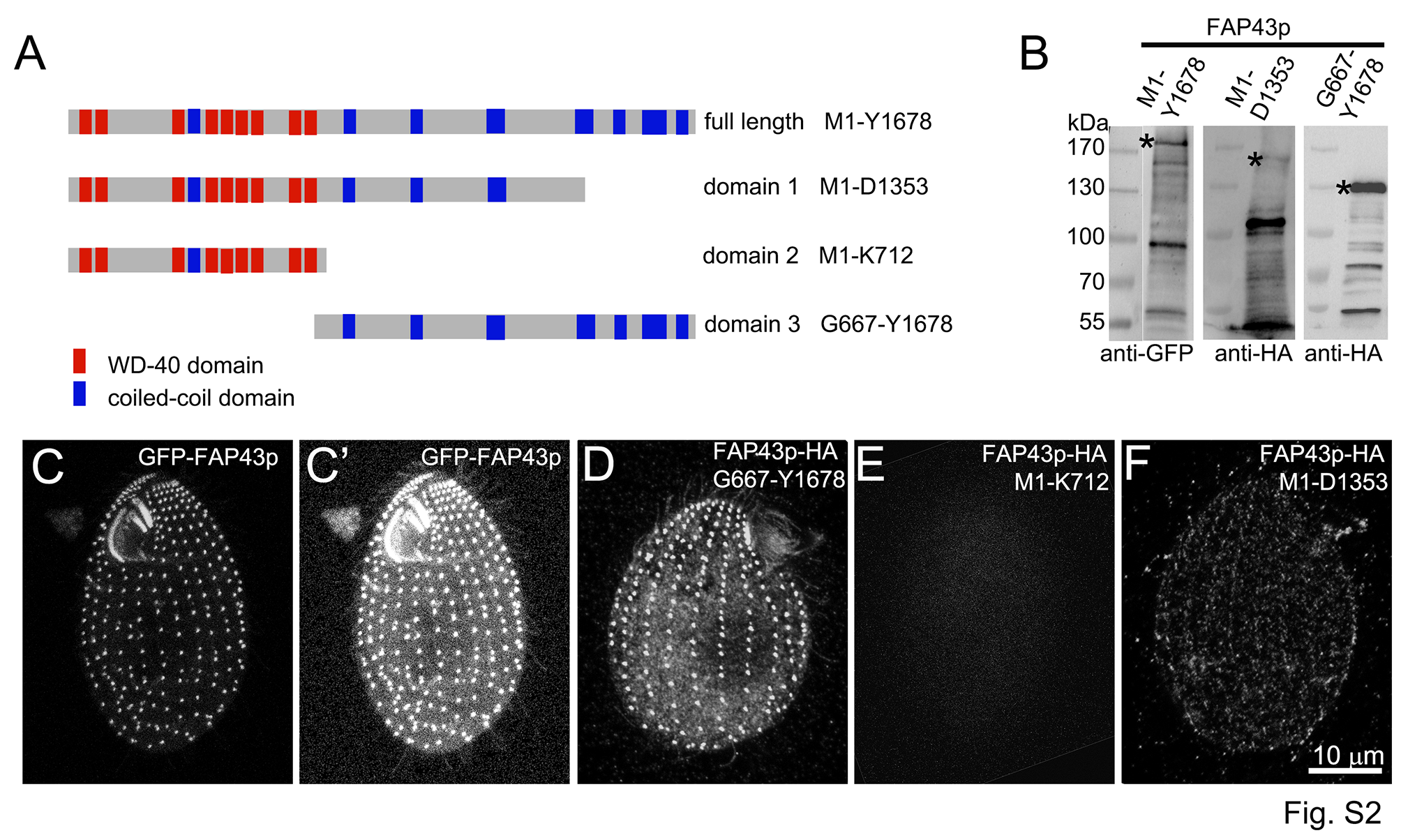

Supplement: Supplementary file 2 — Fig. S2: A C-terminal coiled-coil-domain-containing fragment of Fap43p is sufficient for targeting to cilia. (A) Schematic representation of the motifs and domains identified in full-length Fap43p and its truncated versions. Red rectangles represent WD40 repeats as predicted by the SMART and WDSP programs; blue rectangles represent coiled-coil domains as predicted by the SMART and COILS programs. (B) Western blot analysis of the cytoskeletal proteins isolated from cells overexpressing either GFP-tagged full-length Fap43p or HA-tagged truncated versions. The numbers represent the molecular weight size marker and refer to all blots. The stars mark the positions of the detected bands corresponding to the predicted molecular mass of the overexpressed proteins (GFP-Fap43p M1-Y1678 = 224 kDa, Fap43p-HA M1-D1353 = 159 kDa, Fap43p-HA G667-Y1678 = 121 kDa). The majority of the Fap43p-HA M1-D1353 fragment is degraded (although prepared under the same conditions as the other samples). Fap43p-HA M1-K712 was undetectable on the western blot. (C-F) Immunofluorescence confocal images of cells overexpressing either full-length GFP-tagged Fap43p (C, C’) or truncated versions of the protein (D-F) containing coiled-coil domains (D), WD40 repeats (E) or WD40 repeats and 3 out of 7 coiled-coils (F). Note that the Fap43p M1-K712 fragment is hardly detectable. C’ – overexposed cell presented in image C to visualize GFP-Fap43p in cilia. (TIFF 1511 kb) [file 18_2018_2819_MOESM2_ESM.tif]

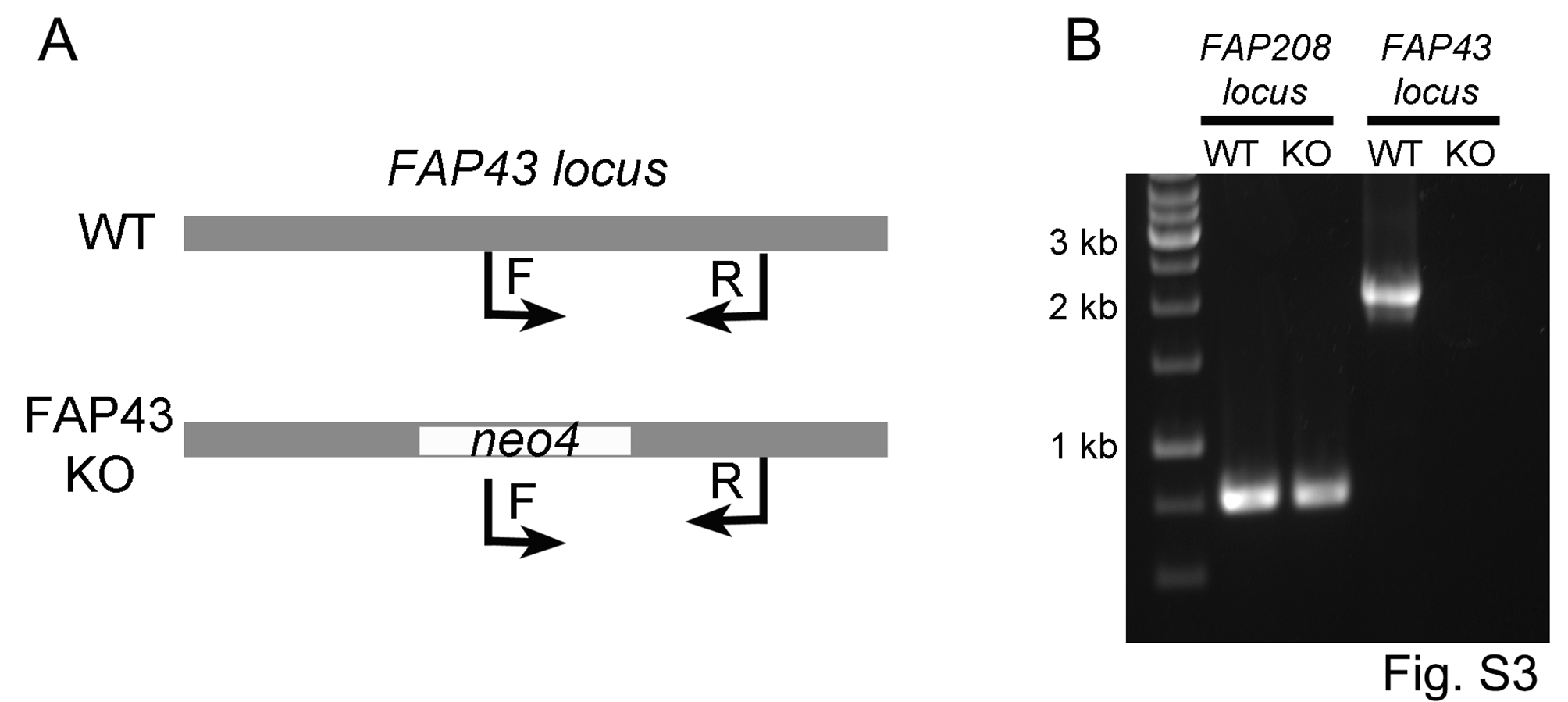

Supplement: Supplementary file 3 — Fig. S3: PCR analysis of the FAP43 locus in wild-type and FAP43 knockout cells. (A) A schematic representation of the FAP43 locus in wild-type and FAP43 knockout cells. The white rectangle represents a fragment of the FAP43 ORF replaced by the neo4 cassette. Annealing (if it occurs) of the primers to the FAP43 locus is indicated by arrows. (B) PCR analysis of the FAP43 locus with primers indicated in scheme (A) demonstrates that a fragment of the ORF was removed from the FAP43 locus. Amplification of the FAP208 locus was used as a control for the quality of the isolated genomic DNA. (TIFF 315 kb) [file 18_2018_2819_MOESM3_ESM.tif]

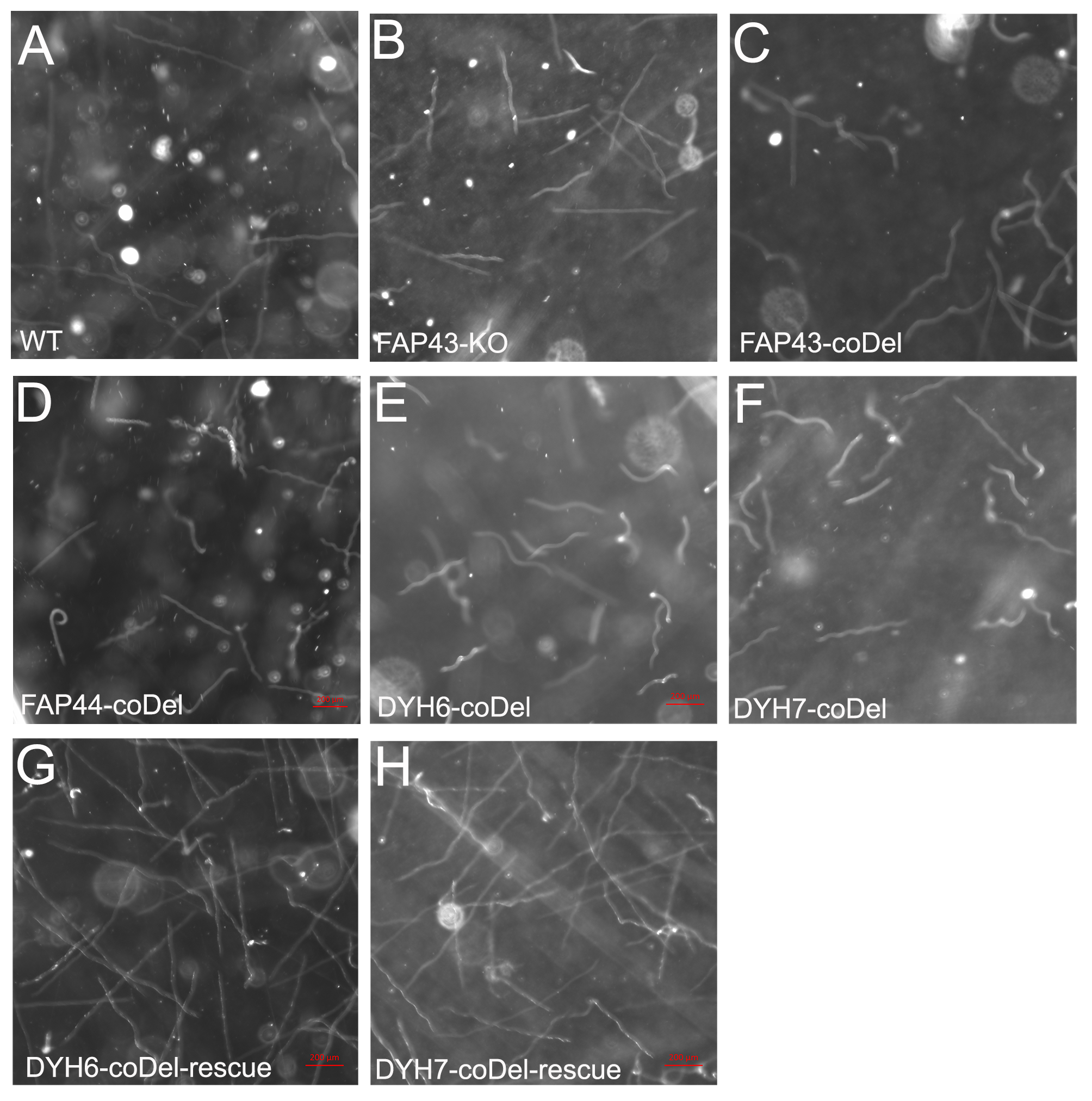

Supplement: Supplementary file 4 — Fig. S4: Comparison of the motility of wild-type Tetrahymena and studied mutants. (A-H) Swimming paths of (A) wild-type, (B) FAP43-KO, (C) FAP43-coDel, (D) FAP44coDel, (E) DYH6coDel, (F) DYH7coDel, (G) rescued DYH6coDel and (H) rescued DYH7coDel cells recorded for 3.2 s using a video camera. Red bar = 200 μm. Graph representing distance swum in 3.2 s is shown in Fig. 2a. (TIFF 2106 kb) [file 18_2018_2819_MOESM4_ESM.tif]

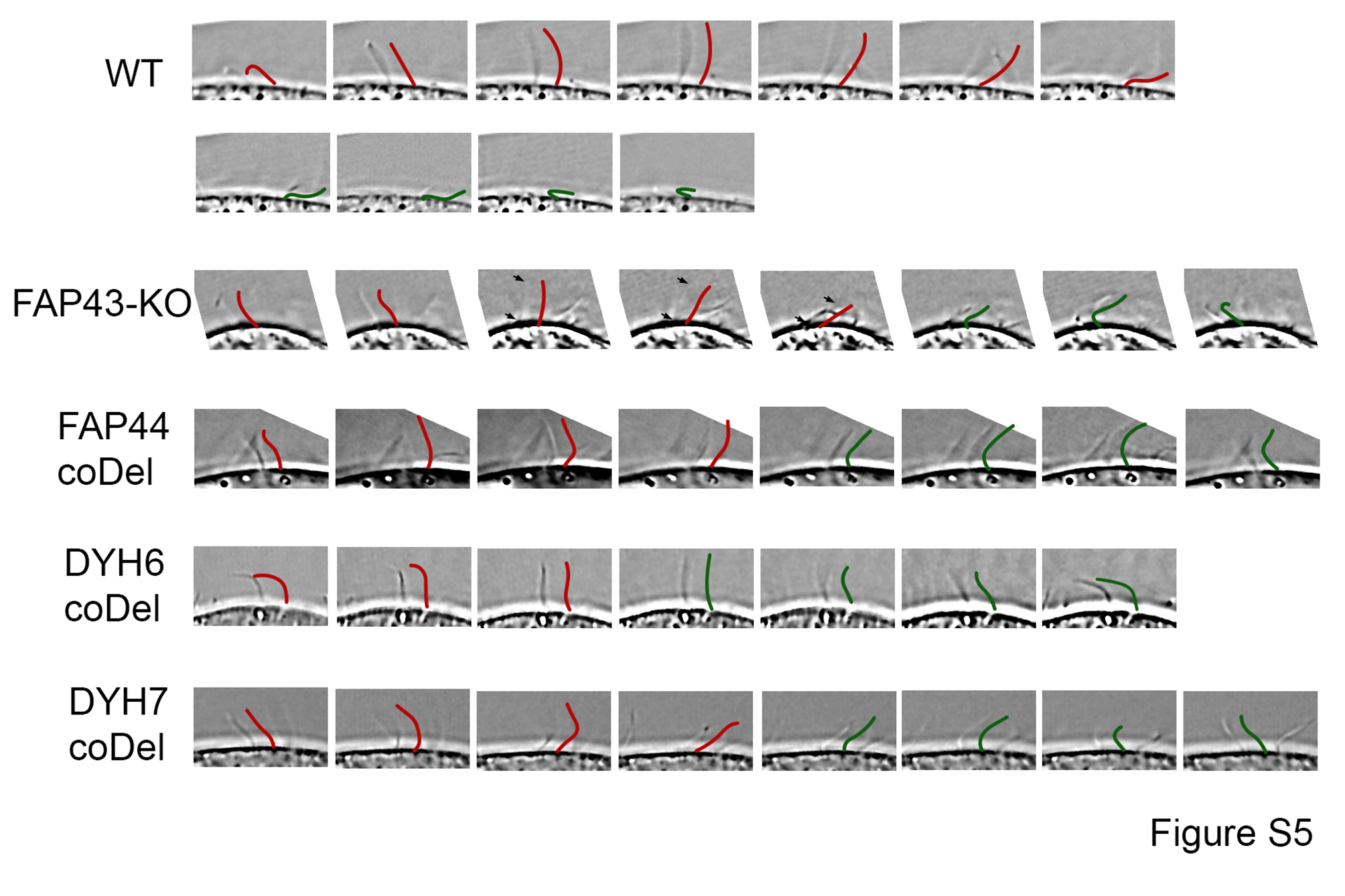

Supplement: Supplementary file 5 — Fig. S5. Tracing of the power (red) and recovery (green) stroke of a single cilium. Selected frames of the Supplementary Movies with marked cilium position. Note that the color line corresponding to the cilium is shifted to the side of the cilium. Red – power stroke, green – recovery stroke. (TIFF 7898 kb) [file 18_2018_2819_MOESM5_ESM.tif]

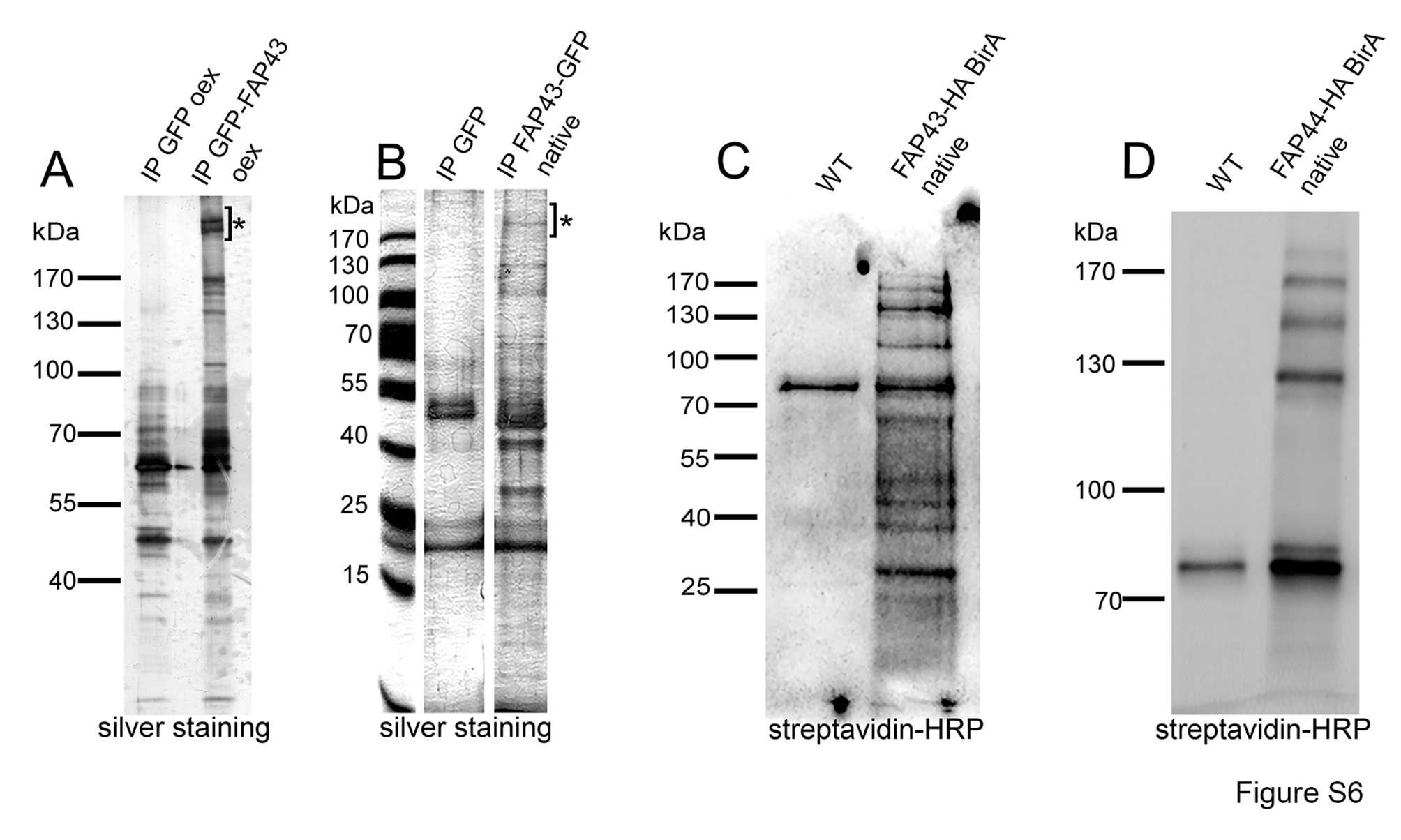

Supplement: Supplementary file 6 — Fig. S6: Fap43p is positioned in close proximity to Fap44p and co-immunoprecipitates with Fap44p. (A, B) Silver-stained gels showing proteins immunoprecipitated using GFP-Trap resin, either from (A) a cytoskeletal fraction of cells overexpressing GFP-Fap43p (GFP overexpressing cells as a control) or from (B) isolated cilia from cells expressing either Fap43p-GFP at the native level or GFP under the control of an uninduced MTT1 promoter (control). Bands marked by brackets and stars most likely represent GFP-tagged Fap43p (224 kDa) and Fap44p (241 kDa). (C, D) Western blot analysis of the biotinylated proteins in either wild-type cells (C and D, lines to the left) or cells expressing Fap43p-HA-BirA* or Fap44p-HA-BirA*(C, D, lines to the right, respectively) under the control of their native promoters. Note that only one major band of biotinylated protein appears in wild-type cells. Predicted molecular weights of the BirA* tagged proteins: Fap43p, 233 kDa; Fap44p, 250 kDa; Fap57Ap, 187 kDa. (TIFF 897 kb) [file 18_2018_2819_MOESM6_ESM.tif]

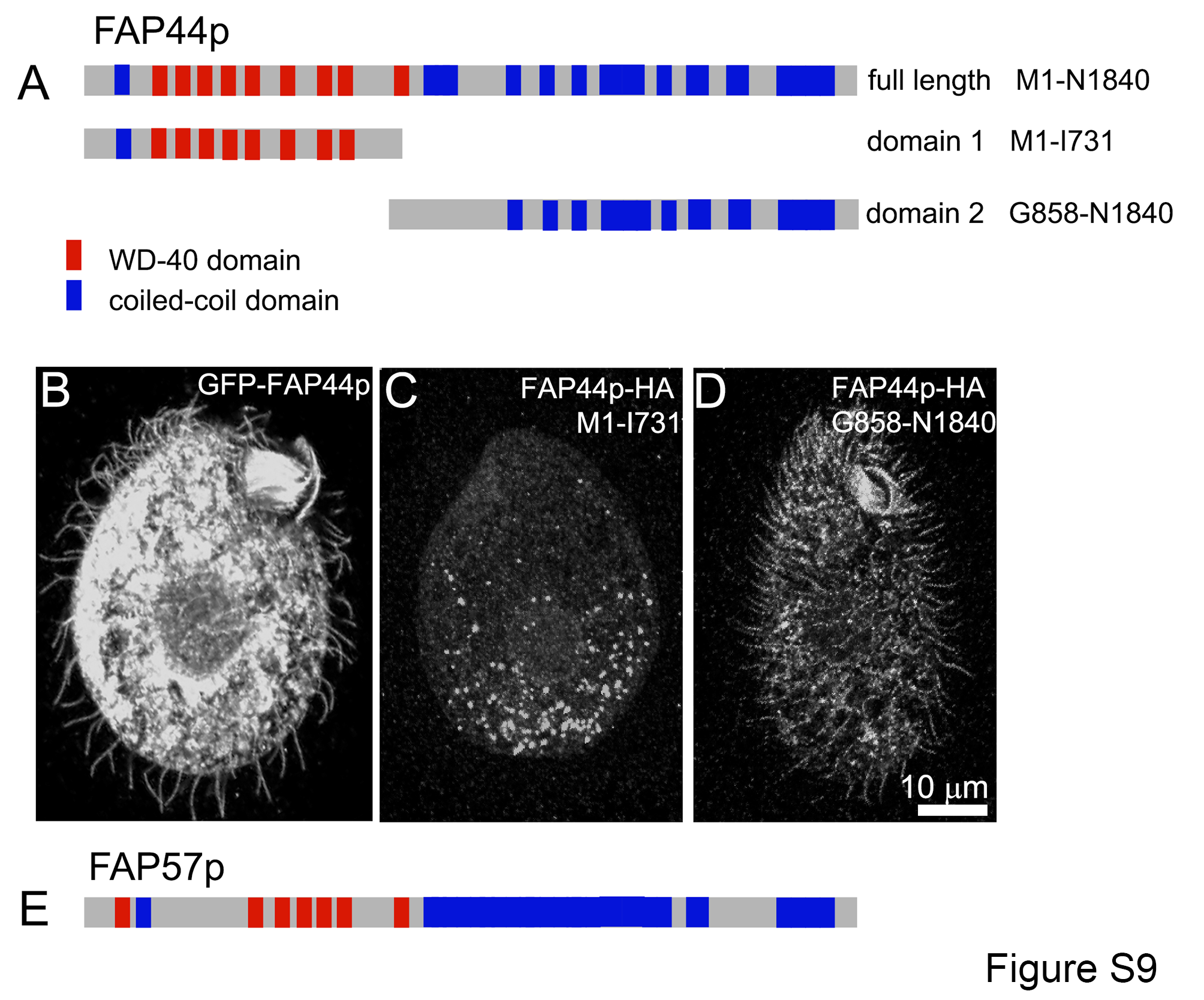

Supplement: Supplementary file 9 — Fig. S9: A C-terminal coiled-coil-domain-containing fragment of Fap44p is sufficient and indispensable for cilia targeting. (A) Schematic representation of the motifs and domains identified in a full-length Fap44p and its truncated versions. The red rectangles represent WD40 repeats as predicted by the SMART and WDSP programs; the blue rectangles represent coiled-coils as predicted by the SMART and COILS programs. (B-D) Immunofluorescence confocal images of cells overexpressing either full-length GFP-tagged Fap44p (B) or HA-tagged truncated versions of this protein (C, D) containing either WD40 repeats (C) or coiled-coils (D). Note that the Fap44p M1-I731 fragment forms cytoplasmic aggregates and is not detected in cilia. (E) Schematic representation of the domains identified in full-length Fap57Ap. (TIFF 1478 kb) [file 18_2018_2819_MOESM9_ESM.tif]

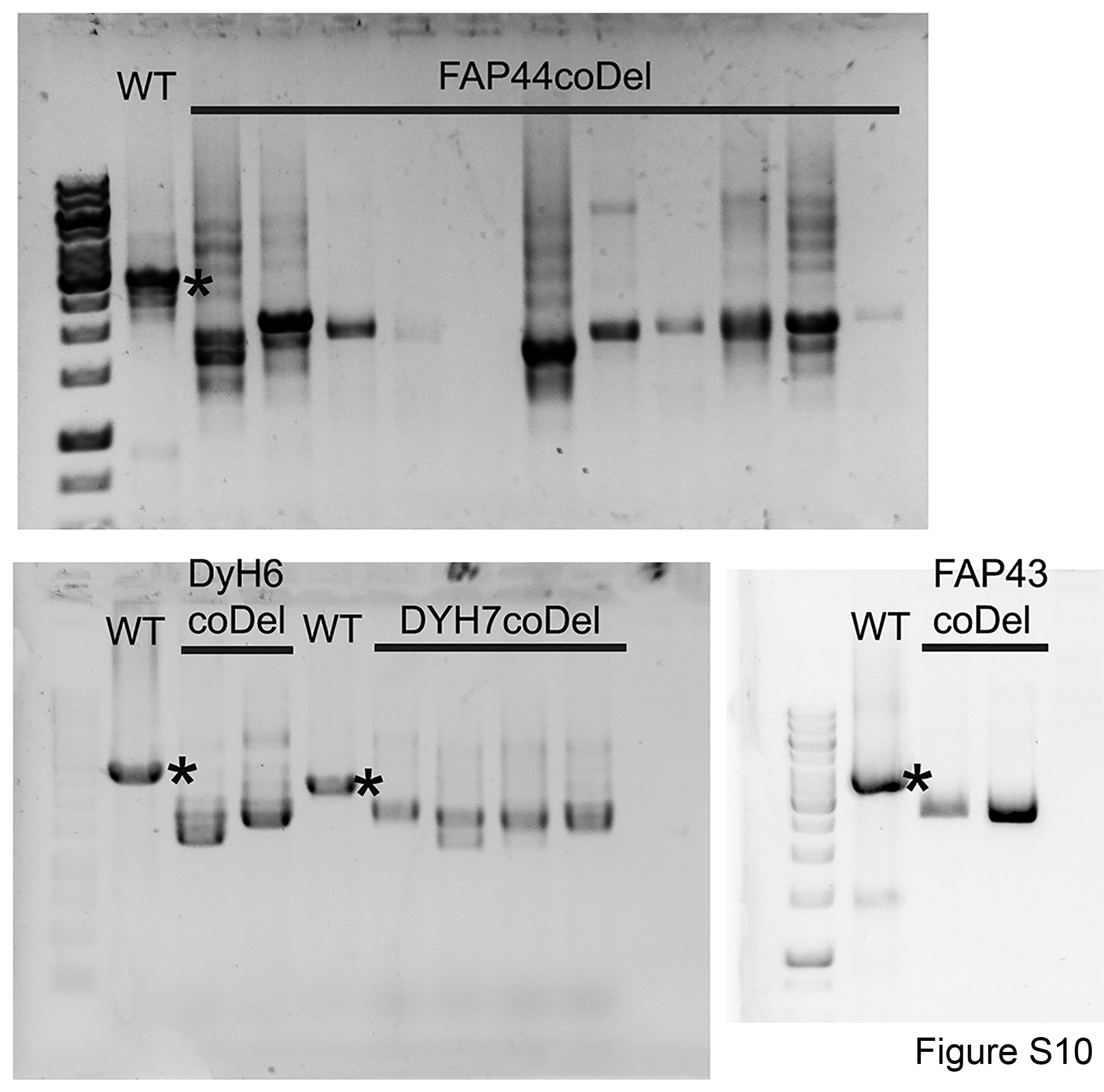

Supplement: Supplementary file 10 — Fig. S10: PCR analysis of the coDel mutants. PCR analyses of the endogenous FAP44, DYH6, DYH7 and FAP43 loci using specific forward and reverse primers annealing approximately 1 kb upstream and 1 kb downstream, respectively, of the amplified gene fragments cloned into the pMcoDel plasmid. Genomic DNA was isolated from cells of slow-swimming clones and wild-type clones (WT, control). Note that the fragment amplified using wild-type genomic DNA as a template is significantly larger (marked by a star) than the corresponding fragments amplified using genomic DNA isolated from mutants, indicating deletion of a fragment of the targeted gene. (TIFF 7811 kb) [file 18_2018_2819_MOESM10_ESM.tif]

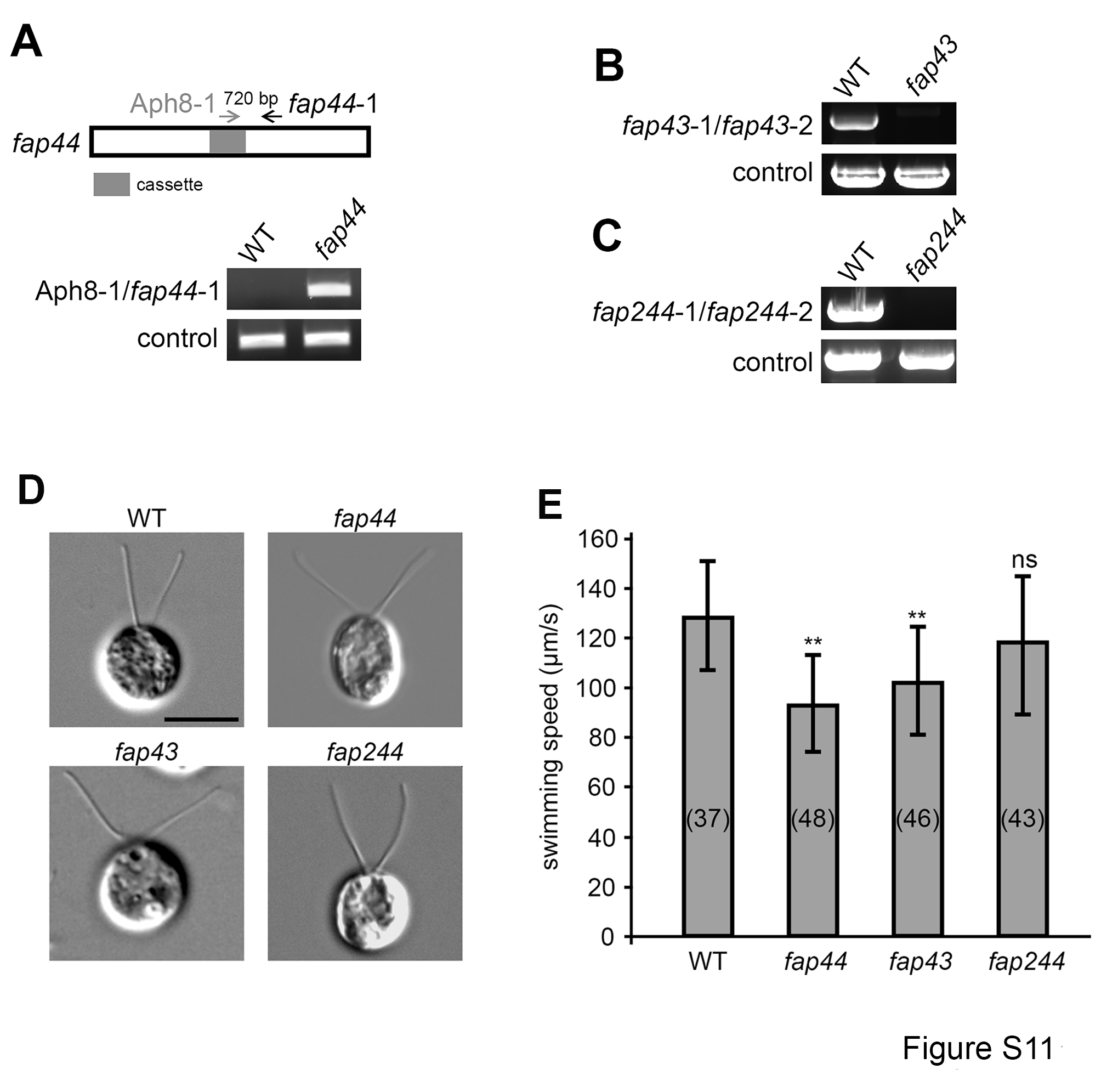

Supplement: Supplementary file 11 — Fig. S11. Analyses of the Chlamydomonas fap44, fap43 and fap244 mutants. Mutation and (partial) gene loss of the Chlamydomonas Library Project (CLiP) strains fap44 (A), fap43 (B) and fap244 (C) were verified by PCR. (A) A schematic drawing of the interrupted fap44 gene with the indicated position of the fap44 gene-specific primer (fap44-1) and an insertion cassette primer (Aph8-1) used to amplify a DNA fragment of 720 bp. Below, the amplification of the DNA fragment in the fap44 mutant verifies disruption of the fap44 gene by the insertion cassette. (B, C) The amplification of the DNA fragment in fap43 (B) and fap244 (C) mutants with a pair of gene-specific primers shows deletion in the respective fap43 and fap244 genes in mutant cells. Control band represents: (A) 5′ UTR region of the fap44 gene, (B) coding region of the rib43 gene and (C) coding region of the fap45 gene. (D, E) Chlamydomonas fap43 and fap44 mutants have normal flagella length but reduced swimming speed. (D) DIC images of Chlamydomonas WT and fap44, fap43 and fap244 mutant cells showing normal cilia length for all strains. (E) Swimming speed analysis showing that the Chlamydomonas mutants have reduced swimming speed compared to WT. ** indicates a significant difference (P < 0.01) between WT and fap44 and between WT and fap43 mutants. ns indicates no significant difference between WT and fap244. Error bars indicate ± SD. The number of cells analyzed is indicated on the bar graphs. Scale bar: 10 µm. (TIFF 11744 kb) [file 18_2018_2819_MOESM11_ESM.tif]
