# Supplementary material for: Ciliary proteins Fap43 and Fap44 interact with each other and are essential for proper cilia and flagella beating
Source: Cell Mol Life Sci. 2018 Apr 23;75(24):4479–93. doi: 10.1007/s00018-018-2819-7 (PMC6208767; doi:10.1007/s00018-018-2819-7)
Supplement: Supplementary file 7 — Fig. S7: Multiple alignment of Fap44p homolog sequences: Cephus cinctus (Cc, XP_015588735.1) Chlamydomonas reinhardtii (Cr, XP_001695270.1), Diachasma alloeum (Da, XP_015121875.1), Danio rerio (Dr, XP_017209920.2), Gallus gallus (Gg, XP_015152018.1), Homo sapiens (Hs, EAW79642.1), Ichthyophthirius multifiliis (Im, XP_004031186.1), Leishmania mexicana (Lb, XP_003873518.1), Paramecium tetraurelia (Pt, XP_001432360.1), Rattus norvegicus (Rn, XP_008767007.1), Tetrahymena thermophila (Tt, XP_001028010.2, TTHERM_00498220), Tribolium castaneum (Tc, XP_008191675.1), Trypanosoma brucei brucei (Tb, XP_845979.1), Volvox carteri f. nagariensis (Vc, XP_002947240.1) (PDF 105 kb) [file 18_2018_2819_MOESM7_ESM.pdf]

FAP44

```
     1
Tt   MSDQGLNDQQQEEFQNQPEVIENNQDEGQSQAQNEVDQNQQQESFKLENPADPNFPVEDATTLNYKKQVQEQENLAQSEEQSQQKSN
Im   -MSEEQQPIEENLSPNKLVEEQKEEVLQEEQTIETVNEQTEQYKEIIEIKQHEELHIQQEITIQTINNTTQQITEQYKEVVQKRKQE
Pt   ---------------------MDEDQKDQNSEIQMKRADSQAGVLEMQPPQIGVEQTSQQVPEADQEDDDQVVQTQVVQGDQQVVQK
Hs   ------------------------------------MKEPDDQDTDGEKSVTSKSDGKKSLRSSKSESRSPVQEDNTFLEDDTDETF
Rn   -----------------------------------------------MGKIRVKELREAFPESDSERNKWSLLCIHEAAEDITDDLF
Dr   ---------------------------------------------------------------------------------------
Gg   ----------------------------------------------------------MSSPNEQEGADEKPAEGTPADHSSVQESE
Cc   ------------------------------------------------------------------------------------MDD
Da   -------------------------------------------------------------------------------------ME
Tc   ---------------------------------------------------------------------------------------
Cr   ---------------------------------------------------------------------------------------
Vc   -----MLVSTAQLPALSTPDLKDTGSLVEGMAVDVRVLQDYRAPEDGPEGTDPTGASSSRATDVSPEADNVRPQDDIVAADPASTVT
Tb   ---------------------------------------------------------------------------------------
Lm   ---------------------------------------------------------------------------------------

    88
Tt   IKGSASSQMEQSVAGQNLPDLNILGNASIIKQQQQENQQEIDEMQEVEQEIPKIPDDFFYEENHVETQIADSE---ISKNAAQFYEC
Im   DEYEESLVEEEHIIEQEKNHQEVEQDNTFQNEEYAKNKVENQETPKEPGSQDHLTDQFFYDENELESTVTDSQ---ITRQTVQYHKV
Pt   SGAQNGMNSQSNIHQSAIINKHVQDMLDQSVYQVPKNDEVDPPKEDAPNPILDLIPEDYYPPDDWQPENQDQTPPLIHPDTIQFHDC
Hs   TKGEGSYLEEDSDEERLEGSLSSFQYGDLQSTTVPQQTPAPAVEEAEEEVKKKISESFFYDYMELASMPFVTLDSNIPLDLLTLVHS
Rn   TDGAESFLDDDLDLEDMNESSHSSQ--EYFHTPVVSQEPPPELKEEPEEVKKKLSETFYYDYLELISTPYVSSPDRIPLYFLALNHS
Dr   -----------------MDASETQTDEPSVKGVVFDEDDDDAAAGGADEEHRLIPDQHYYSYEELRSRASVTEHSHLPQDLLQLSHV
Gg   TGLPKESPAGGEQEEDVEQVETDTESIADDLEEQDNTQVSLKEGKSPEKEEKKISETFFYSYEDIYSRPFVTEDSGIPINLLTLKYS
Cc   TDEDIENVENLPEDPESNIRVSETSAIATRSEIFTTETSTEFQDEIESHEDKQDIPPIHYDSQDYISGPQRTENGTVPMNILEFHHS
Da   VDQLKPTNGLFPEDDDD------------------------------GENRSEFQSQVQYDKNNYISGPERVENGSVPMNILTFDHS
Tc   MSQTDEGLEDLPEDEEE----------------------------YEEEEEEEEDHWSIYDASTFISGPYQSEKCTINEGVLKFDFS
Cr   ------------------------------------------------------------------MPLMVLLLSQRTGGVAQLLSV
Vc   AADELPPPSDSMAGPSTLATDFGGRAPAAPSVGPAEALEFEGESEAVGVKEAEEGQELPVDGIEPVAADPAEELRAPVDSRAQILAV
Tb   ---------------------------------------------------------MAAPAQAVVKEKPPIHVYPQAKIGTKIFTH
Lm   -------------------------------------------------------MASPAAAGAENRPKPLHTYPEDVPPSSKILAH

   175
Tt   FGFDTAKKYNVSMLDNDNLIYSAGITYQIFNIVTREIRPYFSKDQGGIGSIAVNPN--KTHFAVAEKGD------HPNIYIYEYPSF
Im   FGFDSMKKYNVSVLDNENIIYSCGVTYQILNINTLQTQVLFSKDGGGIGSIAVHPS--KKYFAVAEKGN------KPNIYIYEYPTL
Pt   FGMDSNKKSNLWLIDSQTFVYASSISYHFYNFVQGTNEIFFSRDGGGIGSVAVHPS--RKYYSVAEKGS------FPNVYIYDA-SH
Hs   FGYDCRKRANLQLLDDSIAIYIAGNQLIFLNLKTKEQIYLRSSSGEGIGVIGVHPH--KTYFTVAEKGS------FPDIIIYEYPSL
Rn   FGYDCKRRANLQLLDNNTLMYIAGNQMVLLDFKNKTQMYLQSSSGQGIGAIGVHPD--KTYFAVAEKGS------FPKIIIYEYPSL
Dr   FGYECGRRANLQLLDEHTLLYIAGNLLVLLDVNTRGQRYIRSCSGCGIGAIMTHPS--RQYFAVAEKGR------QPNIIIYEYPSL
Gg   FGYDCIRSVNLMVMDSQSLLYVAGNQVVILDLKTKSQSYLRSSSGGGIGFIVAHPT--RQYFAVGEKGE------EPNLIIYDYPSL
Cc   YAYDCQKHFNLCVADPDTIIFASGNLINFFNFSENKLWFRRGSTGGGIGHITKNPT--FNHIAVGENGI------NPPIIIYEWPNL
Da   YGYDCRRHFNLCVPDPNTLIFSSGAFINFFDVENSKLTFQRCSTGLGIGHIAKNPK--EFHIAVGETGD------KPPIIIYKWPSM
Tc   FGYNCRKYFNLTAPDPDTLIFASGNFINFFDVLTKTISFRRSALGGGIAHIKANPNPEFNHVAIAENGK------IPLIIIYEWPSM
Cr   HGLDTHRRNNLVLLDEDTAASCIAGQLVLLSLSTGARRYLPGRDGGGVGAVAVHPS--RTLLAVGEKARPGPASAGPAVYIYSYPGL
Vc   LGMDTHRHNNALLLDEDTLVTCIGSHVVFVSLNTSHRRYLRGLDGGGVGAVAVHPS--RTLLAIGEKAR---GSTGPCVYIYAYPEL
Tb   HGIHSMRYNGVCALTGSTVLMASGRFVMFVDVHKGTIESMQGPENGGVGAVAVHPS--RQYYVVCERKP-----SDPAIRAYSWPSR
Lm   CGFDSRHFNAVAYLGDNTLLTSGGKFVLFVHVDSGAVESQDGPLDGAVGAVAVHPG--GTSYVVGERCP-----ANPLLRAYGWPSR

   262
Tt   KLYR------------------ILRKGTELSYSSVCFSNN-GEIIAAVGSYPDYTISLWNWKQEVIILKAKAFSQEVYSVNFSNYSE
Im   KLFR------------------ILRKGTELSYSSVTFSFD-GIRCASVGSSPDYTISIWDWKKEMIILKAKAFSQEVYHVQFSEYSE
Pt   KLYR------------------ILMKGTERSYSNSNFSKD-GDKFATVGSSPDYQICIWDWRQEKILLKAKAFSQEVYKVGFSEYNV
Hs   RPYR------------------VLRDGTEKGYAYVDFNYS-GNLLASVGSNPDYTLTIWNWKEEQPILRTKAFSQEVFKVTFNPDKE
Rn   KPYR------------------ILQDGTEKAYAYVDFNNE-GNLLASVGCHPDYTITIWDWKGEQPILRTKAFSQDVFKVTFNPDND
Dr   RPYR------------------ILRGGTGVAYSCVCFSAD-GSLLASVGSAPDYTLTLWDWRSERATLRKKAFSQDVHRVSFCANGT
Gg   KPYR------------------ILQGGTKKGYVFGDFNHT-GTLLASIGSSPDYMLTIWDWKQEKIVLRSKAFSQDVYRVTFSPDNE
Cc   EIVT------------------ILYEGTTRSYSHLAYSPD-GLLMVSQGGDPDYLITVWDWQKSKIMLRCKSSGQDVFNVMFSPTVE
Da   DIVT------------------ILQNGTKKNYSHLAYSPD-GKLLLSQGSSPDYSITVWNWIKSMPLLRCKSYEQEIYNAIFSPTVP
Tc   EINC------------------VLKGGSSRLYSHLDFSPD-GLLLVSQGGEPDYLITIWNWPKRRILLRTKSYVNDVHRVKFSPYCP
Cr   EVVK------------------VLRGGTERAYSALAFDGERGDTLASVGHFPDFLLTLWDWRQEAIVLRAKAFSQDVYGVAFSPYFE
Vc   KCIKCIFLVIAANRVYRPRASTLFGQCTVMAFTRMFILRLLGDTLASVGSFPDFLLTLWDWRSESIVLRAKAFSQDVYTVAFSPYFE
Tb   TEVG------------------EFVKGATKGFSACAFNKD-GSMMATVGMYPDFFLTVWDWESRGMVLRSKCHNTDVYTVLFSPFDS
Lm   APSK------------------TFAGGAEVGYSTLAFNVD-GSRLASVASAPDYTLAIWDWENTSLLLRSKCFNSDVYSVAFSRFDD
```

```
   349
Tt  NILATSGLGHIKFWKVAETFTGLKLKGEIAKFGQVELSDVIGYYIHQDGKVLSGTEYGTLLLWEGNLIKCVIYIDEETQT-------
Im  NILATSGLAHIKFWKVAETFTGLKLKGEIAKFGQVELSDVIGYYIHPDGKVLSGTEYGNLLLWEGNLIKCVIFIDEQN---------
Pt  NQLATSGMGHIKFWKVAETFTGLKLKGEIAKFGQVELSDVYAFYPFPDGKVLSGTEYGRLILWEGNVIKVVIGISEEE---------
Hs  EQLTTSGSGHIKFWEMAFTFTGLKLQGSLGRFGKTITTDIEGYMELPDGKVLSGSEWGNMLLWEGGLIKVELCRGTSK---------
Rn  EQLTTSGSGHIKFWEMAFTFTGLKLQGSLGRFGKTSTTDIEGYAELPDGKVLSGSEWGNLLLWEGSLIKVELCRTGMK---------
Dr  GQLTTCGSGHIRFWNLASTFTGLKLEGLLGSFGKTTVTDIEGYVELPDGKVVSGSAWGNLLLWDAGLVKVEICRRGGR---------
Gg  EQLTTAGVGHIRFWKMAHTFTGLKLQGALGRFGKTAVTDIIGYVELPDGKVISGAEWGNLLLWEGGLIKVELCRAGHK---------
Cc  GHLTSCGSGHIKFWKMAETFTGLKLKGELGRFGKTEISDISGVYPMPDEKVVSGCEWGNILLWDEGLIKLEVCKKNKK---------
Da  GHLVTSGSGHIKFWKISKTFTGLKLEGVLGKFGKTEISDIVGLYSMPDEKIISGCEWGNILVWEEGLIKLEVRRKNNR---------
Tc  GQLTSCGISHIKFWKMARTFTGLKLKGELGRFGKTEFSDILGVLPMPDEKVVSGCSWGNILVWDAGLIKLEVFRTLRR---------
Cr  GQLTTSGQGHIRFWRMASTFTGLKLQGAIGKFGNVELSDVAAFVELPDGKVLSSTETGELLLWDGGLIKVVLTRPGSR---------
Vc  GQLTTSGQGHIRFWRMASTFTGLKLQGALGKFGNVELSDVAGFVELPDGKVLSTTETGELLLWDGGLIKVVLLRPGGK---------
Tb  GLLVSGGAGHIKFWTMANTFTGLKLQGLLGKFGRLEISNVSGFVVLSDGKVISGSESGLIILWEGDLIRCCFAREVDREDDDGTAAT
Lm  ALLVTGGAGHIKFWMMADTFTGKKLQGSLGKFGRHEISDVAAFVILADGKVLSGSDCGDLLLWEGDLIVCTFARAFAVDEPGAKGLG

   436
Tt  ---------KCHNGCINVVFKH--GDDIVTAADDGYIRFWDYATIDNSESDDFGNFYMKPNSEILIKTDD-------------DRPA
Im  ---------KCHDGCINAIFKI--DNNIITAGEDGYIRFWDFQSIENSESDDFGNFYLKPTNEILIKTDE-------------NRNA
Pt  ---------PCHKGAIESIFLY--KDCIISGGKDGFLRSWKLTELDQAEGDDQLNYYTQPIKSLELVDDG--------------ENA
Hs  ---------SCHNGPINQIMLY--EGEVITVGSDGYVRIWDFETIDTADVIDETGLLEIEPINELQVDKN-----------VNLFSM
Rn  ---------SCHNGSINQIMLD--EGEVITAGSDGSVRIWDFETIDTADVIDDSGLLEIEPINELHVDKS-----------VNLFSM
Dr  ---------VCHSGAVQQISVE--EEELITVGSDGAVRSWSLESVDAADAADDSGVCEIEPMNELLVGRN-----------VSLVCV
Gg  ---------PCHNGPVSHIVLD--EGELVTVGKDGFIRVWNFEAIDTADSVDETGLLEMEPMNELRVGRN-----------VSLSFM
Cc  ---------PCHDKQITQFEYN--NGELTSVGMDGWIRIWFYETIDQADPPEGDQFLEIEPIYEFHITEEDEDDYDAEQSSMLMCIQ
Da  ---------PCHSKMITQFEYC--NGDLMSIGMDGWLRVWSYETIDQTDASQDKGFIEIEPINEFEINIEKNVFDTGSTSSMIMALE
Tc  ---------KCHDGPIVQISYN--EGELMTVGMDGHVKVWWYEKIDQADPPDDDRVIQIEPTYDFHTEGL-----------MLMCMR
Cr  ---------PCHDGPIEALLLDRPAGRVLSAGADGRVRMWDFGAVNDAEPREDSHSLELSPLDEVVVAEG--------------AAL
Vc  ---------ACHDGPVEVLLHDRASGVILSAGADGYVRVWDFNQVNDAEPREDGHSLELKPLDEVPVAPG--------------AHL
Tb  FMARSYDYTPCHEGAINVVELMEGGRVLMTAGDDGYFRFWRVSELEVAEGEGAPPLYVPECLGEILVHAG--------------AFI
Lm  QPPHDIQ--PCHDGAIHFVSLQ--GRYVVTGGDDGYIRYWDLHELEVAEGTGLPPYYAPNCLHEVCVSSA--------------FRV

   523
Tt  NITQILVQENFWIVIDANGRIQKIKISENKT---------DYEMETLLEMN-SGRLNDIIVSPIQNGAITVGDDGAVRLWDYVQRKE
Im  NISQILLTDNYWIVIDFNGKIIKLCLKDNYT---------SSEQTTILETN-SGKLNDIAVSPTQNASVTIGDDGVVRLWDFVNKRE
Pt  QIIQVVTDEHFWLIGDQGGRVWRVDWETGKK-------------EIVYRNN-SGAYLDLAPSPQLNAAITVGDDGAITLWDYVKSEQ
Hs  IKMNE-TGNNFWLAQDANGAIWKLDLSFSNI---------TQDPECLFSFH-SGAIEAVAVSPLTYLMATTALDCSVRIYDFASKTP
Rn  IKMNE-VGNNFWLAQDANGAIWKLDLSFSNI---------TQDPECLFSFH-SGAIAALDVSPLTYLMATTALDCSVRVYDFASKNL
Dr  VRSPE---SSIWFAQDAHGCIWKLDLSFSNM---------TQDPECVCCFH-AGPVQGMDVSKSSHLMATTALDCSVRVFDVLSKRE
Gg  SKIHD-CGQPFWYAQDANGRIWKLDLTFSNV---------THDPECLCTFH-SGRIEAMSVSPMTYLMATTALDRSVRIYDFISNSQ
Cc  KQNIEDAEENFWYAQDGNGGLWLIDLNTFET---------PERPRKIFTCH-AGPITDMDVATWGPFVATIGRDAHLHIYNYFDKTL
Da  KKYPEDSEDKKWYGQDGNGGLWLIDLSTFDEAVNSDDKKFPNATRQLLRCH-AGPVMDMTACTWNCLVATIGSDCQLHVYNYANRKL
Tc  KRYP-DPKDTSYFGQDGNGGLWIIDLNTEET---------PKESVQLYKCH-AGKVVAIATSPNGPYLASLGEDGRIYVYAYLEKRL
Cr  SALLADSGGRRWVVADKAGNVYTVALPPAGP-------VGKGAVVTRVASHPAGAVAGLQLSARTHTALVASADGCLRALDYVSGAV
Vc  VSLLWE--GRRWVAVDMAGRIYVVALPVSGA-------ISKGASILCAGRHPAGAVAGLVLSPRSHVCIIASRDGQMRVLDYRTGAI
Tb  RSVTYCKDVDEWVVLDSAGVLWRVPYVHPDDILNNAVTKPKEQAVPALEFN-GGSITSAALSPIDHTVVTGGEDGTIRLVDYVTPRE
Lm  RSVTLDEEHRQWVILDEFGTIATLPYPLRNG----KLDTTERVRQVRLRLN-GVPLTCAAVSPVEHVVLTGGMDGVVRCIDYVYCAE

   610
Tt  HYHRV-------FQGKATCAEWLPYTMKNK--SARIILAGFSNGIVRWLLLNQ-------------------NGFHLLRATKVHNES
Im  YYSRK-------FPSKGTCVEWLAHTRKN---GGRVVVVGYQNGIVRYLLLNQ-------------------ITFTLLKSLKVHNKA
Pt  TYKRK-------FAKKATCIQWMPFTMTN---KGRVVVVGYSNGIVRFLLLNQ-------------------DTFVLLNVQKVHPHP
Hs  LAQMK-------FKQGGTALVWVPRMVNF---TGAQIIVGFEDGVVRILELYD----PKGLTIFAGRKKILDADIQLKQVFKPHTAC
Rn  LVQMK-------FKQGGTTLIWAPLTVSI---SGSQIVVGFQDGVVRILELFD----PKGLTVFAGRKKLLDAELHLKYVFKPHTEV
Dr  LTMSR-------FRQGGTTLTWASDAV-----GGGLLAVGFEDGVVRLLELYN----PQGLHAVAGRVHSGDAELRLTHALKPHNAA
Gg  QSEIK-------FKQGGTALAWAPHVVNP---NGSVIAVGFEDGVVRIIEVYD----PKELPTFAGRANIRNAEINLKQAFKPHTTA
Cc  ILVHK-------FCDIGSKVVWFPCGVEA---TGCTLVCAFSSGIIRMITVSI--------LQANRTKKIKGDYVRLIQVVRPHKMP
Da  VGAHR-------FNDGGSAVIWLPCSLDK---TGSTIVCGFHTGVIRVVTVGF---------------NDNSGHVELIQVVKPHTDE
Tc  IFNYQ-------YPAKGTCMIWVPPKLAI---YGDELLMGFDDGTLRLSILTI---------------NEDKKEVRVIQTTKPHDKP
Cr  LAEAA-------TPQRITAFTPLPAASPACPGGAMTAATGYRDGVVRLHARCA-------------------EGLALVGVAKAHKGA
Vc  LAEAP-------GSQPITAVAAIPASTGV---AGMVTVAGYRDGVVRVYTRSGPLVDLDAPDGVDADTSPFPQNLTLAGVAKPHKGS
Tb  LYKMC-------LPQPNVVIGLRFFQKDP---EKKKFLACCKSGAVLLVKRGS-------------------TAFTLLGQWRPHNDG
Lm  RCRMEWPRHPRKDPVPVADMKVLVPRSTA---QSVLAVAGFGDGTVRLLEIGE-----------------GSGSIRMTGQWKAHADG

   697
Tt  IKFIKVSPDGSIIAIFSESGDIFLIEQ-----HPSDFQKIDPFCLFETKFKI----------NSVVWDRMGEK----LLLACDDGNI
Im  IKYIKASPDGSIVAVLSIDGNLFLLEN-----DPSDIKKLEPICLFDTKLQI----------NSMVWDRNGEK----LLFAAEDGKI
Pt  IQKIIVASNTNYVAVLSTKGEIFFLKY-----SDSNIQDIQAYCLWETKLSI----------NDFCFDKTGTK----VLMACKDGYL
Hs  VTALAYERDGEILATGSKDQTVFFFEV---------ERDYKPIGYINTPGPV----------CQLMWSPMSHPEST-LLIICENGYI
Rn  VTALAYERDGEVLATGSDDKTVFFFDV---------EKEYKPIGYFNTPGPI----------RQLMWSPHSHPEST-LLIICGNGYI
Dr  VHAVTFDSTGHTLATGSADGTVFFFAV---------GDSYRPLGFVCVPGPV----------RSLQWAPPTHEMST-LLVFCADGHV
Gg  VTALAYERNGDVLATGSKDKTVFFFAV---------EDEYKPIGFIHVPGPV----------QALQWSPPSHVASM-LLILCENGYV
Cc  ITSMSLNNTHSLLVTGSEDDTIFVFFV----NTTATYPILVPIGFVKVPSPV----------TCMTWKP-NHDMT--ILVGCLRGDC
Da  ITAIVANPPAKLLITGSKDSTIFLFSI-----EVHEFINLTPIGFVEIPSAV----------TRITWKP-KNSST--ILIGCAEGHF
Tc  ITQISINPSNSVLVSGGEDSTIFFFQI-----KFTIHVELVPIGYIRVPNPV----------TCLTWHC-VNPYV--ILVGCLRGEM
Cr  VAALAVSADGGRLVSAGEDGSVFFFDL-TAQPQPGTGVPGMPACGLLAPRAFIKLPSGSGTVTCGVWEA-AEGGG--VLLGTNRGTI
Vc  VLALAVSQDAARLASCGEDGTVFFFDLASLGSTAANGVPAAPPCGVLVPRAYVRLAQGTGTVACATWS--ADGVV--LLLGTSRGTI
Tb  LALFAVDAAEHRLCTIAH-GTVFFFTI------LDDFSSLEPIGFCKIPLPGA---------TCVAWDD-ASSC---CLIGFECGKL
Lm  LCALAVNEDASQVASISPSGHVFFFSV------ETAPCTLDPVGYCVGPLQNP---------TSAAWDTQAVGGHGGCLLGFEGGQL
```

```
784
Tt  HEIMVPKKD---QCDTSETYLKEFR-SRSYKIRMMESQKPKTEEMELEFLLRR----------------------------------
Im  YETQIPKKE---NCDTSETYLQNFY-AKSYTIKMMEFQKPKTEEMELEFLLRR----------------------------------
Pt  YEAIVPS-----DVDNSENYLQEFN-ANKYLVRMMESQKPKKEDLDLQFLMK-----------------------------------
Hs  LEAPLPTIKQEEDDHDVVSYEIKDMCIKCFHFSSVKSKILRLIEIEKRERQRELKEKIREERRNKLAAEM-----------------
Rn  LECLCPTIK-DVDDQNLVTFAIPDMLVRCFHFSSVKSKILRYMEVQRREKQKELREKEREERKRKLMEEK-----------------
Dr  VELQSPAAD---TQICGNSFQLTALPTMHFCFSSIKSRIKRDEAVALRQARTAERQKQQQERLRKLKEQN-----------------
Gg  LQVPAPLPE---KQDTASTYHIKNLPTEYFHFYSIKSRIKLEEEIALRERKKQEKEKAKLEWIKQLQEMG-----------------
Cc  VEVTLPDVP---QNYTKMSYELVRCELRIFKFQSVKSAIRRALIQAEIQKKKEEKLAKKKQALEQLRVDN-----------------
Da  GEIQVPEKF-PSNDEHRKTYRLDEYRAKFLKFRSVKSALRREMVRQENEKKVMEKREEMRKKLDEYRSLN-----------------
Tc  VQVEVPKEP----QPYTTVSYLLDLEPKTNKFKTYKAQIRRDLKLKEIEERKAEKVAKKRVEMEKLKAEN-----------------
Cr  LSVPLPPPD----LNTHHSYEWAAGTSAVSSYQLVVPKPKRPKKKKGKNDGEEGD--------KEGGEQDEGQGGEDKGGEQAD---
Vc  LQVSLPPKD----FDTHHSYEWTE--AVVHSYNFCVPKPRRPKKDKGGNSRKEGDDAGAGSGGQEGGDQDQAEGDAAKSSPQATVAH
Tb  LAIRAPTRD---MVDQSVSYEFTCNYALVGIRQRKKVEKKQANVSAGEREGFVEE--------------------------------
Lm  LSIRAPAPG---SVNHDEGYEFPCAYDLVAIRQRQ-LPPPKTEAEELEVDDTAAT--------------------------------

871
Tt  ------------------------------------------------------------NKNEDKKEEVEWDPESIMNVCYYDYSC
Im  ------------------------------------------------------------NKNEEKKEEVEWEPDSIMNACYYDYSC
Pt  -------------------------------------------------------------LKDDKMPDVEWDPASIMNVVYYGD--
Hs  -------------------------------------GEDGEKEFQEEEEEK---EEEEEEEEPLPEIFIPSTPSPILCGFYSEPG-
Rn  -------------------------------------EALGEQEIQEEEEEASEEEEEEEEEEPLPEIFTPPTPSPILCGFYSVPG-
Dr  ---------------------------------------------PDASEEELQEEEEEEEEEELPPLYTPSPPSPLLCGFYCAAG-
Gg  -------------------------------------------------KEVEEEEPEEEEEEPLPSIYIPEEPSPILCGFYSAPG-
Cc  ---------------------------------------------PGIDIDEEAFLMDSEEEPVLPEIYIPKIPNRVLMVNYTPSG-
Da  ---------------------------------------------PGIDVDEETFLEEVEEPLQLPELYVPREPNPVQNINYTNAG-
Tc  ---------------------------------------------PGLDIDEEVFLADSETEEELEPLYIPEIPNRVIWMQSTQDD-
Cr  ----------------------------------GEGGSKEGGEEGRAAEEEEEEEADDEADGGAGGPSSTTGELISLTLAPNEPG-
Vc  YYSPPRKCTRIRCQRIPSACSLKGWEPVPWRVWSAAQGDDEGAEQG-GGDEEEEGDVEEDEEGNPDDLAASSAELLTIALAPDDPA-
Tb  --------------------------------------------------------------EEEEEYLGPWPVRLICPMADG----
Lm  --------------------------------------------------------------ALEEEDVGPWPIAFVVQLPGE----

958
Tt  TKILATVQGKFLGHIYIIDWN--------------------KDRPIDSLPIAKIPTVNMNFVD----DDILLISFKDGSWQLRHKN-
Im  TKILATVSGKYMGWIYLIDWN--------------------KDRPIEAIPVPKIQSLCMNFVDF---QDILLIGFKDGSWQLRHKN-
Pt  -KVFCTAEGKYLGCCYIIDLN--------------------KDRPVEMIPISKMQTYHLDYKD-----DVLTIGYKNGQWDIRHKT-
Hs  -KFWVSLGGYDSGFLYHCEFPPCDES---------SDFKEQKDEPIDVRYLADTEDNPIQTITFNINKVMMFCGMKNGAIRVYVLNQ
Rn  -KFWVSLGNYDSGFLYHCQFPPHLVK---------DDFQKLENEPFDFRILENTDDNPIRTITFSSNYTMMFCGMKNGAIRTYFLSE
Dr  -EFWLSMGGFDSGFLYRCQFSEDQS----------LDPAERRDEPLSFIPVQDSELNPITSISFSSSGQLLVCGMQDGSIRVYPAQD
Gg  -KFWLSLGGYDSGFLYHCAFSSNEHQ---------EDPENRQDEPFEVIPIEGTDDNPIHQISFCNSRLLMFCGMQNGALRVYPLQD
Cc  -TIWLSMSGFDAGYIYEYPDPL--------------PGNYINRKPTKSTMVYAADDTEINSCLFYKNRKYLFLGMEHGEIRICRVNP
Da  -SVWLSVGGYDAGYIYEYPSITGDDD----------EESVREAEPLRATPLRDAEDLELKCSLSHK-DSLIFLGLEHGQLRICRVDP
Tc  -VLWLSMGGYDAGYIYEYK------------------IEQKSDVPYFFKMVDEADDTEISSFVYNYNRKYLIFAMENGEIRVNKIKD
Cr  -ALLVTAGG--VGHAARKAW--------------RVRMGEPLAAPLLEGFASAPVTCLAHAGPE---GRLALLGSGDGLVRLQALEE
Vc  -TLLVTAGG--SGHAARKVW--------------RVKLGEALATPLLEGFTSAPVTFFGYS-AD---GNTVLLGSGDGLVRVQPLDG
Tb  -DFAIGAGGVELLYKYGLHVRYEGQKELPPLPPTGIEPPDYVEEPLMNLCYRDYTPEASSMSYS---GRYLVVICEGSQMLLRQLDE
Lm  -GFAIGMGKEELAYVYQGSIRYANQLELPPLPPTGVEPPDYVEDPALNVCYRDCVPRRAFVSPN---G-ALVVCADRNRLLLREATS

1045
Tt  -------DLKINMLVRTHDRDTGAIKKVVMNHEKKCVMSVGNDGTLFIYKIDLPSIKKITRGEIIEDF-QFEEFVGG-LGEHSFAEQ
Im  -------DIKIGVHLRPHDIDYGAVKNVCLNFMKNCILSVGGDGTFSTYKVDMEAIKQICKGNMVENFIFADQFEGGSISENTFSEK
Pt  -------DFSKQINKQSHDMDYGRVRKVGLTFDRTGVLSVSDDGTFYVYKIDFQNFLMQLSGNEVADFQYPEFTMG--INSGTFAEE
Hs  NDP-SLTSLVDYWHFNMHDNNYGCIKSIANSFDDRFLVTAGADGNIFVFNIFSEFMLRKDMK------------------AKVPSPR
Rn  NDP-FLISLQDYWHFNVHDNNYGSVKSIFPSFDDQYLVTAGEDGNIFVFDIFSEFIIQKEAK------------------AHVPSPR
Dr  L---HQGSLQAFWALSVHDNSSGRVRHVHFSFDDTFVLSAGDDGNIFSFSCMTEQQIHTHTH------------------ATVPSPR
Gg  KDL-SVNTLKAYWSLSVHDNDYGQIRGICCSYDDRFLITCGGDGNIFTFNILSPEDVHEELK------------------AKIPPPR
Cc  EDH---TDFSDYWILSMHDNYNGYIPKILLSHDQKMLLTCGHDGNLFSFLIN---DDTPPLT-----------------FHIPRAKE
Da  EDY---TNFSDYLSYPAHDYYRGDMNKILLSQDEKYLFTCGCDGNIFSFRVSFGEDRSVGKI-----------------EFENNDRN
Tc  GDY---HDLSDYWSLAMHDNQNGFVPNMCFSYDEKFFFTCGHDGNVFSYTFQPDDDDYIPPE-------------------DYPIVE
Cr  PFGSAAPGALPLWEAPLHDMQSGRVSGLGLSHDGAYLVTAAADGALHLLALALPPELAPPPT----------------TQPGDEPLP
Vc  PYG---APMGPCWESPLHDMQTGRVTAVALSYDGAYLLSAGRDGSVHLLALQLFGAVEPQEP--------------------PAEDL
Tb  MG---RVRLEPILVASAHDRLDGPIAAACTSFDDKMLVSVGSDGLVVAQLLDGCIAPQPPSP----------------------VAQ
Lm  -------RQQVFLLGAAHDSTGGAVTGAFLSHNGSMVVSIGTDGLVVTQLRDGCATPRASPP---LIDAATVVEPALGMVSATAAAP

1132
Tt  IELSDN-EENEIKDDSVYSIQEAKLLAEEDFKRNEAQKKKDKVKEVIKDLVRQFEQLKQQNSTIDEVARLTDQEMTVDHEFIEMLNK
Im  IIEEENQEEEDILDDNVYSIQEAKLLAEEDYKKQEANKKKNKILEQVQNLVSLFEDQKRKNLEIDEIARLTNQELCVDPDYIEMLNQ
Pt  IAFEEV---QDILDDTIYSIQQAKLLAEEDNRRNEANKKKETVKSRILQLREQFSQVRQKNQTAEDAAKLTEDELCVDPEYKQMLLD
Hs  FGIETEPIPEDIEDPKAYSIENARRKREHDKLMKEVGEIKARKREQIKALRSEFCNLLEMNEKLPKHMQFKRTDFDVDSQIRAEMHR
Rn  YGIEAEVAPEDIDDPKAYSIENARRKREHDKLMKKVEELKAQKRQQIKILRNEFWKLLALNKELPKHMQFHRTEFNIDAKIHAEIHK
Dr  AGLELEKAVQDIEDPSAYSIETAKQKLELERVCAEAERRKQQRRARLEELQRSFQELLKENQRLPQHTRLHRSEFELDPLFLQQLEM
Gg  RGLEKEKAAEDIEDPNACNIEEVKQKREYERIMKEAEDKKSKKREELIALRQQFLFLLQKNQELPKHMRLQREQYEMDRRVFEELNM
Cc  PLLLPNVNVEDIEEPDYPSLEEVIVKAEYNRIMTVAKHKKDRTLDVLRELAEEFGRIMESNRYLVKSQQIPQEEFELDPRITEDLNE
Da  ARPARKKDVEDIRDVNYPSLEQVIITTESNRIMSLTKKNRDDVYYLLRNFTKDYQGIMERNRQLIKSQQIPPSDFKLDDRIVEDLNS
Tc  ERTSFPNSVPDEANYDKLSLEQASLKKEQDRIDRLAAKHREIYREKLRKLRERFFKLLKRNKKLLPSQMIPVEDLEVDKRVTDYLDY
Cr  GPAALPLRPPDVLAAAAYTLEEEKQQAERDQQVREAEEKKLSVRQRLGLIRAEFEALLAENEAAPEALRLPRADLEVDPGLRALMEA
Vc  PDARLLLALTDITSPTAYTLEEEKQQAERDQQVRMAEEKKLSVRERLTRIRAEFEALLAENDAQAEALRLSREELEVDPGLRDIMEA
Tb  LQPLRAEEIVEPQLAP-FSITEQKDLDDRRRAEDEKRRELNLFLDKLKDVHQKYARLLRENQSLALTHRLSKEEITIHPQIYRELQE
Lm  AVVACAEEAVGAVEAPALSVQEQKEADDAARASTEREAKLQRFLNRLEKVRDSYAELVHANEQLPSGQRLNKDEMQLDASLQDALDA
```

```
   1219
Tt RVEEDLEETKEELKWDAEYCKLKTKKLTNYVKNELIMDTFSVKAIKKSNVIVSTFKVKKMSEFMKQNLEEIFEVIEEEKRNQEEARQ
Im RVEQDLEETKLELKWDAEYYQLKTQKLFKYMKDELIVDTFTVKAIKNPNIQVSTFKVKKMSDFLRQNLQEIQEKIDQI-EEERRIQE
Pt RVAEDVEETRLELEWDRTLAKMKADKLKAYMIDELEIDKFMVKGFRN-QASVQTFKVKKLSNFLIEQLKETYRLIEEERKNQENQPQ
Hs KTAFKIQQVEKELAWEKEKHELGLMKLKNRFRDPLESDTIVVHAILSDHKISSYRLVQPSKYSKFKRASQSERKPSKLDRFEKEG--
Rn KTSIKIEQVEKELAWEKQKYELGLKKVQDRFREPLESDTVVVYAIQSDHQIASYRLVKPSKYSKLKRPSQTDRRPSKMERFEKEG--
Dr QTEQRVQEVRRELAWGEEEQRIGLSKLQNMFWESVVQDTLTVFACRSGHKISSYRLLALPFTHTHL-------SSQSSPEEAELRQE
Gg QTAQRIQLVEKELAWEHEKHLIGLQKLQNWFRDSLEFDTVIVHAIQSNHQISTYRLVAISEKYYQDK-----ERPSWKKTVLKRAWK
Cc QLKAEMNLVHKKLAFKVEKSKLGLKKLMDHFIEPITCLPFAVRRILKPDTMVNSLRERKLGDEFTATLDEVTRRIE---DREKAG-R
Da QLKCEMDLIDQRMAFKIERNQLILRKLMDHFVLPITCLPFAVSRISKPETMIHSVKEKKLSENFQAEYADVLQKLSRAKDSEK-S-T
Tc KLEEEQALVKRKLAFDVQKSELRMNKLRNYFIDNLCFIPLIVSGLR-SERLVRMLRQRKVSPLNDQMLQIVDEKILEEEVKGRPP-E
Cr EALRREEVARLELAWESERQRLGLAKLRRYFLDGLESERVVLHSLRGSSTVTTFRVAKLSDETRAELAAMRQAARAAAAASAAAGGE
Vc EALRREEVARLELAWESERQRLGLAKLRSYFLDGVETERVALYSLRNGSIVTTFRTAKLSEETRAELAALRTAAAATASAAAASGDG
Tb EMRQRVEESRKPTALELARENIRTRKMRNRFVDNLAHDRFLVRSFSKEFSVASFRTPYVDGSIKLFQQQIDELLGSERCSSLACDGD
Lm EKQRRVREAEKEYILPTAREDVKTTKLKNCFLDNLLYDRFELAAIEEGFSVASLRVFDPHESIAKLEAAAHQLAESDGESDVEKEED

   1306
Tt NAMSQNLNQTNFGGDQTISNKGMQETVKKDQTLQKQEEDKLKKTVMPNMQGQQQG--DKKGETTHLSASKQEKLENKLKEQKIRQEM
Im EIFQKSPQNNLNLNDQTIKNQTFKKEVQNLQNTGN----------------------EKKGEQTHFSLSKQEKLENKLKQQRIKKEL
Pt IENKTQEKIQVQSQVKITAVDMKEDQQKATIQQMDPDQK--------------------------VPQAKLERERLRQEEERIKKEQ
Hs -PGRKDSQRDAGGSVTIQEESIIEKGKKFRPKTLSEIIVENQ--------------------IEKTRKLILKAERAQLKIQQRKKEW
Rn -PGKRESQRDTGGSLSLQEEVVLEKGKKARPRTLSEIMVENQ--------------------IEKTRKLVQQAERAQLKILQRKREW
Dr HTHPHKHHSSTSLQDEGPQVSSAGTHTHT---------------------------------QKQSSRAAEKVERAREQIQTRRQQW
Gg KKEKRESIRDRKPRVVKREADKLKTKVRKPTTCFIERK------------------------REQIRRLVEKSDKEKAKIMKRKEEW
Cc MVEVKSLQEE----EEDEEKQKVQGIESFLKGLSPSTIQYRL--------------------GVKINHMLRKYRSRKARMEERYNEW
Da MADESDDQGEGAGKGDQEEKLKIDGVASFLQDQNFFSDEEKL--------------------TAEMKQMLRRYNDKKLQIERRSKEW
Tc RAPPAPKTIQ-------PKKVKEQGLEYFLVNLDPKILESQK--------------------HNKLTRLLNKYRDRKLKWERREEEW
Cr GGAGGRDTDARGKGSDTGGGPGGDAAARARLAEATAALEEG-----------------------TASGKLNKADLRRLARKRREAEW
Vc IGRDGMNSDAR-KGSDTGNGPAGDAAARARLAEATAALEEG-----------------------TASGKLNKADLRRLSRKRREAEW
Tb RGIVSVASGEASQSPRNLSLSTAAGVVRWLNSEERKRNEGEQQKMNAA-------HREAEILTTTMRQYLNKMDERREERHWRKKGY
Lm EGSDSEQDGAGADVAATEHTAAGDSKAAGIDAAATTAYERPDDLIGIA------------TIGASVKQQLNKMDTRRRERQERRDGY

   1393
Tt EELEKKKPTKESFEPDHLRDINHAMETLGDYKLKSSPTYNVPENQRMNES-KKKKHMFLLDEFIYNTKMKFNNEIVSLRERKTSLID
Im EELSKKKPTKESFDPDHQKDIENAKKTLGDYKLKSSPTYDVPENQRMNVS-KKKR-MFLREEFIYNMKIKMNNEIIALRERKKNILE
Pt ELLEKSKP-IELAKPENAPDIQEAIKQYGDYKLKSSPNYEVPDNQRMNVS-KKRKHMYMLEEFIYNTKLKFNQQLLNLKKRKVNLID
Hs EELYKSKPGDDYEDPKDLQAIKEAQVYMGDFNLKTAPDYKIP-EHMRINAAKKEEELGHLDSLVHGNKRHMNKCILSLRDLKVAVVE
Rn EELYKSKPDDDYEDPRDVQAIKEAQTFMGDFNLKTASDYKIP-EHMRINAAKKEEELGLLDTMAHGKKRYMNKCILSLRDLKLAVIE
Dr AELYASKPSEDYEDPQDVEGIRAARENLGDCKLKSAEDFTVP-EHLRMNVEKKRAQLLELERKIFEQKSEMNLRVLALRDQKVALLS
Gg EKLYKSKPSDDYEDPRDVQAIKEVQENMGCYKLKTATDYRVP-QEKYMNTEKKTMQLASLEVLIYKKKVNMNKQIMSLRDFKVSIIE
Cc KVMNASKPDPSVNHPDDVLAIEEAKETIGNYKLKISPDFNPP-KGKRENTLTKYKQFLNSRKRAHHMREDFNSKLREVRSKKVALFN
Da KIMHASKPDPNSDHPDDIRNVEIARKTIGDYKLKISSNYDMEGQRERETTLSKYKQLLDCRKKCHYLRESFNDRLRFMRAKKLSLKS
Tc EEFLARKPLPGVNHPDDDVALKDAKVTIGNYKLKSDITYKAS-KNQRETTVKKYKELLLSRVKQSNTRHNFNEKVWALREEKIAIRD
Cr AAFNGTRPDDTYDSPADLAAIEEARRTIGDFKLKSDPNYVVP-EEERLTPQRKRLAMLELEEALHDIAAAFNAKFFALRDVKRKVLA
Vc AAFNATRPDDTYDSPADLKAIELA---------------------------------------------------------------
Tb EMLLAHKPDPAVEEASLNEELRRETRRRGECILRTDPSY-----HSAPSAVIKLQQLIRLEEIIFNMRNNFSNELLKLRDEKERLCG
Lm GRLLDRKPNPAIDDAALKQQIAMDLKNRGDCTFRTDAAYRGE-HQYRPTAVAKLQRMIHLMEDIVRIKTAFNEDLLQLREEKRHTLA

   1480
Tt KISNYNKR-ITEINKQLGINETLFTLTLDKQLEAVD-QFDVTEEQIDQYEQQKNKETAQQSKKGGAQEKTQQQQQSSEKQDDKEKD-
Im KINDYNQK-IQEINKQLNINETLFQPKIDKQLEMPETFFQVTEEQIDAFIISKNKENAQNAKNKQTQENEQQNNNNNNNQQ------
Pt KVKKYNQQ-IKKINEQLGKQEQLFQPEIDKELEDPHSFMEISDEQINEFISKLQQQQQKKDEQKRIA--------------------
Hs EIQCLVQE-LKNIQSTLHISKHIPIPKIPQIHP-EEVPEKRFQYDEETLLNFKQQQMKSKDEKSPGV--------------------
Rn EIQCLVQE-LKTIQSSIPPAKHMTIPQVPQIYP-EEVPERRFQYDEETLLRFKRKQQKQRQDDKSSNSS------------------
Dr ELQAQVEQ-LQAVQQTLPLQKHRPLPALPVLLP-AEVPERRHRYTRATLERFNTLRLQEEQDILELLEA------------------
Gg EIRCLLQE-LKSVQAALDISERLPLPLIPQLHP-DEVPEKRFEYDRDILLKFKEEQAAKAKLQEQLEES------------------
Cc EVIELVMK-LKEIHSEIPEERTKPLPIIPVIDQNIEFPEKNLELEKYTSMAERVKEAKRKKKSFIAEIVVSP---------------
Da EIESSVEKKLNAIHAEIPKHYIREMPEMRAIDASLEFPENNLKLEKYISMGEKVKEARRQKQSIIDETMQQA---------------
Tc ELQVLNEK-LDEIHKEIPDDCRKEAPIVREPED-CEFPERHFEITVTMVMEEEEYDEGSRKEPVIVTESKDF---------------
Cr DVRVKLAALAELAAAAGAATGGADPDATAAAYLAPFSGLPSGLLPEEEPAEAREAVTDADLAAFAARKAEDE---------------
Vc ---VMANSAAGGGTGVAVAGGSVDTDATAVMYLAPFASIPAALLPDEQPAEARDAVTDDDLAAFAARKQEEE---------------
Tb TLNVSLQRIRAINEKL-KDKSFHADDVKLTPEEMPGKRFEISRDGLVAFMKQRQEEKLREQTAKKAQRGFGADLATGEPATNTDTSG
Lm RLNCMRQDYRKVLFQLGLCTSPEVPDLSLTVEEEPETLYTMDRDKLEAYNKQKTEERAKAE-AMKAHRGFGADLAS-----------

   1567
Tt ------------------------------------------------------GKKEKKVDITAGFKPRKGAKVQGSELEDELKSI
Im -------------------------------------------------------KKEKKPDPTLGYKQRKGVKVQNSEQEEELKTI
Pt ---------------------PAQISKPAEQ---------------------------------NQIKTRKGIRVQQSGLEEEMKTI
Hs ---------------------EQTGSGGPVGG---FLKLSSRKDGDLTTRDSISRSSKASTFSLDIPKCLEFEKAEPTDVELEIMKR
Rn ---------------------KQSGSGASTGGGLGFLKLSSGKEGDLTTRDSLSRSSKAFAF-LELPKATEFEKAEPGDVELEIMKR
Dr ------------------------------------------------QDTHTEEDTHTQSQDTHAVEDTHSQEEELTLLEEQMREM
Gg ---------------------PSSGAFR------YSLKSSDKETGSLQQTASSDGMKRSSSVVIEQQKIFVIEKAEPTEMELEILKR
Cc ---------------------NDEEYEVLLFDEKSIYIGDDDMQSTVASVVPETKYSVTNAISGDVLQTLNMSDHIETPCEREMKRA
Da ---------------------LDDEFKTILPDESLFEYYSDLLPTSLNIKEKKRIKQVDIAIPNHVQKMMQDYDRKITPWEREMKAV
Tc ---------------------DAIEKHVLPKN----------------TKKFDAKECDVALLDFESLCSFSKNEKKDTLWEHDIRTR
Cr RKAAAAAAGGLGGFAGAAAGPKKPAAGGAAPAGGALAGGAAGSGSVAHGAGGPSAGGQQGLTAAEEALAKMMAAVPQSELEKGLAAY
Vc RKAAAAAAGGLGGFA-TAGGVKRTAGGVAVPAQPHGGAGGGGGAATSGSIGGP------GLSASEEALAKMMAAVPQSELEKGLAAY
Tb ADTPATRRSEGEDSRKVTISANRDSFGTAAARTRSVRTGTLAASKGGRPFSGGGFAAGAARERMNHELRVKHENIKLTEMEEEELQI
Lm AQAPERSR----DSSTVTTGTVPGSVIMPQKRSK----GSHFPSYLGRGRSRQSAVGSLAMRERMDADLKGKLNNVTLTPEENEEREM
```

```
   1654
Tt NQMILNAEKKRMLDEINEEIEEFDKQVVNCQNEKKCIESDMTIAQMKFVTYYQELLILADMEEQDEKYIKDLLNHKREKADLEEQSS
Im TQMILNAEKKRMQDEINEETEQFDKDVLKWQNEKNIMESDITIAQMKLVTFFQELLILADMEEQDEKLINDLLNLQKDKSELEEQNA
Pt QDMVLNAEKQRMIDEMQEEIVQFDKDVVKCQNEKNVLESDMLIAQMKLVTYYQELIILEDMEDFDNKLIKEVLDFKKEKQNLEQQVE
Hs DEIKHVYMQQYLVNRIKELVVTFDAELRLLRHQKLKLDTQMKLSDLHHVTLFQEILLLKNFEKQENILQERVNSLDKEEQYMQWKIN
Rn DEVKHLYMQQFLCNRIKELTVTFDAELHLLRHQKLKLDTQMKLSDLHHVTLFQEMLLLKNFEKQENILQERVNSLDREEQDMQWKIN
Dr EEARGLNLQEQLLTQMQDSVCRFDAELCVLRHEKQQLDVSLKLADLRHVTLFEELLLLRDFQKREEKLTERLRTYRQEEDDIQGRMR
Gg EKIKNLYLQETLINKINELVTNFDAELRFLRHKKLQLDVQMKYADLNYITWFEELLILKNAEKNENLLQGHVNTLQSEQEAMQSKLN
Cc RVMRKIYEQDSILRHIHESYEILDNQLDELERERLEIVAESVYMDLYMLTLHQELIILKEFEAMENTLEEKVNEKLKERATAKQKMQ
Da RLSRKLYEQDCIIKDIESRYEDIDRELDELESMRLQVYADGVYSDLYKLTLHQELIVLRDSERTENSLREKLNTKLQDKAVALDKLQ
Tc RLQRKLFEQDVIIDEMNRKITDFDDKVEKLNTERFQAEVDAKIIDIFMVTVHQELLILKEFEALEYTLQSKVNQKMSEVLDMRDIID
Cr NRRRVEHMRSKLSEEITAMLDAFDDAHSALKAEKLGLEADVKAGQMRLLVGLQELQLLREFDKRESVLLAKRQAKLDDKQEIVDKIA
Vc NRRRVEHMRNKLTDEVNSMIEAFDDAHAALKAEKLGLEADVKAGEMRLLVMLQELQLLREFDKRESVLLQKRQAKLDDKQEIVDKIT
Tb ERNRLLAERQRLHTQVQAMMDEFDVRLWSMYEERSRVDANLCLAHTHSLLLFREYNILLVFRQKDFELQSSYDEARNSRDRCLREME
Lm QRAVLERDMRRLKAAMAAVRTEFNDKLGSLRLRRAALDVDLCLMTRCLTLFHREYQLLLIFRSQDKKLRAVLTAVKQERESVQRGIA

   1741
Tt KIVGELTALANKDSQNEKELTEQMKVFKELVYPEDDNKREKIHQYYLRKYKKNKAKQLK-LRNHEE-ENEDFDEDEMDSEDDDDDDF
Im KIVADLVTLANKDSQNEKELVEQQKAFKELIHPDDDVKREKIHQYYLRKYKKNKAKQQK-LRNAEE-ENEDFEEEDIESDEDDDDDF
Pt KILENLATLQKKEQQNDKDLIEQLKAWKELVHPDDEQKRNKIHDYYRKKFKREMLKRLKKQQEKEHSDSEENEDDEENEDDNDDDIV
Hs ETLKEMEEKKNEITKLQEQEKALYAGFQAAIGENNKFANFLMKVLKKKIKRVKKKEVEGDADEDE--ESEESSEEE-SSLESDEDES
Rn ETLKEMEEKKNEINKLQEQEKSLYSGFQSALGENNKFANFLMKVLKKKIKRAKKKEVE-EADEDE--ESEESSEEE-SSLESDEDES
Dr ECQRQMELRKRDVQRLQNREKSISTSFQTSLGENQRFEEFLTRVFKKKIRRTKKKETHTAQDGDQSEDEEDSDEDSDEDSDAEDEDD
Gg SYLARMEDKKSEIVKLQECEKALYASFQASLGENNEFAHFLTKVLKKKFNCVKKK-VERETDEKD--ESEEESDEE-SSSESDEEDS
Cc AMNIKIEQKIREIAKIQEKIKDVFSEYMSMINEN-RFYEFLRRIFKKKYKP---PKTDDESSASS-SSTTESSTDDDEAG--SIDSR
Da SMMRNIDKKNRDIKNIQNKMKEQTAYFASATQDN-KYAKFLTRIFKKKVKIGVAQRSESSSQMSS-DSSDETSTDQDDSDNSSVDIH
Tc EISSKIVEHNSDIEKAQEKIKSITVSFTNAVQDN-KFFDFLRKVFRKKYKPPKVHKDDGETESSSESSSSSTSSTESDDDGKSIDSR
Cr ECTDKLETKRLELEGLVARRAAVVAELDAVVPESDPFR--------EALVRVFHRRIKRSKKKAGGGGGEDDYDSEEDEEDEDMGDDE
Vc ECTDKLEQKRVELEGLIARRAAVIAELDAILPETDPYR--------EPLVRIFHRRIKRSKKKAGG--GDDDYDSDDDEDEDEMGDDE
Tb ELQRLVQDQTASIEKLQEANKVFRREVEIFISNSFPAEH-----VPYITKVFLRQIKRRKHHSDMSGNDDDITSDDDDDDD-MGEDE
Lm SQHTDLASSEERIRQLTGQLKSTNQAAEAYIDEHCPSDK-----LTYIKRVYYRQLKRRRRAEDGDDDDDDITSDDDDDEDDGIG--

   1828
Tt MDDDD---DEKPDITPVENDT-KIREII---DKICDLEDQQETNRKEKADLERQKNQISVKIQSTLKELQKAEEKLRAYQREKLQK-
Im LDDED---DEKPDISPVENDS-KIREII---DKICELEDQQENKRKEKSELDRQKVSILSKIANIGNNLRKAEEELKNYQRKNFKKL
Pt SDDDE---EEKPDISPVEQDP-KVRDVI---EKICGLEEQQDNFKQEKIQNEQQKSKLANKIAQNDIYLKRANDDLTQYQRKKLQR-
Hs ESEDE---VFDDSICPTNCDVALFELALHLREKRLDIEEALVEEKKIVDNLKKEYDTLSKKVKIVATNLNAAEEALEAYQREKQQR-
Rn GSEDE---VFDDSICPSNCDVSLFELALQLREKRLDIEEALVEEKKVVDNLKKEYDTISKKVKVVATNLNAAEEALEAYQREKQQR-
Dr YEESEAGAALDDTVCPPNCDAALFENTLRLRDLRVDVEEQLQDQMKTLDTLKKDYDTLSKKEKLVQGSRRSADSDLELLNREKQQR-
Gg GSEDE---IFDISVCPENCDKALFQNTIQLREKLLDIKDALIREKKVAAKLRKEYNVLFEKAKSIEASLNTAEKELDIFQWKKQQK-
Cc EIGPI---HVDENICPLGCDKELYAMAFSMRERRYEYELQVKEEQKSIELLRKEIENDTKKMKAIENNLKYNQDELEAFMREKQRK-
Da KIALT---TFDESVCPPDCSRELYDLAFSMRLKRHDNENEIREEQKNVESLLREVDHDSKKIKIIETAVNDCKQTLNNFMHEKQCR-
Tc DFGII---KQDLNVCPKGCDPSVYEYTINSRSERHQVELLIKEVLKMIEVLKKDLEIHNKKVKVIEKDLQTCQDELEKYQRQKQDK-
Cr VDDDD---DGGEEVCPPGCDQSVYERVCDLREKRLDEEDMIAEFTKTIEVLRKEKEALAKKQRLVEQGLAAVNADMAEFQKEKQGR-
Vc ADDDD---DGGEEVCPAGCDQSVYERVCDLREKRLDEEDMIAEFQKTIEVLRKEKEALAKKQRLVEQGLAAVNADMAEFQKEKQGR-
Tb AW---------EEICPPNCSEERWCEVIEKREVRLDYVDAITEERRQLEATEQRIEEHKALADKNNAAVSTCLKAIEDFQGEKRKQ-
Lm -----------EEARPPNCSVEAWEGMLRCRESRLDLTEASDAAKAEVANIRKQLEQSEQRLQELAEHVKQCRGEVQVLEEKKRKE-

   1915
Tt VNQLDVSFC-LQVDQIKNLIEMGNQQQ-HILPEDLK--KSILFTQNERNKLSLRIEQLRQEQKEIEQSKKTLEKRKRYLQIKINKKN
Im INQMSLIEQLNIFQSIYIFIKLQEKKESYKLPDDLS--RSILFTQDEFNKIAKRIKELKLEQERIKKSRANLEKKQKVLQVKLSKKN
Pt VNQLDVSFV-LKLSQVQNLNEQ------KQLPADLN--KSILFTEYDFFKLCKRVVELKHEQGKITKQKAQKDKTKKMQEKEIKLKD
Hs LNELLVVIP-LKLHQIEYVVFG-------EIPSDLS--GTLVFSNHALRRLQERIHELQEENSKQQKLNKEWRERRKQLIREKREMT
Rn LNELLVVIP-LKLHQIEYVVFG-------EVPEDLS--GTLIFSNHSLDRLQERIVQLQEENAKQQKLNKECRERRKLLIREKREMA
Dr LNEVDVVLP-LRLHQLECGSEG-------LQPADLQ--SALVLSAVELQRLQMRIRELQQEKSQQRLQYRHARQQHRQLTPQLTDME
Gg LNDLYVVVP-LKLHQVEYFFNG-------EIPRDFS--QALVFTNQSLEYLQKRIVDLHHEKIMQREIYKKAQKQYKQLVRDKKEME
Cc LNDIDITVI-LKFHQLQHFTGS-------STVAQIQ--DCTVFDKEKLSHLYARVEELQKETSDQKAKHKKNRMHLHRMNVDCKHME
Da LNDIDMTLT-LNFHQLEHFVDD-------DNLSKMF--NCVVFDTTRLSQLYRRVGELQQETCEQKMRHKKNCTHLHRMNIDCKYMN
Tc LNEVRTTVI-LRLDQMKHLAEE-------YKTSGIM--DTLIFSKERLSYLYKRVEELQIETEHQVSRHRLNVKHYTRMQTDCRYMA
Cr LNQVEVIVA-LRMHQIEYLLDG-------CLPDDLS--ACLVFSASQLRRLQARVDELEEEKAGLRAAHKELRRQHAALLRDKADKE
Vc LNQVEVVVS-LKMHQVEYLIDG-------CLPDDLS--AALVFSATQLRRLQRRVDELEDEKAALRSQHKDLKREQAGLLRDKAEKE
Tb LNMLETLVA-MRCGQVRCLDEEG------RCPDTFRRNDLVVVSDKVITGLHDRIRALAEEKHDRRGKLKSMVAEQQALQRERSEKQ
Lm LNMLYTAVP-LRLSQVRCLEENA------RLPSHLPEQPVVVISAEQMSALRQRIFDLADQKIQRRDEVARLTAELQPLKEARAASN

   2002
Tt ALIDELSKKYNEKHMLKFGDIIDLKILDALEPTKQ------------------VLEMREKFKEEEKTSILRVEKAKQMLQEKKNVLLQ
Im AEAEEQSEKCREKHFLKFGDVMDLSTIEQLEPTKQ------------------vLEMRERFKEEERQSIIKLERAKQQLQERKNYMLQ
Pt LKIEDLNKKYEEKHMLKFGDIIDLKILDALEPTKA------------------VLDMRAQFNQEEKEAQRKVSRAKEELSKVKQQLLE
Hs KTIHKMEETVRQLMISKFGRVVNLEALQTLSVNTT------------------LEELKIRKLRKELANAKEMKMWEEKIAQMRWELMM
Rn KTISKMDETVRELMISKFGRVIDLEALQTLSVNTT------------------LEELKIKKLRKELSNAKEIRMWEEKIAQVRWDLMM
Dr TRIRESEVRCEQLMLLKFGKLVELEVLQTLAGNRK------------------VEEMRQEVRERDARYTQELKHWQAEVVKAKACVTA
Gg ITIQRLEEKCSQLMMLKFGRVVDFEAVQARSVNIR------------------LEELELQIMEKEYEHSQEIKECAKRILDLEQKLMM
Cc LEIKKLKDNIKEEMMKKFGQEISLNALYEAVLRRM------------------VYDIKVNINGMISSFEKEINSVKEAYAERVNILEN
Da SQIKRLKSCVEEEMLKKFGCPISLASLYEAVVRRL------------------IYSMKANMTDNLK-YERQFKCLKERYDENILILEN
Tc TRIKYLQEVITDKMKMKFGKVIDINEIEVAMLKRTFGRDDLHDLEEVLLKKIVHDLRLSMMDIKGLYIDQLHFWKEKISQTQKELTY
Cr ARVAELEARAHDVQMLKFGQVIDLELLDRVSSSRG------------------TEELREDLKKQELAYARELAEWDAKINARMDELVV
Vc KKVAELEARAHDVQMLKFGQVIDLELLDKVSSSRG------------------TEDLKEELRKQELQYARELAEWDSKIAARMDELVV
Tb ALHTQWEEKIYEAMLLKFGQIVNLEVLESSCGSRE------------------VEQLKERLRLEELSWEKELRKRDKKIAVLREKLHE
Lm RVFEEWQEKVNEVMLLRFGQYVDLEMLESCGSSWA------------------IETKKEELLHLELKWARSVKKVENQITELRTRLQD
```

```
 2089
Tt  AKKENTSIITSITKLGQDLMKLSKKLDSTNKQLFKEDNEDKKNKQDLEKERQNYKDLVRFLSKEIEDLKTEIGLFKRKGGHIYTMVT
Im  AKKENTAIITSITKLGEHLMKLSKKLDLTGKQLFQEDNEEKKNKQDLEQERQQYKDLVRFLAKEIEDLKTEIGLFKRKGGHIYTMVT
Pt  VKRENTKIFTNITKLGKQQMDLSKKLTSGNKQLFKG--DNKEKRSDMESDRQSLEDLVKFLGKEIEDLKNEIGLYKKKGGHIYTSIT
Hs  KTKEHTRKLYQMNDLCIEKKKLDSRLNTLQNQQGNAFQG--PREADVV-AREEVTELIQLQAERISALKEEIALLRRKGSLILPPIQ
Rn  KTKEHTKKLHQMNDLCLEKKRLDSRLNTLQNQQGNAFQG--PRKADIV-AKQKVTELVQTQSEKISALKEEIALLRKKGGLILPPIT
Dr  VMKLNTERLQKINRLMTQKKTLDEELDCRQRRTVCQYAG---GLVDEQ-ELQRLQQLISGQSQDIAALNVEIQALSRKDGHILPPLE
Gg  LTKENTRKARQLNQFCLEKQQLETKLDSLKDDWGVEFQG--PRTADMK-EKARMESQLKRLAHDEAILREEINFLSRKDGHLIEQLS
Cc  LIQDNTEKLNLLTVLEEEKSKLRKILKHVPMTEEEIELS---KLTYQN-DISKLKSILKSQMHQKELFRNEIRNLSLKSRPL-PPIC
Da  LVRDNTEKLSVLTVLTEEQMRFHKIIKKWPETSKNIFQS---ELEWRK-DLAHLEGIIVNQKEQKEFFRSDVRNLKLKIRSL-PPVT
Tc  VLRHNTSQQELLTYLVNEKVELAQSLMSQEKKKLHIASVGDITRRYSE-EIEKLQAIISEQNQQIQELKDEIKLLRTKGMVL-KPKE
Cr  LTRENTACLNAVSELTAAQRRLESGLTATRKGLFADPVQQR--RAEVE-ERDALVALVNAQAAELDRLKGQLLALRRKDTSMYA---
Vc  LTRENTACLNAVSQLTAAQRKLETGLTATRKGLFSDPVQQR--KAEVE-ERDALVQLVNAQAQEIDRLKTQLLALRRKDTSMYA---
Tb  SLEYNTSLLQTIGDQESDRQSVERSLAQSTQKVVSKMYDSI-NVATEE-DRSNLRLLIAAQQEEIDALRTEVALLRTKGGHVYAAAM
Lm  KVFENTSLLQNLGDLETERQRVDSVLSQATSKTVQQYCGTT--VASKQ-ERAVLRELIAAQQEEIDALTAEILMLKRKGGHVYSQAM

 2176
Tt  SNKKNFN--------------------------------------------------------------------------------
Im  SNKKNFN--------------------------------------------------------------------------------
Pt  QTNQNFVQR------------------------------------------------------------------------------
Hs  SPREKEIQPADL---------------------------------------------------------------------------
Rn  PMQENEMRHMDA---------------------------------------------------------------------------
Dr  PVLRPAHTHTASGTGAHSKLTLSGRAVK-----------------------------------------------------------
Gg  LLTRNNQFLQPSSPQDLEAPDQIFPVLKP----------------------------------------------------------
Cc  SKKKSQKIRTEPHEDEQILAVIDIKTGDGYTIDKTQVKIKVDDVESNDYIEEEVELSDEEKEAEDDTKEREITDFSSSENTAMMIHR
Da  SRRKYLPSIKKICSKSDDEQGASVRDETWQEKKTAISEIFKDKIHDSALRIVRNLLSKIIQQYVEREEAKSIVDQFFNEISKKHSNI
Tc  VKKEEPEEEPVAWEHIKDWGEEIEEEEEESLRKPFSTEIVIPTTLQEKSQEVATELIQEMLEALDVKLTKRSNENFVKEILGSVMRG
Cr  ---------------------------------------------------------------------------------------
Vc  ---------------------------------------------------------------------------------------
Tb  AAGR-----------------------------------------------------------------------------------
Lm  M--------------------------------------------------------------------------------------

 2263
Tt  ---------------------------------------------------------------------------------------
Im  ---------------------------------------------------------------------------------------
Pt  ---------------------------------------------------------------------------------------
Hs  ---------------------------------------------------------------------------------------
Rn  ---------------------------------------------------------------------------------------
Dr  ---------------------------------------------------------------------------------------
Gg  ---------------------------------------------------------------------------------------
Cc  LLSRIIEMSLGDEQAEESTQEILNEVISNLPINGNHDDLEAGIERSVENIVAMLPQGNPERMEAMRQAVRESLSDIIIRLDSESKQI
Da  STDFIDSFMEICEKIEKRIEEISTKAGISGKIRDIILEYANEIISQLEIESRGIGVGSVEISWNVKPKLDEFLESMEIKKDITTEVY
Tc  LSMTELVEELVRNLPIEPDDVQRGIIETTAEQLYVVQEPDAEAEDKFFSCREILEDIVDEILLVKGEPHSVMARLITKLVEQLPIDF
Cr  ---------------------------------------------------------------------------------------
Vc  ---------------------------------------------------------------------------------------
Tb  ---------------------------------------------------------------------------------------
Lm  ---------------------------------------------------------------------------------------

 2350
Tt  ---------------------------------------------------------------------------------------
Im  ---------------------------------------------------------------------------------------
Pt  ---------------------------------------------------------------------------------------
Hs  ---------------------------------------------------------------------------------------
Rn  ---------------------------------------------------------------------------------------
Dr  ---------------------------------------------------------------------------------------
Gg  ---------------------------------------------------------------------------------------
Cc  KSDDTDPDAKVKSKINELLENVGIETENMQIQNELVQLIKNGQGVDAVMEWLSDKLPVNMNEESKKTAQEYAMVTLAEITEQIIMSE
Da  IMKFLKMEMGLELATDYVLTKLSTKPDSETAIAFREHASRTLREIQKHIDGESETETTAKSSNSGL---------------------
Tc  LKEQSSLEYIVKRMVTSELETKLNRSDVMSLLRSKEAREILDEIIQTVFDSGLDLLYLIKDE-------------------------
Cr  ---------------------------------------------------------------------------------------
Vc  ---------------------------------------------------------------------------------------
Tb  ---------------------------------------------------------------------------------------
Lm  ---------------------------------------------------------------------------------------

 2437
Tt  -----------------------------
Im  -----------------------------
Pt  -----------------------------
Hs  -----------------------------
Rn  -----------------------------
Dr  -----------------------------
Gg  -----------------------------
Cc  EPVSVQAEEVEENIVSTENQSVKDDMSVD
Da  -----------------------------
Tc  -----------------------------
Cr  -----------------------------
Vc  -----------------------------
Tb  -----------------------------
Lm  -----------------------------
```
