# Supplementary material for: Ciliary proteins Fap43 and Fap44 interact with each other and are essential for proper cilia and flagella beating
Source: Cell Mol Life Sci. 2018 Apr 23;75(24):4479–93. doi: 10.1007/s00018-018-2819-7 (PMC6208767; doi:10.1007/s00018-018-2819-7)
Supplement: Supplementary file 8 — Fig. S8: Multiple alignment of Fap57p homolog sequences: Chlamydomonas eustigma (Che, GAX79654.1), Danio rerio (Dr, XP_697139.4), Drosophila melanogaster (Dm, AAN71097.1), Homo sapiens (Hs, XP_005270577.1), Tetrahymena thermophila (TtFAP57A TTHERM_00105300, TtFAP57B TTHERM_00929540, TtFAP57C TTHERM_00052490, TtFAP57D TTHERM_000681920), Trypanosoma cruzi (Tc, EKG07864.1), Xenopus tropicalis (Xt, XP_002931652.2). (PDF 62 kb) [file 18_2018_2819_MOESM8_ESM.pdf]

FAP57

```
            1
TtFAP57C    ---------MIDKNKDITGRLKLDLLHGFGSLSSKGYRNCISILDQENFRKVVFPLGQYLAVKQIDFQDMNFIKLDEKTES
Hs          ---------------MSAVVAQTLHVFGLRSHVANNIFYFDE-------QIIIFPSGNHCVKYNVDQKWQKFIPGSEKSQG
Xt          ---------------MSVIVAQSHYIFGLRSPVTNNIFYFDE-------QTIIFPSGNNCVKYNVDQKWQKFIPGSEKSQG
Dr          ----------------MATVAAPSHVFGLRAGVKNNLLYLDE-------QTIIFPCGNNCVRYNIDQKFQRFIPGTERSQG
Che         ---------------MSMAAIAPRLVFGFRGDVGDNVAYSDD-------GSVVYPAGHNVVLYSADTKTQRIIPGTLESEG
Tc          ----------MPSPPAPPRMIDRRHVFGVCEKVKDGFQYLDD-------TNVVWVSGKSLVILDTQLGTQQLISCTPTCQS
TtFAP57D    -------------MAGLAQHIQLKYIFGMNNEIRNSLQFHDE-------DRIIYPAGYNVVLHDLNDKSQHYYQGTNEYRG
TtFAP57B    ---------MQLPSIGNTQHIQLKFAFGINTNIKNNLYFLDE-------EKIIYPAGYNIVQYNIQDKTQVYYQGLTDYRG
TtFAP57A    MAQQIVQSLQDEGTQSLKLDFSMKYVLGYRSDFKNNIFQYTM----NDQNRIIYPAGNTIVISNNE-NKQQFINCIPGTKG
Dm          MDEANKEDVKRASLSNSTVTIRPRLIYGLRSDVIGNIHFNLT-------KEVIYPVEGVLAFHDYVQNKQRFLRLPEDTR-

           82
TtFAP57C    IVSMAICTKKMVAAVCEKQYQQDASEQQQDQYHYQSKYSSQPR------------------PFLAIYSLSDKKNQMVENRS
Hs          MLALSISPNRRYLAISETVQEKPAITIYELSSI------------------------------------------PCRKRK
Xt          MQALAISPNRRYLAMSEKGLEKATVTIYDLASV------------------------------------------PFKKRK
Dr          MHALAISTNRRYLAVSECR-EKATITVFDLEQE------------------------------------------QNRRRK
Che         ITAICVSSNKKLLAVAERAEKAMITVYDLQTL---------------------------------------------KRRK
Tc          VTALAISYNHRLLAVAESS-KFPCIVIYVCDPS---------------------------------------GNPKLRRKK
TtFAP57D    ISCLALSPLKRYLALGVKGQRDPAI--------------------------------------------IIFDTFTQKRRK
TtFAP57B    ISCIELSPLRRWLAVGLKGAKFDNEKFDNPSIAIYD-------------------------------------TITQRKKH
TtFAP57A    ITCMTLSPSKRLLAWSEDCDSGIIVVFDLIKLDKLEKAKNQNYQEPSDKDKLDKQAEKDRRDRFEKEKREKIDKLQKEVKR
Dm          PEVISISSHRKLLAVVERHSKNGRYIS-------------------------------------------IYELATLKKRR

          163
TtFAP57C    FIYDKDTQMSSFVALSFSS--------DGRYLACLTG--------------------------------HPEYKLIFLDLN
Hs          VLNNFDFQVQKFISMAFSP--------DSKYLLAQTS--------------------------------PPESNLVYWLWE
Xt          VLSAPDLTAHEFVSIAFSP--------DSKYLVAQSG--------------------------------FPEWQIIFWMWE
Dr          VLTGGEMVVDEFVCMAFSP--------DSKYLIGQAG--------------------------------GPDWTLFLWMWE
Che         VLIAADAGSKEYVSMCFSS--------DGKMLLAQGG--------------------------------GPEYNLVLWVWE
Tc          VLQVPDLGSTEYISLGFSL--------DGRHLISLGG--------------------------------YPEWNLVYWNVE
TtFAP57D    NLGLNLGNLDRYNIKQWVSIAFPSQNQETKYIFSLSG-------------------------------AGGDVVLCWWWHE
TtFAP57B    ILNFKEVKIKYWVSLAFPSSQQ-----EAKHLVSLSGNCLSERHKDKESKDRDSKEKQSSAQQGSDKIEGEIIACYWNLSG
TtFAP57A    IITTLDCKSRHYVALDFNKI-------NEKRIVALSC--------------------------------APDWQLIYFQLD
Dm          HLELPSNHRNAEIQQMIFTN-------DSRSVALITR--------------------------------PPEETLIMLVLD

          244
TtFAP57C    SNKREIEIASAFLEERLRDVPQKENELKINKISVSPKDSHQVALLGPEIFMIVRI-QESSFVPITQEIKRMPKKKDN----
Hs          KQKVMAIVR-----------IDTQ-NNPVYQVSFSPQDNTQVCVTGNGMFKLLRF-AEGTLKQTS--FQRGEP--------
Xt          KQKVMATVK-----------PDSH-SNPIYQVSFSPQDNTQICTTGHGVFKLFRY-LEGNMKQTN--FQKVEP--------
Dr          KKKVMATVN------------ISN-NGPINQVSFNPQDNSKICVSGKGVFKIFHY-AGETLKQTN--AFKTDS--------
Che         KSKVAAVAK-----------TTNQQGSPIYTCAFSPGDGTMVSIIGQGIFKMFKL-VDGSLKLLNIALGKRDP--------
Tc          RAKIMATCAVLDENE-----LGSRDRRLLNQCSICPNDFSLVCVSGHGIVRFLLL-RDNQLRRASG-GLREPV--------
TtFAP57D    KGKCLGS-------------IDLGAQNDIYEISFNHSDANSICVTGNNFFGIYAK-IEGTYVREDNQQKQKPSVQNGENSS
TtFAP57B    SGKILAWV------------QVSLANIDAFEISINKNDTSFFSCIGRKVFKCFKK-IDSVEEGDRQADDKQKGEKQKGEKY
TtFAP57A    KQKTVIINQVP---------LKVGDNMRYTHCFFHPKEEDFIVAIGTGAIKPYKLGADGQFKQKDPPFVKKESKDQAHS--
Dm          KTSTVIEGRA----------TIPGSHGGAECIAGNPNDCNFMAVGGERTLLLMSK-SERGFSISNNLKVKYRVTS------

          325
TtFAP57C    -------------------------------------------CIYTDAVWFDN------DKIALSTSYGEIFIIQDSEVK
Hs          -------------------------------------------QNYLAHTWVAD------DKIVVGTDTGKLFLFESGDQR
Xt          -------------------------------------------QNFLCHTWLSE------DRVICGTDNGKLSLWESGDLR
Dr          -------------------------------------------HNFLCHTWMTT------EQVLAGTETGQLMMFESGRLR
Che         -------------------------------------------ASVVSQYWVPSDPSENKERLLLGTIDGEIIMMEGTDAR
Tc          -------------------------------------------TNYMAHIWIPSE-----NRLILSTENGDLILMENNEYK
TtFAP57D    AAQLPQPTLNIQVSAGNNNQNPQPEIKLENGNLISNLPADIVEKNFVAHLWMQCE-----SYLVVFVESGEIILCDSRGNF
TtFAP57B    KATLKNHCTQLNGAPQELS------------------------QDYTCHTWLTCD-----NNLIVCTANGEIIVCNENAEF
TtFAP57A    -------------------------------------------PNYLSYCILQDG------YMVIGTDMGEILLFQPSCEF
Dm          -------------------------------------------MAFLSLDLLMIG---TSDDQLILVENGEQKLAQKASDA

          406
TtFAP57C    QHIQRVLEDRDER----------------------------------------IPIDCITPFSKGLIVGCEAGNFAVWMKA
Hs          WETSIMVKEPTNGSKSLDVIQESESLIEFPPVSSPLPSYEQMVAASSHSQMSMPQVFAIAAYSKGFACSAGPGRVLLFEKM
Xt          WEINIGSKSGQTDHEKHSAYSQELQVTSAEHAAPAP-----------------VRINAILAYSKGFLCSAGPGKVCMFEKV
Dr          WEINVTSVQKTESESKQQYSDEPFSAL--------------------------PQVTAIVSYSNGFACSMGQGTVCLFEKT
Che         SVFNTDNG---------------------------------------------LAAASIVCYSKGFVVGQDNGVVTIFEK-
Tc          YPLPMSPSDG-------------------------------------------VSINTLIAYSKGFICGGDLGLIYIFERT
TtFAP57D    LQIMEQSPRYFIVSRSGM-----------------------------------IKIVAAVAYSKGFYVACSESIVLQYHYT
TtFAP57B    IEVLKQSPVFINKQS--------------------------------------WRIECINTYSKGFIVGAKDSSIYVYRAT
TtFAP57A    KTILASSPKNEQ-----------------------------------------FAIQCIQPYSKGFLVGGKECTILFYEKD
Dm          DTVDLMIDQEVFDRDKELQEQRFLPTQAVSLPD--------------------RRVLCMTAFPKGFAYIIFNRAFVFERVS
```

```
          487
TtFAP57C  EDPDLPYSGAQGKEKNQLIDQLHYLK-EWS--DKKASGVSGMDISSN-EELLVIAFKNNQIATLDLTKVIQQVPQNIKALK
Hs        EEKDF------YRESREIRIPVDPQS--NDPSQSDKQDVLCLCFSPS-EETLVASTSKNQLYSITMSLTEISKG-------
Xt        EDKDF------YKRSRDIRIPQDEYS--TDPTQSEQQEIVCMCLSPS-EEMVLISTNKGQLYSIALSSAEISKG-------
Dr        EKADL------YKKTRVIKIPPKRFS--NDLSLTEKQKISTLCISPS-EETLVASTDLGQLYSIHLSIAELQKG-------
Che       DEKEF------YRKARSFTIENN------------PSRVRYLAVSPN-EEHLICSLENNQMYSLLLSNSEIMKA-------
Tc        DNKEM------YRKVRTFKFNNDAENMGPPSELIPVILSLALSPPPA-EEYVSFMTSTKQLYSINLPNADFFKS-------
TtFAP57D  QDNDK----NPFTCVNQFKLKQ--------FVELKEIEIKSICMNKA-EDKLLIGLDNNQIYEIKIKPYHPETV-------
TtFAP57B  NDDKN-----RYEEVNRVLFKQYS--------DLKDLYVTGLSITPHSEDKIACSLSNNQIYSIKLKRDSIYSS-------
TtFAP57A  VDLKN-----PYKLCSKKIQFRDMK-----------AMITSLLLTPN-EEKLIVGVDSGQLLQVPFTSDSMQLN-------
Dm        KFKFE-------RKTILTVP--------TNLYAEHMYQILNLAIDHK-QETVIVTTSHCQIYVGILIVPETLKT-------

          568
TtFAP57C  RDHNQRDRRDVQFDYLYKGFHNGPISSIDVCIQRPLLVTCCSQDNSIIIWNYASYKCELARKFPVSLNNDEINRQVLLSVA
Hs        --------EPAHFEYLMYPLHSAPITGLATCIRKPLIATCS-LDRSIRLWNYETNTLELFKEYQEE----------AYSIS
Xt        --------EDAYFEYLTESWHSSSITGLSICVRKPLIATCS-LDRSVRIWNFENNTLELYKEYQEE----------AYSVS
Dr        --------ECAYFEYLSHSFHNNIITGLSTCIRKPLIATSS-LDRSVRIWNFETNVLELYKEFQEE----------AYSVA
Che       --------DEMNFDVLGTNNHQGPITGMDICIRKPLIATVS-TDKSVRLWNYVDRTCELTKFFADE----------IYSVA
Tc        --------DERVFEPIGQPFHSGAVIAVDVSVQRPHAVTAG-RDRCIFLWNLITGVVEFRKRFSSD----------IHSIA
TtFAP57D  ---NNDSSEDREVTLINHLNHTGPINSMDICKRKPIIATCS-TDKTIKIWDYEKKQIKISWAFNEE----------AFCLS
TtFAP57B  -------DSDEQIEQVSLAFHSGPINGMDICIRKPLIATCG-KDKTIKVWNYEEKTLELSWLFNEE----------AYCIA
TtFAP57A  -------EENSKCEPLFMPFHSDKITGLDVCIRKSLVATCS-VDKTVRIWNYSDNQLENSKEFEEE----------AYAVA
Dm        --------KQLKFEPLGVLIHTGEIIAMSVCAWKPIIMTAS-RDQTIRIWNYETALVELVRKFQVD----------VNIVE

          649
TtFAP57C  FHPTGYYLAAGFFDKLRMYHVLND------KLRTYREIS-VKNCTIIRFSNGGQYIAAGSPISKFNKGANMGGAGTSTNSQ
Hs        LHPSGHFIVVGFADKLRLMNLLID------DIRSFKEYS-VRGCGECSFSNGGHLFAAVNG--------------------
Xt        LHPSGLYVLVGFSDKLRFMNLLID------DIRSVKEFT-VRGCKECAFSHGGHLFAAVNG--------------------
Dr        LHPTGLYILVGFSDKLRLMTLLMD------DIKIFKEFT-IRNCRECTFSHGGHLFAAVNG--------------------
Che       IHPNGLQILVGFADKLRLMTVLME------ELKSIKELG-IKGCRECCFSTGGEYFAAVNG--------------------
Tc        MHPSGLHLLVGLTDGLRMMNLFHN------DVREFKNIG-IRSCMECRFSTGGQFFAAAHA--------------------
TtFAP57D  LHPQGFCVAVGFLDKLRLMNLCIHNSQNTTKNKAYKEISPFKGCKEIKFSNGGQYFAAVNSTSSN----------------
TtFAP57B  LHPSGLHMIVCLNDKLRWMNICLHQSSNSNKSKHYKEITQFKQCKEVRFSNGGHYFAAIDGTQSS----------------
TtFAP57A  FHPSGFHIIVAFTEKIRLMNIFEN------DLISFKELS-VKNCREIQFSNGGHFFAITNV--------------------
Dm        LSLTGQMAAIGFSDQLRITQIFMD------DLNIVKTYN-FPHCNAVRYSNFGHMMAAAYD--------------------

          730
TtFAP57C  FQNSNALQTGKKSVNYVINIYNAYTLENIT--CLKGHTGAVTDLIWTK-GDKKLYSCGEDGQIYVYTTDSWE---KKDIKL
Hs        ---------------NVIHVYTTTSLENIS--SLKGHTGKIRSIVWNA-DDSKLISGGTDGAVYEWNLSTGK---RETECV
Xt        ---------------NVIHIYCTATFENIT--NLKGHNGKVRSVAWSS-DDSKLVSCGLDGAVYEWNILLGK---RESECV
Dr        ---------------NVINIYSTITFEEIL--NLKGHNEKVQAVAFSL-NDSCLVSCGMDGAVYEWNTLSGT---RESESI
Che       ---------------TTISIYNTYTCENVG--NLRGHNGKVRSLFWSP-DDSRIISAGMDGAVYEWRLKDFK---REKENV
Tc        ---------------TTIHVYYTYTCELLG--HLRGHSGKVKTIFFVPPDDTRLVSVGLDGAVHEFNLCDFH---KVNDNV
TtFAP57D  ---------------HVIQVFKFFTGENPSQLVFKGHTGRVKCIAWSS-DDSFLLSCGLDGMILAWKLDQDFQ--HQQTIV
TtFAP57B  ---------------QLIKVFRFYTGEQPPSLVFKGHTGRVKSLAWSK-DDSLLASCGVDGMVYIWRIDNDSGDLRLYENS
TtFAP57A  ---------------SMVQVFQFYTGENPSNLVFRG-SGKVRTIFWEE-DDQGFYTGSTDGLVIYWRVDDNGPQ-KTQIAQ
Dm        ---------------NNIAITSVYKLDVLI--NLKGHNGIVLSVAWSR-TDKFLISGGAEGAIYLWDIETGA---RLQEIV

          811
TtFAP57C  KTPNVKLQSML-YNEPQGILMVTGPDDVNKGGYIQTVRFRDDR---------------------------------DEICD
Hs        LKSCSYNCVTV-------SPDAKIIFAVGSDHTLKEIADSL-----------------------------------ILREI
Xt        LKSCSYSSVAM-------SSDSKTIFAVGSDQTLKEISDSQ-----------------------------------IMREI
Dr        LKTCSYTGVAI-------SPDAKTFFAVGTDCSLKEIHNSQ-----------------------------------ILKEM
Che       LKGCNYSSVLA-------TPDCKTLYAAGSDKKIKEFEEAPGAG------------------------------TQITKEI
Tc        LKAMTYNCGVA-------DVG--TVWTAGNDQKLRQFDRTK---------------------------------LQPVAEY
TtFAP57D  PRIVDIHNKGVNFSGLTLTVDNKTIVAVGNDRHIHQAVINEQQ-------------------------------PVDKTDK
TtFAP57B  HKSIQLSCVALN-------MDTQMIYSCGSDRFLHQGFIFD---------------------------------SSQQARK
TtFAP57A  FNNLIITSITGL-----YNPDTTTQGLERILFVSGVSSSQENEGEKCVYKLVISCRQDKEGREITDNTLKKIYYVSQPEQK
Dm        QKGTEYVTISCS------TNDPLTIYAGTSIGTIREFQDST---------------------------------LVREITI

          892
TtFAP57C  TQIIKNYHISSFTFNKCIYNTTGIIAGTSTG--QIRVYPSLFAQQPFDSIP-------------THNGQVSHIIASKDGRF
Hs        SAFDVTYTAIVISHSGRMMF-VGTSVGTIRA-----MKYPLPLQKEFNEYQ-------------AHAGPITKMLLTFDDQF
Xt        PSFDVTYTAVAVSHSGRMIF-TGTSLGTIRS-----MKYPLPLQKEFNEYQ-------------AHAKPVTRLVVTFDDQY
Dr        SSRDVVCTTVALSRSGRTIF-IGTSTGTLRV-----IKYPLMIQNDWIEYQ-------------AHAGPITKMVITFDDQF
Che       DAGTNLTQVGLMPNAKVMFA--ATESGALRT-----YKFPLT--GEFQEIK-------------SHHSSITRLRMTWDEQL
Tc        DLQNASLYSMVISPKLKLLI-GGAGDGTVRV-----FNTYLGEKLSGMERERGNGGEIMIESHNAHSGIVTRLALTFDESL
TtFAP57D  KLLDINLSCLAFPSSNKLLF-AGIQDDARSSGAIRCFIYPLTH-GKFTDYQ------------AHDERGVEKMKITNDDRY
TtFAP57B  INQEVQLNQIAFTSTNKIMF-AGIADEQRSSGAVRCILFTTPTNNKFYDYP------------AHDEQGIEKLRITYDDKY
TtFAP57A  IFTGTNVSQIAISHSKKLFF----FATEDRPGAIRITKYPFTN--EIMEIQ------------SHF-GPITRMRISFKDNY
Dm        PSRTKGSVSDVCLARSDLIM----FVADHEGNLFNMQLPFLEAGGGTFTNFR-------------FFDGPVNKLRFSYDGTL
```

```
                 973
TtFAP57C  LFTGGKDGSVFIFKVSELNAEGHSIKADQNDTAIAVDEKLADVVLVERQEIDVYLGEQKKLKEEYENLENKMEIQALEEKN
Hs        LLTAAEDGCLFTWKVFDKDGRGIK-RERE---------VGFAEEVLVTKTDMEEKAQVMLELKTRVEELKMENEYQLRLKDM
Xt        LLSVSEDGCLILWKISDKEGRGLK-RDKE---------VGYAEEVLITKSDLEEKNQVMLELKTRVEELKMENEYQLRLKDM
Dr        LITVSEDGCLFIWKIIDKEGQGLK-REKE---------LIYSEEILTNKLDFDEKSKMLLELNAKVEELQEDNKCQLSLKDT
Che       LVTASDDGCIFVYDVKDKDAKAAARRDQER--------MDWAVEVLVTRSELDEKKSRMSELEQQVAELTMQTEYQLRLKDL
Tc        VVSVGEDGAVIFWDVVAPYRGPHKE-------------VEYSREIFMANSDLEESTKTVSALEAEVNELKQRMQQQQIKRDR
TtFAP57D  IITAGKDGCIMVFEIKDKDARGMKLKDGYA---------KYSEEELITRSDLDDLKSTRDGLIVQINEFSNQNAMIGLNSRD
TtFAP57B  LITAGRDGCIMLFEIKDKDARGMRLKEGYP---------KNAEEILVTRQDLDDLKNTIDNYQSLIGEFNNQTLNNQTNQKD
TtFAP57A  IFTAGEDGALIIYENKEKEYQVKIENESVE---------AAAEEFLIPRDQYNDQKREIEKLKRQLNEERMKQEQQIKEKMK
Dm        LFAISNKGTLAIWAMDNIEGKVPYMDQDLM----------RSQEVLIPRSQLNDKIEQIANLELRLKQQAEEFQYQLSQNEI

                1054
TtFAP57C  KMEREIKEMETKMRTEIKSYEQRYEDLKFQKNRNEKEHGGLLKELEKTHLKAIEELENLYEKKLAFENEKFLQQEQELIEE
Hs        NYSEKIKELTDKFIQEMESLKTKNQVLRTEKEKQDVYHHEHIEDLLDKQSRELQDMECCNNQKLLLEYEKYQELQLKSQRM
Xt        NYNEKLKELTEKFIQEMEVLKTTNQILKTDSERQDIKHQEDVAELLEKQSREIQDLETSNSQKLLLEYEKYQELQLKSQRM
Dr        EYNKKINELSEKCMQQIISLNAEKEVLKKEKDNQQEAHNKAISDILERHDKELHDMESSNNQKLMLEYEKYQELQLKLHNV
Che       HLQERVKELTDKFTAELEADRQKFELLLQEKNEQEMEYEEKLKLAEERSQVQLSTLDTQYQAKIMAEVERFQQLTQEKELL
Tc        VHEEQLLKMEQEYADEQARRLAQYEALKADKNEQAIQFTDFMAEMEAKEKEALKRTKEDYSVKIQSLRDRADRLRQLIDEI
TtFAP57D  DRIRQLETQIENNSQKRKQMFEQLLKSKTMEEQKLIQEINEIKEQFEQEIQVMDTKYQKEVMSLVEEYENQKRLHEIENNK
TtFAP57B  EQIKQLQERLQKSLEHNKTAYEQLQEKKRDIEKKYDEELKIMKEKFEAEIQELDTSYQRKVMDEVEKHESVKKVHEIQRNK
TtFAP57A  DRDDKINQLENQQKDSDNRDLNKYQLLEREKNEMIESYEEKKRMMKLQHENNKRTIENEYKKKIALEMSRNEELAREKEKE
Dm        FDGQQLQEVHRSYCSALEELKELNNEIEARHTEEMNHITFQINSIREEHRIQLDTLATQYSERMLIEYQKFTNLRENMLEL

                1135
TtFAP57C  RMKFEKKLKEIERKHDDNINSLKYEFNDNFQKAQKVYDSTKQTADDLRKIYEERLAQQEEEHEQEIRDLNENHKKEITKLV
Hs        QEEYEKQLRDNDETKSQALEELTEFYEAKLQEKTTLLEEAQEDVRQQLREFEETKKQIEEDEDREIQDIKTKYEKKLRDEK
Xt        QEEYEKQLHELEESKIQALEEITEHYEAKLQEKMSVLQLTQDESRQHLREFEETKKQIEEDGDREIQDMKIRYERRLREEK
Dr        QQEYEQQLHSMEESKTQALEDMMQSYEAKMEEKMILVNQYQDESQQRFKEFEEHIRQEEEDTDAEIHDIRMKYEQNLREEK
Che       NERWDEQNSLLVESHERVIAELTEDYEAKLAEESLKIEQLQQEKAELEREFEEIKRQLEEDADREIEELKEKYESKLATER
Tc        QHEQDAHLAEVREAAAKKRAEDNERQRKVLKELEEENHQLLMERDEDTKRTENYCALIEDETDVEVTLLKEDVEKRRKEQE
TtFAP57D  NNKKKTKLLQEHSQKLQIIDDQYQKLLEEQITQKERV---EKQINHLQKEQQEVLMQISEEKETEIKNLNQKNSKDEQAIT
TtFAP57B  NNRERQQKAMQCQMQAQSLDQEYQQKLHEERSQRERV---QNDIDKMQKDNDEIYRQIEQETKEEIENLNNKNLEHEAEVK
TtFAP57A  EKR--FQSEIQQYQEEHLRQMEEKRRHYEEQLAIEK----QLYNDLHLKREELAYKFDQKRNKLEMEAEELIDQLKEENES
Dm        RESYEDKLKNSTGTLQDTVEALENNYKQQLNERKELIRDLMKEMQDKKAEFIEYCREVELENDRNMVSTQTEYENKLTTER

                1216
TtFAP57C  KDLALAEEKKNKFDEDNKKAKENITRLDKKIEDQDN------EIQYQKSRLTDASNQIEALKKDIISLEDLVKKKEKKIHE
Hs        ESNLRLKGETGIMRKKFSSLQKEIEERTNDIETLKG------EQMKLQGVIKSLEKDIQGLKREIQERDETIQDKEKRIYD
Xt        ESSLRLKGESGIMRKKFSSLQKEIEERAADIEKMKA------EQQKLQGVIKSLEKDILGLKREIQERDETIQDKEKRIYD
Dr        KAHSKLKVELDTTKKRFHDLQREIENGNLEIEKLKQ------EVQRLQAVIKSLENDITGLKTAIQNRDGIIQDKEKNIFQ
Che       ESSLRLKGENGIMRKKFNALQKDIEAQKEEIKNLFE------QKRELYATIASLEKDIASLKREIRERDETIGDKERRIYD
Tc        EEVATLQTNNDLLLSRENIIKAELEAVKADVREKLR------QQSTLESQIEAAKRDIGALTQEFKDRGETIAEKERRVLD
TtFAP57D  DQGLKAKSDISITKKKIMQQQQEAQDLKEQKQEYER------QKEKLKIQNQELRDKIDGQKKIILERDRTIGEKEKIIYN
TtFAP57B  ERHKKAKSDAAITTKKIDQKKKDSEQYEEQFRLHQK------QLKKLREENETLLKEIQQQKEIIQERDKTIGKQEKQIYK
TtFAP57A  KMQVLFKNLEKAEIKKMDRRNDYDTEAQKLEEQKSKLKGTMEDITSIQETNKMLQKEKESHAKEIDEREKTIRDKGRRIYE
Dm        NETQMWRGKAGVLQKKFESQSREIDNLLEEVEILKE------EHHKSQRNIQKQMRNIEDLQKDIADRDYAINGKEKRIQD

                1297
TtFAP57C  YKYKINDLQKSKHVLSFRTTEMRKSLEPKEEQIEKQKEQLCKLESEFEGLLKVSEARNDKMKKMQSQIENLNKNLKLQTEL
Hs        LKKKNQELGKFKFVLDYKIKELKKQIEPRENEIRVMKEQIQEMEAELENFHKQNTQLELNITELWQKLRATDQEMRRERQK
Xt        LKKKNQELEKFKFVLDYKIKELKKQIEPRENEIKEMKEQIQEMESELERFHKQNSQLDLNIAELKQKLKATDREMHKERQK
Dr        LKKKNDTLNKTLEVMEFRAEYMQEQIEPKENEIQQQKDQMVECFMKPHFAHH-----------------------------
Che       LKKKNQELEKFKFVLDYKIKELKKQIEPREQEISDMKEQIKEMDGELERYHKTNSGLDLLISNLKLKQEGLQQEVMTQRTG
Tc        LKKKNQELEKFKFVLEYKIKELKLQIDPRDEEIRQAKSRLSEMGKEADNYTHSNEHLVLQIRNLRHKKAGQQKELDKLAEI
TtFAP57D  LKKKAQDLEKFKFVLDHKIKELKRDIVPREDEITKMKQETNNMDQYLKELNAYNNYLGTVVDELYTTQETMKEDIKQQRQQ
TtFAP57B  QKKKSQNLEKFKFVLDHKIKELKRDIGPREDEIARMKEETNQMDQLLKKYNSYNNHLGNAVDELYTAQELMNSEIKNQRTV
TtFAP57A  LKKKTQELEKFKFVLDYKIKELKRDIGPKEEEIAKMKEQIANMNSEILHFKRTNANLKLIVTDLRLRQEGMKKEIEDQSKV
Dm        LLHKNQELDKYKQVLGHKIAELKAQIEPREFQINDKRKHIIEMEAELEGLNQNNVQLELQLKEMRDKYLSNVAELRTERHR

                1378
TtFAP57C  TKQKENILNKIIMEIHDSVTYKQDPK-ELAQEMQKLYQTYVLKT----EHKQKQ-YDNKGVQEMGRQIQHLNKTIQQISES
Hs        ERDLEALVKRFKTDLHNCVAYIQEP-RLLKEKVRGLFEKYVQRADMVEIAGLNT----DLQQEYTRQREHLERNLATLKKK
Xt        VRDIEAVVKRFKTDLHNCVGFIQEP-KMLKDNIRELYSKYVQDSDVAEIVGVDA----DIQREYARQREHLERSLSTLQKK
Dr        ---------------------------------------------------------------------------------
Che       KSDAEQLLKRMQHDLQAIIPYFQEP-KDLKEQVKALYHKYCGNQPLRTEEDADL------EREAARQREYLEKTVDTLKRK
Tc        MRGFAEYLSRLWTELSCLREETNP--RKLKECAKMLFDKYTSTKAVAANKTLASRTAADEVREYNRERDHLERNLAGLKNK
TtFAP57D  ISVQQVKISRFKDDVYSLAQHILDYD-KLVDETERLFMKHVNDKEVKRQSVEGD-----IMMEYRSQKKNLEKLVNMFKKS
TtFAP57B  ISSTNNKIKRFKDDLYKAVQHIQDYE-KLQQHVNELKRLYANKNIERNKNDEQI------LQEYENQKAHLQYKVQRLKKY
TtFAP57A  IQQNNQYIKAFEQDMSDCHQHIADYK-KLKQKVLNLYNQYVQGDDSKKKRLENN----DQQKEIMKERAHLETSVNNLKIK
Dm        AKASRECLHSICSDIYYVAGEINSAE-ALKKAVKELFRKHASDDELKRFVTLDA----EVRDEFMRQRKQIENVLDRYKSV
```

```
          1459
TtFAP57C  SNKLLDRRYQDIFNKTKENAELIYDLNDMRKQNKEFQSQILNLRMKEDKYIKEIANLKTEIQKLKLGGPGGSSKKNNNMMQ
Hs        VVKEGELHRTDYVRIMQENVSLIKEINELRRELKFTRSQVYDLEAALKLTKKVRPQEVSETEPSR-DMLSTAPTARLNEQE
Xt        LAKDTEIHRTDNVRVMQENVTLIKEINDLRRELKLSRTKVHDLEAILGLNKKTRKNNATELKVSSSEGLHPVGVLRLNSEE
Dr        ---------------------------------------------------------------------------------
Che       LAKDSELHRTDNLRIMQENTALIKEINELRREIRTLKSRVAGINGAVNLSVTGRRPGSPGDSLMLSEGLKRELEMQRDLIT
Tc        VNKDAENNRSDKNRITTENVILIREINDLRKEARRLAEKAGAWRKTEKCAASLQQEETKRELAMQRAELKRLRALARKLAE
TtFAP57D  LQKDNQIHKDDKIRIMKDNVDLIREINTLRKSIKDITKGQQSNIPDQKGQTQHMPLQTPQLSRKSISQPVLPPISKSAMFE
TtFAP57B  LKQDNQIHKDYNLGLMSNNVNLIKEINILRKQIKDIQKGEGGQTGKPAEHARSLTTSFKRARTAGVRSNSKNGNRPDEIDE
TtFAP57A  HDKNQSVHKSDTTIIMKHNTFLIFEINHLKREKKKILEDKAKMLMQATQKKKQDGTSRVDIDSLEKEIQKNEDEKRKLKED
Dm        AEDKSVQKKYD--KLFKENVILIEEIEKLNETNKMLRSKVKEDLRRSTKTIHK----------------------------

          1540
TtFAP57C  LPPAQQMQQQQYDEQRQNVNSVQQTQRNNFFSQSQGQVQSNQRPQSMKIGQKGTICRGKNFDTKTLAIFDKAKMMDMTAEM
Hs        ETGRIIEMQRLEIQRLRDQIQEQEQVTGFHTLAGVRLPSLSNSEVDLEVKTN-----------------------------
Xt        ETDRIIDMQRLEIKRLRDLIQSQELTQGVRPPSVGRLPALTMTTDRGIREFPWDR--------------------------
Dr        ---------------------------------------------------------------------------------
Che       RLREETAAKEARIRQLESMIAPRPTSRERLPPMEGFPGSMPPERISSSLLMKGESRPPSAAIDLPPGIMEQEAVA------
Tc        EAREKGITLTLPSLTEGRPGS------------------------------------------------------------
TtFAP57D  GVTQQNEIQKSHTQINPELVEEKQKVLNSLQTDILELQKVYEALNYEKHNLTQA---------------------------
TtFAP57B  ALNDPAIQQKQQILVMQSDDITQLRVQLEELHEENRALTVSNNF-------------------------------------
TtFAP57A  IEKLRQYNDQIKNQLRQQIQNQQDDDEDN----------------------------------------------------
Dm        ---------------------------------------------------------------------------------

          1621
TtFAP57C  ELNTQKLLQQEVLIKSIRRKIHKHVIDRACNDIAFEEELMALQAQEQGGFIPLQQQDIDTSDDNIYQAEQRQYSRK
Hs        ----------------------------------------------------------------------------
Xt        ----------------------------------------------------------------------------
Dr        ----------------------------------------------------------------------------
Che       ----------------------------------------------------------------------------
Tc        ----------------------------------------------------------------------------
TtFAP57D  ----------------------------------------------------------------------------
TtFAP57B  ----------------------------------------------------------------------------
TtFAP57A  ----------------------------------------------------------------------------
Dm        ----------------------------------------------------------------------------
```
