# Supplementary material for: Ciliary proteins Fap43 and Fap44 interact with each other and are essential for proper cilia and flagella beating
Source: Cell Mol Life Sci. 2018 Apr 23;75(24):4479–93. doi: 10.1007/s00018-018-2819-7 (PMC6208767; doi:10.1007/s00018-018-2819-7)
Supplement: Supplementary file 12 — List of used primers. (DOCX 31 kb) [file 18_2018_2819_MOESM12_ESM.docx]

**Table S1**

List of used primers.

| **name** | **nucleotide sequence** |
| --- | --- |
| **native locus expression with C-terminal tag** | |
| FAP43-MluI-F | AATT **ACGCGT** TATGCCTTTGAAGAATAGAAGAAG |
| FAP43-BamHI-R | ATTA **GGATCC** GTATTTTTAGACTGCAGTGAAGTTAGC |
| FAP43-PstI-F | ATAT **CTGCAG** TTTTAAGAGTTATGGTTTAATGAATACAA |
| FAP43-XhoI-R | AATT **CTCGAG** TAGTATTATCAACTAAAAATCAGTGGA |
| FAP44-MluI-F | AATT **ACGCGT** TAGGTATTGGAAATGAGAGAAAAG |
| FAP44-BamHI-R | ATAT **GGATCC** GTTACTTCTAATAAAAAGAATTTTAAT |
| FAP44-PstI-F | ATAT **CTGCAG** TATATGTACTTACTTGTCCAATA |
| FAP44-XhoI-R | AATT **CTCGAG** GTAATAAGTGAGAGAAATTGTCAA |
| FAP57A-MluI-F | AATT **ACGCGT** GATGGACAGAAGAAACGACTATG |
| FAP57A-BamHI-R | AATT **GGATCC** ATTGTCTTCGTCATCGTCTTACTG |
| FAP57A-PstI-F | AATT **CTGCAG** TCATACATAATCAGTTAGTTAATTGATTCTAC |
| FAP57A-XhoI-R | AATT **CTCGAG** TGGCGACTATTTGCTTCTTC |
| **native locus expression with N-terminal tag** | |
| FAP44-SacII-F | TTAA**CCGCGG**TATAGGCAGAGGAGTTTGGAA |
| FAP44-Pst-R | TTAT**CTGCAG**GGTTGTGCTAAATGTATAATTATCCTATC |
| FAP44-MluI-F | AATT**ACGCGT**CATGAGTGATTAAGGTTTAAACGATTAAC |
| FAP44-BamHI-R | AATA**GGATCC**TTCACCCTTTAGCTTCAAACC |
| **overexpression in *BTU1 locus*** | |
| FAP43-MluI-oex-F | ATAT **ACGCGT** CATGAGTATTTAGTATAGCTTGTTTAG |
| FAP43-BamHI-TGA-oex-R | ATT**GGATCC**TCACCATACTTTAACCCATGC |
| FAP43-BamHI-oex-R | ATTA **GGATCC** GTATTTTTAGACTGCAGTGAAGTTAGC |
| **domain analysis** | |
| FAP43 D1353 R | AATT **GGATCC** CATCAACATATGAGTCGAATATTCCATC |
| FAP43 K712 R | AATT **GGATCC** TACCTTTTTGATAATGCATAACTTTTTC |
| FAP43 G667 F | AATT **ACGCGT**TATGGA TGGTTCATTCTTTGCCTGGAGTC |
| FAP44-wd-R | AATT **GGATCC** TATGCTTTCAGGGTCCCATTCAACTTCTTC |
| **gene knockout** | |
| FAP43 upstream-ApaI-F | TTAT **GGGCCC** TAGGAGGAGCTCCGA |
| FAP43 downstream-SmaI-R | TAAT **CCCGGG** TCCTGCATCGTCTTGCTTG |
| FAP43 upstream-PstI-F | AATA **CTGCAG** GCCTTTGAAGAATAGA |
| FAP43 downstream-SacII-R | AATA **CCGCGG** CCTAGTTCTTCACGCAA |
| FAP43-coDel-F | CAGTTCTCATCAAGTTGTAATGCTAAAAT**GCGGCCGC** CAC AAT ATT TGA TTA AAC AAA AAA TCA TTT AGA |
| FAP43-coDel-R | GGACTCTTTATTGTTATCATCTTATGACC**GCGGCCGC**  ctcgaGAACTTCTTAAGAGTTTTAGTCTACTTC |
| FAP44-coDel-F | CAGTTCTCATCAAGTTGTAATGCTAAAAT**GCGGCCGC**  ATGAGTGATTAAGGTTTAAACGATTAACAATAAG |
| FAP44-coDel-R | GGACTCTTTATTGTTATCATCTTATGACC**GCGGCCGC**  GATTAACAGCTATGCTGCCTATAC |
| DYH6-coDel-F | CAGTTCTCATCAAGTTGTAATGCTAAAAT**GCGGCCGC** TTTATTCATTAAACTTCATTATGACTTGTATTT |
| DYH6-coDel-R | GGACTCTTTATTGTTATCATCTTATGACC**GCGGCCGC**  ctcgagCATAAGGTCAACATTTTTAATTGC |
| DYH7-coDel-F | CAGTTCTCATCAAGTTGTAATGCTAAAAT**GCGGCCGC**  GTAAATTAGTATGTGCTCTATACTTTTCTA |
| DYH7-coDel-R | GGACTCTTTATTGTTATCATCTTATGACC**GCGGCCGC**  ctcgagTAAGAAATTTATTAAACTCCTCTTTTGTT |
| **PCR analysis of the targeted loci** | |
| FAP43-KO-F | GGAGCATTTGAATTGTTTACAAAC |
| FAP208-F | ATGATTGCTGATGATTTTGAAC |
| FAP208-R | GACGTCAATAGAAAGATAATGTGCGTTTCA |
| FAP43-F | CAGGAAAAGGTGGTGTCGCAATG |
| FAP43-R | CCA TCT TAT TCT TCA ATC GGA GCT CCT |
| FAP44-F | AATTACGCGTCTGAGCTTCATAGAGCTAAATGGTGA |
| FAP44-R | AATTCTCGAGTATATGGAAGCCACTCTGCACATG |
| DYH6-F | GGCTACTTCTTCATCATCAACTTCACC |
| DYH6-R | GTG ACA GCC TTT AGA TTG GGA CC |
| DYH7-F | CGAGATTATGAGACTCGCGCTCTA |
| DYH7-R | ATT AGG AAG CCT CGG TGA GAG TAG |

Nucleotide sequences recognized by the restriction endonucleases are in bold, and additionally introduced restriction sites used in screen for positive clones are indicated by small letters.
